# Supplementary material for: C. elegans RIG-I-like receptor DRH-1 signals via CARDs to activate anti-viral immunity in intestinal cells
Source: bioRxiv. 2024 Feb 8:2024.02.05.578694. Preprint. [Version 1] doi: 10.1101/2024.02.05.578694 (PMC10871272; doi:10.1101/2024.02.05.578694)
Supplement: Supplement 1 [file media-1.pdf]

S1A Table. Foldseek analysis

No.	Target (PDB ID)	E-value	TM-score	Alignment length	Sequence identity (%)	Description	Organism
1	4p4h.cif.gz_C	3.84E-01	3.59E-01	204	13.7	Caught-in-action signaling complex of RIG-I 2CARD domain and MAVS CARD domain	*Homo sapiens*
2	6e25.cif.gz_MODEL_10_A	3.52E+00	6.28E-01	103	14.5	NMR solution structure of the CARD9 CARD bound to zinc	*Homo sapiens*
3	6e25.cif.gz_MODEL_3_A	3.05E+00	5.68E-01	98	14.2	NMR solution structure of the CARD9 CARD bound to zinc	*Homo sapiens*
4	1wxp.cif.gz_MODEL_1_A	3.69E+00	5.66E-01	94	12.7	Solution structure of the death domain of nuclear matrix protein p84 (THOC1)	*Homo sapiens*
5	6e25.cif.gz_MODEL_4_A	5.38E+00	5.36E-01	97	14.4	NMR solution structure of the CARD9 CARD bound to zinc	*Homo sapiens*
6	7dni.cif.gz_B	3.36E+00	3.69E-01	220	8.1	MDA5 CARDs-MAVS CARD polyUb complex	*Homo sapiens*
7	2n7z.cif.gz_MODEL_2_A	8.61E+00	5.89E-01	85	16.4	Solution structure of RIP2 CARD	*Homo sapiens*
8	6gwm.cif.gz_MODEL_3_A	9.92E+00	5.17E-01	90	13.3	Solution structure of rat RIP2 caspase recruitment domain	*Rattus norvegicus*
9	5uxv.cif.gz_B	5.38E+00	3.34E-01	116	11.2	Crystal Structure of Anti-anti-sigma factor PhyR I40V/S51C mutant from Bartonella quintana	*Bartonella quintana str. Toulouse*
10	7ml4.cif.gz_M	4.89E+00	2.59E-01	247	11.7	RNA polymerase II initially transcribing complex (ITC)	*Saccharomyces cerevisiae*
11	4g97.cif.gz_A	9.92E+00	3.02E-01	103	11.6	Crystal structure of the response regulator PhyR from Brucella abortus	*Brucella abortus 2308*
12	1h0o.cif.gz_A	3.69E+00	1.52E-01	237	8	Cobalt substitution of mouse R2 ribonucleotide reductase to model the reactive diferrous state	*Mus musculus*
13	8c3w.cif.gz_A	8.61E+00	1.69E-01	241	13.2	Crystal structure of a computationally designed heme binding protein, dnHEM1	Synthetic construct
14	8g0c.cif.gz_d	6.19E+00	1.52E-01	258	13.5	Cryo-EM structure of TBAJ-876-bound Mycobacterium smegmatis ATP synthase rotational state 1 (backbone model)	*Mycolicibacterium smegmatis MC2 155*
15	3g33.cif.gz_B	9.92E+00	1.53E-01	247	10.5	Crystal structure of CDK4/cyclin D3	*Homo sapiens*
16	3v71.cif.gz_A	8.61E+00	1.66E-01	212	15	Crystal structure of PUF-6 in complex with 5BE13 RNA	*Caenorhabditis elegans*
17	3e4a.cif.gz_B	9.92E+00	1.92E-01	85	17.6	Human IDE-inhibitor complex at 2.6 angstrom resolution	*Homo sapiens,* synthetic construct
18	5ivw.cif.gz_2	7.13E+00	1.44E-01	270	11.1	Human core TFIIH bound to DNA within the PIC	*Homo sapiens*
19	6ivn.cif.gz_C	8.61E+00	1.37E-01	217	12.9	Crystal structure of a membrane protein G264A	*Klebsiella pneumoniae*

S1B Table. Dali analysis

No	Chain	Z	rmsd	lali	nres	%id	Description
1	6bzh-B	8.9	6.8	104	192	9	MOLECULE: PROBABLE ATP-DEPENDENT RNA HELICASE DDX58;
2	5juy-B	8.8	13.5	112	1234	10	MOLECULE: APOPTOTIC PROTEASE-ACTIVATING FACTOR 1;
3	6n2m-A	8.7	3.5	103	142	9	MOLECULE: CASPASE RECRUITMENT DOMAIN-CONTAINING PROTEIN 9;
4	7dni-A	8.7	2.6	86	199	13	MOLECULE: MDA5 CARD;
5	6xkj-A	8.2	2.7	82	85	10	MOLECULE: CASPASE RECRUITMENT DOMAIN-CONTAINING PROTEIN 8;
6	2of5-H	8	2.8	89	100	6	MOLECULE: DEATH DOMAIN-CONTAINING PROTEIN CRADD;
7	6m6q-B	7.9	5.1	176	315	9	MOLECULE: DICER-RELATED HELICASE 3;
8	6mks-A	7.8	2.9	85	90	9	MOLECULE: CHIMERA PROTEIN OF NLR FAMILY CARD DOMAIN-CONTAIN
9	4d8o-A	7.7	4.1	80	509	11	MOLECULE: ANKYRIN-2;
10	6gwm-A	7.6	4.3	111	125	11	MOLECULE: RECEPTOR-INTERACTING SERINE/THREONINE-PROTEIN KIN
11	2yqf-A	7.6	5.2	96	111	16	MOLECULE: ANKYRIN-1;
12	4e9m-A	7.5	2.8	79	97	6	MOLECULE: NUCLEOTIDE-BINDING OLIGOMERIZATION DOMAIN-CONTAIN
13	5wvc-B	7.5	2.9	89	103	12	MOLECULE: APOPTOTIC PROTEASE-ACTIVATING FACTOR 1;
14	6ac0-A	7.5	3.8	92	115	11	MOLECULE: TUMOR NECROSIS FACTOR RECEPTOR TYPE 1-ASSOCIATED
15	2mb9-A	7.2	3.4	96	106	8	MOLECULE: B-CELL LYMPHOMA/LEUKEMIA 10;
16	4m9s-B	7	15.5	131	511	9	MOLECULE: CELL DEATH PROTEIN 4;
17	4dwn-A	6.8	2.8	87	97	9	MOLECULE: BCL10-INTERACTING CARD PROTEIN;
18	2gf5-A	6.8	3.8	93	191	12	MOLECULE: FADD PROTEIN;
19	3t6p-A	6.8	3.5	95	330	11	MOLECULE: BACULOVIRAL IAP REPEAT-CONTAINING PROTEIN 2;
20	4uz0-A	6.7	2.7	80	87	11	MOLECULE: NUCLEOLAR PROTEIN 3;
21	1ich-A	6.5	2.9	79	87	15	MOLECULE: TUMOR NECROSIS FACTOR RECEPTOR-1;
22	2o71-A	6.5	3.3	86	91	9	MOLECULE: DEATH DOMAIN-CONTAINING PROTEIN CRADD;
23	1wxp-A	6.4	4.6	93	110	11	MOLECULE: THO COMPLEX SUBUNIT 1;
24	7cmg-A	5.6	10.8	83	487	8	MOLECULE: POR SECRETION SYSTEM PROTEIN PORM/GLDM;
25	1ddf-A	5.5	4.8	106	127	12	MOLECULE: FAS;
26	7k3r-A	5.5	3	78	95	13	MOLECULE: INTERFERON-INDUCIBLE PROTEIN AIM2;
27	3mop-K	5.4	2.9	77	93	9	MOLECULE: MYELOID DIFFERENTIATION PRIMARY RESPONSE PROTEIN
28	2km6-A	5.4	3.1	79	96	13	MOLECULE: NACHT, LRR AND PYD DOMAINS-CONTAINING PROTEIN 7;
29	1ygo-A	5.2	3.1	86	110	7	MOLECULE: PROBABLE SERINE/THREONINE-PROTEIN KINASE PELLE;
30	7bso-A	5.2	3.1	78	90	4	MOLECULE: NACHT, LRR AND PYD DOMAINS-CONTAINING PROTEIN 9;
31	2mjm-A	5.2	3.4	82	101	11	MOLECULE: PROTEIN NLRC5;
32	6jcc-A	5.2	3.3	79	97	13	MOLECULE: COMPUTATIONAL DESIGNED PROTEIN BASED ON EVOLUTION

33	3mop-A	5	3	80	105	9	MOLECULE: MYELOID DIFFERENTIATION PRIMARY RESPONSE PROTEIN
34	3qf2-B	4.9	2.9	73	106	12	MOLECULE: NACHT, LRR AND PYD DOMAINS-CONTAINING PROTEIN 3;
35	6ncv-A	4.9	2.8	72	91	13	MOLECULE: NACHT, LRR AND PYD DOMAINS-CONTAINING PROTEIN 6;
36	2dbg-A	4.8	3.3	82	103	6	MOLECULE: MYELOID CELL NUCLEAR DIFFERENTIATION ANTIGEN;
37	2ms7-A	4.8	3	82	102	13	MOLECULE: MITOCHONDRIAL ANTIVIRAL-SIGNALING PROTEIN;
38	4zbw-A	4.7	4.1	83	183	5	MOLECULE: CASPASE-8;
39	6eqt-A	4.6	7.8	79	207	9	MOLECULE: CENTROMERE PROTEIN N;
40	2dbh-A	4.6	3.6	84	102	8	MOLECULE: TUMOR NECROSIS FACTOR RECEPTOR SUPERFAMILY
41	5z8q-A	4.6	6.2	67	101	4	MOLECULE: HEAT SHOCK PROTEIN SSA1;
42	6hj7-A	4.5	4	83	102	10	MOLECULE: DEATH DOMAIN-CONTAINING MEMBRANE PROTEIN NRADD;
43	6ajf-A	4.5	5.3	100	901	7	MOLECULE: DRUG EXPORTERS OF THE RND SUPERFAMILY-LIKE PROTEI
44	1e7p-C	4.5	9.9	72	254	8	MOLECULE: FUMARATE REDUCTASE FLAVOPROTEIN SUBUNIT;
45	5xn7-B	4.4	10.9	90	551	8	MOLECULE: PUTATIVE RTX-TOXIN;
46	2n00-A	4.4	2.9	80	95	10	MOLECULE: INTERFERON-INDUCIBLE PROTEIN AIM2;
47	6irw-A	4.4	12.9	78	495	8	MOLECULE: PHOSPHORYLATED CTD-INTERACTING FACTOR 1;
48	3crd-A	4.4	3.8	89	100	12	MOLECULE: RAIDD;
49	6if2-A	4.3	4.4	101	195	8	MOLECULE: RAS-RELATED PROTEIN RAB-35;
50	1irx-B	4.3	8.9	73	508	10	MOLECULE: LYSYL-TRNA SYNTHETASE;
51	1sxj-D	4.3	10.9	76	328	12	MOLECULE: ACTIVATOR 1 95 KDA SUBUNIT;
52	1gzs-B	4.3	10.9	69	165	1	MOLECULE: SOPE;
53	6e6y-A	4.3	20.1	79	268	10	MOLECULE: DIECKMANN CYCLASE, NCMC;
54	1xfi-A	4.3	5.3	79	343	10	MOLECULE: UNKNOWN PROTEIN;
55	6ybw-z	4.3	3.5	95	160	11	MOLECULE: 40S RIBOSOMAL PROTEIN S4, X ISOFORM;
56	1k30-A	4.3	8.7	60	363	7	MOLECULE: GLYCEROL-3-PHOSPHATE ACYLTRANSFERASE;
57	4xom-A	4.3	10.3	66	206	14	MOLECULE: COENZYME F420:L-GLUTAMATE LIGASE;
58	7lxc-A	4.3	2.8	70	88	10	MOLECULE: DED1CH;
59	2bbz-A	4.3	4.7	85	190	8	MOLECULE: VIRAL CASP8 AND FADD-LIKE APOPTOSIS REGULATOR;
60	6poo-A	4.2	10.9	108	273	4	MOLECULE: BIBA;
61	5b4s-B	4.2	10.8	56	310	9	MOLECULE: CHITOSANASE;
62	3p8c-B	4.1	11.3	87	1086	5	MOLECULE: CYTOPLASMIC FMR1-INTERACTING PROTEIN 1;
63	5v8f-D	4.1	12.5	87	456	5	MOLECULE: DNA REPLICATION LICENSING FACTOR MCM2;
64	6jal-A	4.1	11	69	416	10	MOLECULE: ABC TRANSPORTER, PERIPLASMIC SUBSTRATE-BINDING PR
65	5yet-A	4.1	10.6	62	397	15	MOLECULE: UNCHARACTERIZED PROTEIN R354;
66	2g8l-B	4.1	3.7	70	286	13	MOLECULE: 287AA LONG HYPOTHETICAL PROTEIN;
67	6vvo-A	4.1	13.5	62	448	6	MOLECULE: REPLICATION FACTOR C SUBUNIT 1;

68	6pie-A	4.1	2.8	54	162	13	MOLECULE: HEMERYTHRIN HHE CATION BINDING DOMAIN PROTEIN;
69	7bvf-A	4.1	10	92	1087	5	MOLECULE: PROBABLE ARABINOSYLTRANSFERASE B;
70	5izs-D	4.1	4.1	47	82	9	MOLECULE: DESIGNED PROTEIN 5L6HC3_1;
71	2d96-A	4	4.6	84	109	13	MOLECULE: NUCLEAR FACTOR NF-KAPPA-B P100 SUBUNIT;
72	4elj-A	4	11	76	590	5	MOLECULE: RETINOBLASTOMA-ASSOCIATED PROTEIN;
73	6zg3-D	4	3.3	53	260	4	MOLECULE: ENERGY-COUPLING FACTOR TRANSPORTER ATP-BINDING PR
74	2g7r-A	4	3.3	66	86	11	MOLECULE: MUCOSA-ASSOCIATED LYMPHOID TISSUE LYMPHOMA TRANSL
75	6j72-A	4	3.3	57	561	7	MOLECULE: ISONIAZID INDUCIBLE GENE PROTEIN INIA;
76	3u5z-B	4	13.3	66	320	9	MOLECULE: DNA POLYMERASE ACCESSORY PROTEIN 44;
77	6ny2-Y	4	13.1	123	915	5	MOLECULE: DNA TARGET STRAND;
78	6hsy-A	4	5	58	190	12	MOLECULE: TOLUENE TOLERANCE PROTEIN TTG2D;
79	1wh4-A	4	3.6	87	127	8	MOLECULE: INTERLEUKIN-1 RECEPTOR-ASSOCIATED KINASE 4;
80	1d2z-D	3.9	8.3	94	153	10	MOLECULE: DEATH DOMAIN OF PELLE;
81	2hjm-A	3.9	2.9	60	91	8	MOLECULE: HYPOTHETICAL PROTEIN PF1176;
82	5way-A	3.9	5.3	64	482	5	MOLECULE: TRANSCRIPTIONAL ACTIVATOR;
83	5f3o-A	3.9	3.5	59	194	5	MOLECULE: PUTATIVE UNCHARACTERIZED PROTEIN;
84	7c6g-A	3.9	10.2	60	420	3	MOLECULE: SUGAR ABC TRANSPORTER, PERIPLASMIC SUGAR-BINDING
85	5wb2-A	3.9	5.9	75	421	5	MOLECULE: ENVELOPE PROTEIN US28, NANOBODY 7 FUSION PROTEIN;
86	6jpq-A	3.9	9.4	60	577	12	MOLECULE: UNCHARACTERIZED AAA DOMAIN-CONTAINING PROTEIN C31
87	4mhq-A	3.9	10.9	102	400	7	MOLECULE: DNA PRIMASE SMALL SUBUNIT;
88	6fze-A	3.9	4.9	59	209	5	MOLECULE: PUTATIVE SURFACE PROTEIN;
89	3add-A	3.9	12.5	57	251	14	MOLECULE: L-SERYL-TRNA(SEC) KINASE;
90	4kbq-C	3.9	8.3	63	91	5	MOLECULE: E3 UBIQUITIN-PROTEIN LIGASE CHIP;
91	6uuw-A	3.9	4.4	68	1034	4	MOLECULE: ATP-CITRATE SYNTHASE;
92	1jgt-B	3.9	11.1	68	501	7	MOLECULE: BETA-LACTAM SYNTHETASE;
93	4aur-A	3.9	5.4	68	564	15	MOLECULE: LEOA;
94	3dpn-A	3.8	4.1	72	537	8	MOLECULE: PROTEIN CT_858;
95	5cwc-A	3.8	7.5	61	196	11	MOLECULE: DESIGNED HELICAL REPEAT PROTEIN;
96	5vyz-A	3.8	13	95	1144	6	MOLECULE: PYRUVATE CARBOXYLASE;
97	2gut-A	3.8	2.7	48	77	4	MOLECULE: ARC/MEDIATOR, POSITIVE COFACTOR 2 GLUTAMINE/Q-
98	5che-A	3.8	3.7	56	427	9	MOLECULE: GLUTAMYL-TRNA REDUCTASE 1, CHLOROPLASTIC;
99	2kn6-A	3.8	3.4	67	195	10	MOLECULE: APOPTOSIS-ASSOCIATED SPECK-LIKE PROTEIN CONTAININ
100	3zjc-E	3.8	12.4	64	286	17	MOLECULE: GTPASE IMAP FAMILY MEMBER 7;
101	4mk6-A	3.8	11.1	67	188	6	MOLECULE: PROBABLE DIHYDROXYACETONE KINASE REGULATOR DHSK_R
102	7ltq-A	3.8	5.8	62	256	6	MOLECULE: HISTIDINE KINASE;

103	6gvw-B	3.8	6.9	55	262	9	MOLECULE: BRCA1-A COMPLEX SUBUNIT ABRAXAS 1;
104	7jzl-E	3.8	3.1	49	55	12	MOLECULE: SPIKE GLYCOPROTEIN;
105	6jbq-F	3.8	4.9	59	186	8	MOLECULE: DNA-DIRECTED RNA POLYMERASE SUBUNIT ALPHA;
106	6gc1-C	3.8	11.7	78	551	10	MOLECULE: NHL REPEAT-CONTAINING PROTEIN 2;
107	7ew1-A	3.8	5.4	68	269	6	MOLECULE: GUANINE NUCLEOTIDE-BINDING PROTEIN G(I)/G(S)/G(T)
108	3kdw-A	3.8	6.2	75	206	7	MOLECULE: PUTATIVE SUGAR BINDING PROTEIN;
109	7kfu-C	3.8	10.4	94	909	6	MOLECULE: CAS2;
110	1nxh-A	3.8	10.8	73	124	14	MOLECULE: MTH396 PROTEIN;
111	3h0d-A	3.8	5.2	72	155	4	MOLECULE: CTSR;
112	3l32-B	3.8	3.2	44	45	7	MOLECULE: PHOSPHOPROTEIN;
113	3cym-A	3.8	7.5	78	415	5	MOLECULE: UNCHARACTERIZED PROTEIN BAD_0989;
114	7s9y-A	3.8	9.4	86	901	3	MOLECULE: CYTOCHROME C BIOGENESIS PROTEIN;
115	1l1l-A	3.7	9.7	85	717	7	MOLECULE: RIBONUCLEOSIDE TRIPHOSPHATE REDUCTASE;
116	2efl-A	3.7	6.9	76	281	3	MOLECULE: FORMIN-BINDING PROTEIN 1;
117	4ayb-Q	3.7	4.2	45	50	11	MOLECULE: DNA-DIRECTED RNA POLYMERASE;
118	2o7g-A	3.7	3.3	47	88	2	MOLECULE: PROBABLE RNA POLYMERASE SIGMA-C FACTOR;
119	6ocv-A	3.7	4.3	76	190	5	MOLECULE: HEME NO BINDING DOMAIN PROTEIN;
120	7cun-E	3.7	12.5	67	796	9	MOLECULE: INTEGRATOR COMPLEX SUBUNIT 1;
121	4akx-B	3.7	10.8	68	534	6	MOLECULE: SPCU;
122	6ibl-A	3.7	4.2	65	397	11	MOLECULE: THIOREDOXIN 1,BETA-1 ADRENERGIC RECEPTOR;
123	6ptm-A	3.7	4.6	66	726	8	MOLECULE: UNCHARACTERIZED PROTEIN;
124	6rmg-A	3.7	10.6	108	1026	10	MOLECULE: PROTEIN PATCHED HOMOLOG 1,GFP-LIKE FLUORESCENT
125	2d1l-A	3.7	8.5	70	249	4	MOLECULE: METASTASIS SUPPRESSOR PROTEIN 1;
126	2y7h-B	3.7	2.4	42	530	2	MOLECULE: TYPE-1 RESTRICTION ENZYME ECOKI SPECIFICITY PROTE
127	6ebu-A	3.7	4.7	77	165	9	MOLECULE: LPXE;
128	7lt5-B	3.7	11.5	65	577	12	MOLECULE: SITE-SPECIFIC DNA-METHYLTRANSFERASE (ADENINE-SPEC
129	6tnt-A	3.7	17.2	93	1583	4	MOLECULE: ANAPHASE-PROMOTING COMPLEX SUBUNIT 1;
130	6mtz-B	3.7	7.2	70	347	9	MOLECULE: HEPC.19480.A.B1;
131	4wl5-A	3.7	5.1	94	594	5	MOLECULE: HETERODIMERIC RESTRICTION ENDONUCLEASE R.BSPD6I L
132	6qgi-A	3.7	2	40	498	10	MOLECULE: VP5;
133	6cng-A	3.7	4.3	63	281	6	MOLECULE: FATTY ACID KINASE (FAK) B3 PROTEIN;
134	1r5i-D	3.7	4.5	67	214	4	MOLECULE: HLA CLASS II HISTOCOMPATIBILITY ANTIGEN, DR
135	6plm-B	3.7	13.5	91	752	8	MOLECULE: SIDJ PROTEIN;
136	3bes-R	3.6	6.2	45	250	13	MOLECULE: INTERFERON GAMMA;
137	5mps-R	3.6	4.5	46	108	15	MOLECULE: YEAST UBC4 GENE FOR UBIQUITIN-CONJUGATING ENZYME;

138	6ep3-B	3.6	9.6	48	218	4	MOLECULE: LMO0651 PROTEIN;
139	4ywz-A	3.6	4.5	70	147	13	MOLECULE: SENSOR PROTEIN KINASE WALK;
140	6ssk-B	3.6	8.4	91	223	8	MOLECULE: ENDOGENOUS RETROVIRUS GROUP K MEMBER 24 GAG POLYP
141	6bog-A	3.6	5.5	83	967	10	MOLECULE: RNA POLYMERASE-ASSOCIATED PROTEIN RAPA;
142	2ygu-F	3.6	3.8	64	118	6	MOLECULE: VENOM ALLERGEN 2;
143	7mca-F	3.6	6.3	71	164	11	MOLECULE: ORIGIN RECOGNITION COMPLEX SUBUNIT 1;
144	6f1t-f	3.6	17.8	95	929	7	MOLECULE: ARP1 ACTIN RELATED PROTEIN 1 HOMOLOG A;
145	2hh6-A	3.6	8.2	57	112	18	MOLECULE: BH3980 PROTEIN;
146	1ywm-A	3.6	11.5	82	180	10	MOLECULE: C PROTEIN ALPHA-ANTIGEN;
147	5yx4-A	3.6	9	59	232	3	MOLECULE: CHALCONE-FLAVONONE ISOMERASE FAMILY PROTEIN;
148	7eeb-C	3.6	12.7	69	278	10	MOLECULE: ENHANCED GREEN FLUORESCENT PROTEIN,CATION CHANNEL
149	1pn5-A	3.6	3.1	74	93	11	MOLECULE: NACHT-, LRR- AND PYD-CONTAINING PROTEIN 2;
150	6f1y-f	3.6	5.9	83	280	11	MOLECULE: CYTOPLASMIC DYNEIN 1 HEAVY CHAIN 1,DYNEIN HEAVY C
151	5w7g-A	3.6	5.6	56	131	7	MOLECULE: ORF140;
152	3jcm-I	3.5	16.6	85	416	7	MOLECULE: PRE-MRNA-SPLICING FACTOR 8;
153	5nx9-D	3.5	11.3	83	477	8	MOLECULE: ADENYLOSUCCINATE LYASE;
154	7jz2-A	3.5	11.7	74	481	4	MOLECULE: SUCCINATE DEHYDROGENASE FLAVOPROTEIN SUBUNIT;
155	6dg6-A	3.5	5	55	100	5	MOLECULE: NEOLEUKIN-2/15;
156	3hmj-A	3.5	9.4	65	1750	6	MOLECULE: FATTY ACID SYNTHASE SUBUNIT ALPHA;
157	2ogi-B	3.5	8.1	67	194	7	MOLECULE: HYPOTHETICAL PROTEIN SAG1661;
158	5aqd-M	3.5	3.8	55	184	2	MOLECULE: PHYCOERYTHRIN ALPHA SUBUNIT;
159	1sb0-A	3.5	2.7	47	87	4	MOLECULE: PROTEIN CBP;
160	7plh-A	3.5	5	48	260	10	MOLECULE: SHTNSC;
161	3u5z-A	3.5	18.5	84	186	6	MOLECULE: DNA POLYMERASE ACCESSORY PROTEIN 44;
162	5he9-E	3.5	2.5	42	56	7	MOLECULE: HELICASE LOADER;
163	4gzr-C	3.5	2.8	40	61	13	MOLECULE: ESAT-6-LIKE PROTEIN 6;
164	6vxm-A	3.5	9.3	52	277	15	MOLECULE: MECHANOSENSITIVE ION CHANNEL PROTEIN 1, MITOCHOND
165	6w6w-A	3.5	14	79	905	13	MOLECULE: CST COMPLEX SUBUNIT CTC1;
166	2dd4-H	3.5	5.9	67	156	6	MOLECULE: THIOCYANATE HYDROLASE ALPHA SUBUNIT;
167	3jc5-A	3.5	10.6	64	208	6	MOLECULE: DNA REPLICATION LICENSING FACTOR MCM2;
168	7jve-A	3.5	18.2	81	253	5	MOLECULE: DSBA FAMILY PROTEIN;
169	6lu0-A	3.5	17.7	92	937	12	MOLECULE: CAS12I2;
170	6myo-D	3.5	10.6	54	102	4	MOLECULE: SUCCINATE DEHYDROGENASE [UBIQUINONE] FLAVOPROTEIN
171	3if8-B	3.5	11.5	60	237	8	MOLECULE: PROTEIN ZWILCH HOMOLOG;
172	3w1h-A	3.5	9.7	64	452	6	MOLECULE: L-SERYL-TRNA(SEC) SELENIUM TRANSFERASE;

173	6nsj-A	3.5	6.5	64	182	8	MOLECULE: ACID-ACTIVATED UREA CHANNEL;
174	6vvo-E	3.5	9.4	81	346	6	MOLECULE: REPLICATION FACTOR C SUBUNIT 1;
175	7dpa-A	3.5	11.6	90	1642	12	MOLECULE: DEDICATOR OF CYTOKINESIS PROTEIN 5;
176	5ziq-A	3.5	2.7	63	155	5	MOLECULE: GLOBIN PROTEIN;
177	5ziy-A	3.5	5.2	62	195	5	MOLECULE: FLAGELLAR HOOK-ASSOCIATED PROTEIN 3;
178	7m2w-F	3.5	11.1	92	674	8	MOLECULE: TUBULIN GAMMA CHAIN;
179	3caz-B	3.5	8.9	73	210	7	MOLECULE: BAR PROTEIN;
180	5a9e-A	3.5	10.5	102	254	8	MOLECULE: DELTAMBD GAG PROTEIN;
181	6d03-E	3.5	4.4	75	466	4	MOLECULE: TRANSFERRIN RECEPTOR PROTEIN 1;
182	2px0-A	3.4	6.5	68	258	4	MOLECULE: FLAGELLAR BIOSYNTHESIS PROTEIN FLHF;
183	6efw-A	3.4	5.3	55	345	5	MOLECULE: ATP-DEPENDENT (S)-NAD(P)H-HYDRATE DEHYDRATASE;
184	3l9w-B	3.4	13.1	91	357	9	MOLECULE: GLUTATHIONE-REGULATED POTASSIUM-EFFLUX SYSTEM PRO
185	5n2j-B	3.4	23.1	76	1381	7	MOLECULE: UDP-GLUCOSE-GLYCOPROTEIN GLUCOSYLTRANSFERASE-
186	1n3k-A	3.4	4.4	94	130	12	MOLECULE: ASTROCYTIC PHOSPHOPROTEIN PEA-15;
187	6izw-B	3.4	8.6	49	145	8	MOLECULE: MUTUAL GLIDING-MOTILITY PROTEIN MGLA;
188	4lnb-A	3.4	6.3	60	339	5	MOLECULE: CAAX FARNESYLTRANSFERASE ALPHA SUBUNIT RAM2;
189	2nup-C	3.4	5.4	46	137	11	MOLECULE: PROTEIN TRANSPORT PROTEIN SEC23A;
190	2hxo-A	3.4	12.6	67	207	7	MOLECULE: PUTATIVE TETR-FAMILY TRANSCRIPTIONAL REGULATOR;
191	6yxp-B	3.4	3.6	65	272	9	MOLECULE: SWI/SNF COMPLEX SUBUNIT SMARCC1;
192	6n1l-A	3.4	3.3	53	141	8	MOLECULE: FIBRONECTIN-BINDING PROTEIN BBK32;
193	7dfe-A	3.4	7.1	97	142	5	MOLECULE: B6 PROTEIN;
194	7obq-u	3.4	5.4	64	441	5	MOLECULE: SRP RNA;
195	3p8c-F	3.4	6.8	60	156	7	MOLECULE: CYTOPLASMIC FMR1-INTERACTING PROTEIN 1;
196	4our-B	3.4	9.9	84	445	4	MOLECULE: PHYTOCHROME B;
197	4m0m-A	3.4	4.3	75	721	11	MOLECULE: PUTATIVE UNCHARACTERIZED PROTEIN;
198	7lxt-J	3.4	7.2	64	215	8	MOLECULE: 20S PROTEASOME ALPHA-1 SUBUNIT;
199	3oao-A	3.4	8.2	58	140	10	MOLECULE: UNCHARACTERIZED PROTEIN FROM DUF2059 FAMILY;
200	5wp4-A	3.4	10.7	87	487	9	MOLECULE: PHOSPHOETHANOLAMINE N-METHYLTRANSFERASE 1;
201	6l1q-A	3.4	10	66	267	6	MOLECULE: CBBQ PROTEIN;
202	6ueb-A	3.4	13.6	78	2099	6	MOLECULE: LARGE STRUCTURAL PROTEIN;
203	5dvi-B	3.4	10	49	396	4	MOLECULE: BINDING PROTEIN COMPONENT OF ABC SUGAR TRANSPORTE
204	2c5q-E	3.4	3.2	46	233	4	MOLECULE: RRAA-LIKE PROTEIN YER010C;
205	6kp3-B	3.4	3.3	64	112	6	MOLECULE: PROGRAMMED CELL DEATH 6-INTERACTING PROTEIN;
206	7s0r-A	3.4	4.4	56	85	9	MOLECULE: C PROTEIN BETA ANTIGEN;
207	4uzz-B	3.4	3.5	55	65	5	MOLECULE: INTRAFLAGELLAR TRANSPORT COMPLEX B PROTEIN 46

208	3fjv-A	3.4	7.5	67	193	10	MOLECULE: UNCHARACTERIZED NOVEL PROTEIN;
209	1lq7-A	3.4	3.8	52	67	6	MOLECULE: ALPHA3W;
210	7dvq-7	3.4	4.3	42	81	5	MOLECULE: PRE-MRNA-PROCESSING-SPLICING FACTOR 8;
211	6nes-A	3.4	7	57	431	0	MOLECULE: FAD-DEPENDENT MONOOXYGENASE TROPB;
212	5w7g-B	3.4	4.8	56	126	7	MOLECULE: ORF140;
213	6ks0-A	3.4	13.7	85	286	8	MOLECULE: ADIPONECTIN RECEPTOR PROTEIN 1;
214	6en3-A	3.4	12.5	82	934	7	MOLECULE: ENDO-BETA-N-ACETYLGLUCOSAMINIDASE F2,MULTIFUNCTIO
215	6zyd-A	3.4	9.5	67	323	10	MOLECULE: LOW CONDUCTANCE MECHANOSENSITIVE CHANNEL YNAI,LOW
216	4bt9-B	3.4	5.4	52	238	8	MOLECULE: PROLYL 4-HYDROXYLASE SUBUNIT ALPHA-1;
217	2nn4-A	3.4	7.5	47	62	6	MOLECULE: HYPOTHETICAL PROTEIN YQGQ;
218	5eqz-A	3.4	7	72	138	10	MOLECULE: REV PROTEIN;
219	4heo-B	3.4	3.3	48	60	6	MOLECULE: PHOSPHOPROTEIN;
220	3j5s-D	3.4	10.3	64	554	9	MOLECULE: 16S RIBOSOMAL RNA;
221	6lo8-D	3.4	9.7	53	119	8	MOLECULE: MITOCHONDRIAL IMPORT INNER MEMBRANE TRANSLOCASE S
222	3bbz-A	3.4	2.7	45	48	2	MOLECULE: P PROTEIN;
223	5gza-A	3.4	11.6	75	290	9	MOLECULE: PROTEIN O-MANNOSE KINASE;
224	5oxf-C	3.4	3.6	61	601	10	MOLECULE: GTP-BINDING PROTEIN;
225	6vbk-B	3.4	2.8	46	210	7	MOLECULE: LON211;
226	2do9-A	3.4	5.6	80	115	9	MOLECULE: NACHT-, LRR- AND PYD-CONTAINING PROTEIN 10;
227	3ezh-A	3.3	5.9	58	114	10	MOLECULE: NITRATE/NITRITE SENSOR PROTEIN NARX;
228	6jiv-A	3.3	10.9	83	560	8	MOLECULE: SSPE PROTEIN;
229	4m70-B	3.3	3.3	51	91	14	MOLECULE: RX PROTEIN;
230	5ulm-B	3.3	10.8	79	376	10	MOLECULE: MITOGEN-ACTIVATED PROTEIN KINASE KINASE KINASE 5;
231	6gw5-A	3.3	2.9	54	393	6	MOLECULE: PUTATIVE OUTER MEMBRANE PROTEIN;
232	3dkq-A	3.3	11.9	50	230	4	MOLECULE: PKHD-TYPE HYDROXYLASE SBAL_3634;
233	1sg9-A	3.3	10.1	64	276	5	MOLECULE: HEMK PROTEIN;
234	4kis-A	3.3	7.2	60	315	7	MOLECULE: PUTATIVE INTEGRASE [BACTERIOPHAGE A118];
235	6tv6-A	3.3	11.9	80	362	10	MOLECULE: PROTEIN-ARGININE KINASE;
236	2fsf-B	3.3	4.8	68	723	4	MOLECULE: PREPROTEIN TRANSLOCASE SECA SUBUNIT;
237	6qfo-A	3.3	3.8	83	1348	6	MOLECULE: PEGA DOMAIN-CONTAINING PROTEIN,PEGA DOMAIN-CONTAI
238	6rlb-A	3.3	11.2	95	969	8	MOLECULE: O6-ALKYLGUANINE-DNA ALKYLTRANSFERASE MUTANT,DYNC2
239	3viu-A	3.3	17.4	78	703	5	MOLECULE: PHOSPHORIBOSYLFORMYLGLYCINAMIDINE SYNTHASE 2;
240	3ugm-A	3.3	8.5	72	854	6	MOLECULE: TAL EFFECTOR AVRBS3/PTHA;
241	4dlq-A	3.3	7.7	83	353	5	MOLECULE: LATROPHILIN-1;
242	1szi-A	3.3	5.1	53	194	4	MOLECULE: MANNOSE-6-PHOSPHATE RECEPTOR BINDING PROTEIN 1;

243	6q7p-A	3.3	22.6	66	230	6	MOLECULE: OE1.2;
244	2e5t-A	3.3	4.2	39	46	15	MOLECULE: ATP SYNTHASE EPSILON CHAIN;
245	2jxn-A	3.3	9.8	63	116	10	MOLECULE: UNCHARACTERIZED PROTEIN YMR074C;
246	4ddg-A	3.3	6.9	62	399	13	MOLECULE: UBIQUITIN-CONJUGATING ENZYME E2 D2, UBIQUITIN THI
247	4ae4-B	3.3	2.8	42	115	7	MOLECULE: UBIQUITIN-ASSOCIATED PROTEIN 1;
248	2xa6-A	3.3	4.3	37	37	11	MOLECULE: KH DOMAIN-CONTAINING\,RNA-BINDING\,SIGNAL
249	6erp-A	3.3	4.5	84	1011	6	MOLECULE: TRANSCRIPTION FACTOR A, MITOCHONDRIAL;
250	2m6u-A	3.3	6.6	51	82	8	MOLECULE: CHOLINE BINDING PROTEIN A;
251	4qsz-A	3.3	11.6	85	686	11	MOLECULE: MALTOSE-BINDING PERIPLASMIC PROTEIN, JMJC DOMAIN-
252	6s7o-E	3.3	4.1	47	560	6	MOLECULE: DOLICHYL-DIPHOSPHOOLIGOSACCHARIDE--PROTEIN
253	3dye-A	3.3	5.3	88	302	10	MOLECULE: D7 PROTEIN;
254	6es4-A	3.3	2.3	42	207	7	MOLECULE: SYNCRIP, ISOFORM K;
255	1ej5-A	3.3	4.2	63	107	2	MOLECULE: WISKOTT-ALDRICH SYNDROME PROTEIN;
256	7d7c-F	3.3	6.7	79	137	9	MOLECULE: DNA-DIRECTED RNA POLYMERASE SUBUNIT ALPHA;
257	5fqf-A	3.3	7.5	72	583	11	MOLECULE: BETA-N-ACETYLGALACTOSAMINIDASE;
258	6djy-C	3.3	4.4	71	1041	7	MOLECULE: CLAMP PROTEIN;
259	7lt2-A	3.3	12.6	79	387	6	MOLECULE: MAB-21 DOMAIN-CONTAINING PROTEIN;
260	6m9a-C	3.3	4.9	54	157	6	MOLECULE: SIGNALING PROTEIN;
261	4wvm-B	3.3	11.4	88	616	10	MOLECULE: STONUSTOXIN SUBUNIT ALPHA;
262	5k47-A	3.3	10.8	52	484	6	MOLECULE: POLYCYSTIN-2;
263	4wbd-A	3.3	11.2	71	540	3	MOLECULE: BSHC;
264	4a4z-A	3.3	14.3	71	874	8	MOLECULE: ANTIVIRAL HELICASE SKI2;
265	6rjw-A	3.3	5.2	83	161	12	MOLECULE: LYSM DOMAIN PROTEIN;
266	5ckw-B	3.3	4.8	68	400	13	MOLECULE: LEGK4;
267	1nek-C	3.3	4.3	59	129	7	MOLECULE: SUCCINATE DEHYDROGENASE FLAVOPROTEIN SUBUNIT;
268	4ams-A	3.3	10.9	88	367	13	MOLECULE: MG662;
269	3bni-A	3.2	12.4	74	174	14	MOLECULE: PUTATIVE TETR-FAMILY TRANSCRIPTIONAL REGULATOR;
270	4afi-A	3.2	2.7	46	155	7	MOLECULE: AP-3 COMPLEX SUBUNIT DELTA-1, VESICLE-ASSOCIATED
271	5mpd-Q	3.2	12.3	77	434	5	MOLECULE: 26S PROTEASOME REGULATORY SUBUNIT RPN10;
272	4abn-B	3.2	6.8	55	426	9	MOLECULE: TETRATRICOPEPTIDE REPEAT PROTEIN 5;
273	2qq8-A	3.2	8.5	74	288	5	MOLECULE: TBC1 DOMAIN FAMILY MEMBER 14;
274	6e11-E	3.2	4	72	210	3	MOLECULE: UNKNOWN (CLAW);
275	7obq-y	3.2	15.6	71	454	8	MOLECULE: SRP RNA;
276	6ekk-B	3.2	11.4	74	381	8	MOLECULE: DENN DOMAIN-CONTAINING PROTEIN 1A;
277	2w4m-A	3.2	23.2	67	250	1	MOLECULE: N-ACYLNEURAMINATE-9-PHOSPHATASE;

278	3htk-C	3.2	13.1	52	254	8	MOLECULE: STRUCTURAL MAINTENANCE OF CHROMOSOMES PROTEIN 5;
279	5exr-B	3.2	4.4	57	434	18	MOLECULE: DNA PRIMASE SMALL SUBUNIT;
280	6swg-B	3.2	4.2	45	74	13	MOLECULE: PERIPHILIN-1;
281	6exn-a	3.2	5.8	54	171	9	MOLECULE: U2 SNRNA;
282	6e5o-Y	3.2	22.2	96	898	8	MOLECULE: CASX;
283	4oe8-C	3.2	3.8	57	87	11	MOLECULE: INTERLEUKIN-12 SUBUNIT BETA;
284	6ogd-E	3.2	12.5	78	1078	14	MOLECULE: TOXIN SUBUNIT YENA1;
285	2pv4-A	3.2	5.2	49	145	12	MOLECULE: UNCHARACTERIZED PROTEIN;
286	5wx8-A	3.2	3.8	61	165	10	MOLECULE: IMMEDIATE-EARLY PROTEIN 2;
287	6s3k-A	3.2	5.5	71	573	10	MOLECULE: APC FAMILY PERMEASE;
288	4zil-A	3.2	11.8	72	210	10	MOLECULE: DSBA OXIDOREDUCTASE;
289	6tdv-D	3.2	3.7	79	186	5	MOLECULE: ATPTB1;
290	6ikn-A	3.2	10.4	87	305	11	MOLECULE: GROWTH ARREST-SPECIFIC PROTEIN 7;
291	6nr8-6	3.2	4.6	46	102	11	MOLECULE: PREFOLDIN SUBUNIT 1;
292	6wpg-A	3.2	3.3	73	119	4	MOLECULE: REGULATORY PROTEIN NPR4;
293	2xvt-C	3.2	3.2	46	79	11	MOLECULE: RECEPTOR ACTIVITY-MODIFYING PROTEIN 2;
294	6o0i-A	3.2	4.6	37	98	5	MOLECULE: DESIGN CONSTRUCT XAA;
295	6dan-B	3.2	13.9	76	328	12	MOLECULE: PHDJ;
296	6vhf-A	3.2	4.4	41	140	10	MOLECULE: PHD-TYPE DOMAIN-CONTAINING PROTEIN;
297	6eu1-O	3.2	3.9	47	551	9	MOLECULE: DNA-DIRECTED RNA POLYMERASE III SUBUNIT RPC1;
298	4wvz-D	3.2	4.6	59	203	7	MOLECULE: THIOL DIOXYGENASE;
299	7jpp-E	3.2	12.9	71	406	6	MOLECULE: ORIGIN RECOGNITION COMPLEX SUBUNIT 1;
300	4etx-A	3.2	11	68	300	10	MOLECULE: PELD;
301	6wgm-A	3.2	8	58	305	3	MOLECULE: TRAP-TYPE C4-DICARBOXYLATE TRANSPORT SYSTEM, PERI
302	6uv7-B	3.2	11.4	70	154	10	MOLECULE: ALR1298 PROTEIN;
303	1jr3-D	3.2	10.5	75	338	8	MOLECULE: DNA POLYMERASE III SUBUNIT GAMMA;
304	6cfw-B	3.2	4.5	47	82	4	MOLECULE: MONOVALENT CATION/H+ ANTIPORTER SUBUNIT D;
305	2dwk-A	3.2	7.1	99	162	9	MOLECULE: PROTEIN RUFY3;
306	2xv9-A	3.2	3	69	134	13	MOLECULE: ABA-1A1 REPEAT UNIT;
307	1tiq-A	3.2	9	54	173	6	MOLECULE: PROTEASE SYNTHASE AND SPORULATION NEGATIVE REGULA
308	6l7o-G	3.2	8.2	48	192	10	MOLECULE: NAD(P)H-QUINONE OXIDOREDUCTASE SUBUNIT 1;
309	2b3t-A	3.2	10.8	67	277	9	MOLECULE: PROTEIN METHYLTRANSFERASE HEMK;
310	7k3h-A	3.2	4.9	57	104	11	MOLECULE: NETWORK HALLUCINATED PROTEIN 0217;
311	6r6b-F	3.2	9.1	59	245	10	MOLECULE: SURFACE PRESENTATION OF ANTIGENS PROTEIN SPAP;
312	5l4k-S	3.2	14.8	78	491	8	MOLECULE: 26S PROTEASOME NON-ATPASE REGULATORY SUBUNIT 4;

313	6cfw-A	3.2	2.8	43	165	0	MOLECULE: MONOVALENT CATION/H+ ANTIPORTER SUBUNIT D;
314	6lo8-C	3.2	3.9	50	108	12	MOLECULE: MITOCHONDRIAL IMPORT INNER MEMBRANE TRANSLOCASE S
315	6umm-A	3.2	6.3	61	285	8	MOLECULE: ESX-3 SECRETION SYSTEM PROTEIN ECCE3;
316	6jt0-B	3.2	12.1	88	576	7	MOLECULE: GUANYLATE CYCLASE SOLUBLE SUBUNIT ALPHA-1;
317	6h4j-A	3.2	10.9	55	488	9	MOLECULE: UBIQUITIN CARBOXYL-TERMINAL HYDROLASE 25;
318	5sy1-A	3.2	10.5	82	582	9	MOLECULE: CALMODULIN;
319	3vp5-A	3.2	12.8	61	185	11	MOLECULE: TRANSCRIPTIONAL REGULATOR;
320	6sty-A	3.2	9.7	71	198	11	MOLECULE: OLIGORIBONUCLEASE, MITOCHONDRIAL;
321	3ufe-A	3.2	4	49	109	14	MOLECULE: TRANSCRIPTIONAL ANTITERMINATOR (BGLG FAMILY);
322	3d5l-A	3.2	3.3	47	203	9	MOLECULE: REGULATORY PROTEIN RECX;
323	7t4x-B	3.2	5.1	58	450	9	MOLECULE: POTASSIUM CHANNEL AKT1;
324	1kw2-A	3.2	7	76	455	9	MOLECULE: VITAMIN D-BINDING PROTEIN;
325	6ulg-N	3.2	11.5	85	483	6	MOLECULE: FOLLICULIN;
326	2x0s-A	3.2	6.9	74	899	8	MOLECULE: PYRUVATE PHOSPHATE DIKINASE;
327	7aqq-N	3.2	7.5	84	488	10	MOLECULE: NADH-UBIQUINONE OXIDOREDUCTASE CHAIN 3;
328	7b8s-C	3.2	12.5	59	589	10	MOLECULE: MULTIDRUG EFFLUX PUMP SUBUNIT ACRB,MULTIDRUG EFFL
329	6swy-1	3.2	15.5	87	542	10	MOLECULE: VACUOLAR IMPORT AND DEGRADATION PROTEIN 28;
330	6hqv-A	3.2	5.2	127	1556	9	MOLECULE: PENTAFUNCTIONAL AROM POLYPEPTIDE;
331	4b3n-A	3.2	13.3	86	557	9	MOLECULE: MALTOSE-BINDING PERIPLASMIC PROTEIN, TRIPARTITE M
332	4p96-A	3.2	7.5	58	278	10	MOLECULE: FATTY ACID METABOLISM REGULATOR PROTEIN;
333	2ip6-A	3.2	2.6	50	87	6	MOLECULE: PAPB;
334	6dlu-P	3.2	8.5	72	747	8	MOLECULE: DYNAMIN-1;
335	1wgz-A	3.2	13.9	84	510	5	MOLECULE: CARBOXYPEPTIDASE 1;
336	7mez-B	3.2	17.1	89	547	7	MOLECULE: PHOSPHATIDYLINOSITOL 4,5-BISPHOSPHATE 3-KINASE CA
337	6ryp-A	3.1	4.3	47	164	11	MOLECULE: LIPOPROTEIN SIGNAL PEPTIDASE;
338	5y2g-A	3.1	10.4	74	581	7	MOLECULE: MALTOSE-BINDING PERIPLASMIC PROTEIN,PROTEIN B;
339	3u2r-A	3.1	4.4	46	135	15	MOLECULE: REGULATORY PROTEIN MARR;
340	6z5l-A	3.1	7.8	62	251	5	MOLECULE: MATRIX PROTEIN 1;
341	2j68-A	3.1	6	78	680	4	MOLECULE: BACTERIAL DYNAMIN-LIKE PROTEIN;
342	6vrb-C	3.1	10.4	57	229	18	MOLECULE: RNA (52-MER);
343	3dy5-A	3.1	11.6	57	1002	7	MOLECULE: ALLENE OXIDE SYNTHASE-LIPOXYGENASE PROTEIN;
344	4ihq-A	3.1	15.1	63	512	10	MOLECULE: FLAI ATPASE;
345	3pvz-A	3.1	13.9	61	372	7	MOLECULE: UDP-N-ACETYLGLUCOSAMINE 4,6-DEHYDRATASE;
346	3zx6-A	3.1	7.2	57	303	5	MOLECULE: HAMP, METHYL-ACCEPTING CHEMOTAXIS PROTEIN I;
347	3dxj-D	3.1	11.5	84	1504	6	MOLECULE: DNA-DIRECTED RNA POLYMERASE SUBUNIT ALPHA; CHAIN

348	1xqo-A	3.1	8.8	75	253	9	MOLECULE: 8-OXOGUANINE DNA GLYCOSYLASE;
349	6qd5-A	3.1	11.7	77	356	10	MOLECULE: UREA TRANSPORTER 1;
350	4nfu-B	3.1	13.8	60	524	8	MOLECULE: EDS1;
351	6gw6-B	3.1	3.1	61	149	8	MOLECULE: RES TOXIN;
352	3s1b-A	3.1	2.5	34	34	3	MOLECULE: VASCULAR ENDOTHELIAL GROWTH FACTOR A;
353	6sih-A	3.1	8.5	53	488	8	MOLECULE: FLAGELLAR HOOK-ASSOCIATED PROTEIN 2;
354	6uun-R	3.1	3	58	354	2	MOLECULE: GUANINE NUCLEOTIDE-BINDING PROTEIN G(S) SUBUNIT A
355	5jqk-A	3.1	4.3	66	631	8	MOLECULE: PEPTIDASE, PUTATIVE;
356	7nsb-a	3.1	13.1	65	568	11	MOLECULE: VACUOLAR IMPORT AND DEGRADATION PROTEIN 30;
357	7mrw-B	3.1	6.5	86	972	10	MOLECULE: CYTOADHERENCE LINKED ASEXUAL PROTEIN 3.1;
358	5wgr-A	3.1	17.4	94	664	4	MOLECULE: FLAVIN-DEPENDENT HALOGENASE;
359	5im8-A	3.1	2.8	41	60	5	MOLECULE: E3 UBIQUITIN-PROTEIN LIGASE MIDLINE-1;
360	4nqj-A	3.1	7.7	63	177	11	MOLECULE: E3 UBIQUITIN-PROTEIN LIGASE TRIM69;
361	6xss-A	3.1	5.7	59	260	12	MOLECULE: C4_NAT_HFUSE-7900;
362	5o5e-A	3.1	8.4	77	381	6	MOLECULE: UDP-N-ACETYLGLUCOSAMINE--DOLICHYL-PHOSPHATE N-
363	6mag-A	3.1	4	67	282	3	MOLECULE: BBVCI ENDONUCLEASE SUBUNIT 2;
364	2i5u-A	3.1	3.4	46	77	4	MOLECULE: DNAD DOMAIN PROTEIN;
365	6tmh-d	3.1	8.4	59	143	7	MOLECULE: INHIBITOR OF F1;
366	3o6x-A	3.1	7	83	639	7	MOLECULE: GLUTAMINE SYNTHETASE;
367	4kp3-D	3.1	5.5	55	82	7	MOLECULE: UNCONVENTIONAL MYOSIN-VA;
368	3hjl-A	3.1	3.8	57	321	7	MOLECULE: FLAGELLAR MOTOR SWITCH PROTEIN FLIG;
369	3zt9-A	3.1	11.9	62	192	5	MOLECULE: SERINE PHOSPHATASE;
370	6v3z-A	3.1	3.9	55	178	7	MOLECULE: SEN1395;
371	5ihf-B	3.1	2.7	53	109	4	MOLECULE: VIRG-LIKE PROTEIN;
372	6vm0-A	3.1	7.8	64	363	13	MOLECULE: GLYCINE RECEPTOR SUBUNIT ALPHAZ1;
373	4xxk-A	3.1	7.6	58	149	3	MOLECULE: PHYCOBILIPROTEIN APCE;
374	5xyv-C	3.1	6.5	45	52	0	MOLECULE: RHINO;
375	6z6o-D	3.1	7.5	60	542	7	MOLECULE: HISTONE DEACETYLASE HDA1;
376	4paf-A	3.1	9.8	54	308	9	MOLECULE: TRAP DICARBOXYLATE TRANSPORTER, DCTP SUBUNIT, PUT
377	7bl5-8	3.1	4.4	68	157	9	MOLECULE: 50S RIBOSOMAL PROTEIN L2;
378	3crl-B	3.1	6.2	83	383	6	MOLECULE: PYRUVATE DEHYDROGENASE [LIPOAMIDE] KINASE ISOZYME
379	5img-A	3.1	12.6	95	467	8	MOLECULE: DIPEPTIDASE;
380	3r2x-C	3.1	2.6	46	82	7	MOLECULE: HEMAGGLUTININ;
381	4neo-A	3.1	2.6	51	83	8	MOLECULE: PEPTIDE SYNTHETASE NRPS TYPE II-PCP;
382	7js4-A	3.1	16.6	91	953	10	MOLECULE: F5/8 TYPE C DOMAIN PROTEIN;

383	4wat-A	3.1	5.1	71	332	6	MOLECULE: PFRH5;
384	5nwf-A	3.1	3	38	203	5	MOLECULE: FIC FAMILY PROTEIN;
385	6veo-A	3.1	4.8	57	136	9	MOLECULE: ATPASE FAMILY AAA DOMAIN-CONTAINING PROTEIN 2B;
386	3jbz-A	3.1	14.4	82	960	4	MOLECULE: SERINE/THREONINE-PROTEIN KINASE MTOR;
387	6z5s-W	3.1	5.4	44	94	0	MOLECULE: LIGHT HARVESTING COMPLEX 1 PROTEIN W;
388	1ufi-A	3.1	5.4	40	48	15	MOLECULE: MAJOR CENTROMERE AUTOANTIGEN B;
389	6hvn-A	3.1	2.6	42	175	7	MOLECULE: DIADENYLATE CYCLASE;
390	2lse-A	3.1	3.3	60	101	10	MOLECULE: FOUR HELIX BUNDLE PROTEIN;
391	4wd9-A	3.1	11.9	67	965	9	MOLECULE: NISIN BIOSYNTHESIS PROTEIN NISB;
392	4fcg-A	3.1	15.9	70	296	11	MOLECULE: UNCHARACTERIZED PROTEIN;
393	2qw6-C	3.1	3	55	83	13	MOLECULE: AAA ATPASE, CENTRAL REGION;
394	5fia-B	3.1	12.2	102	397	4	MOLECULE: LPIR1;
395	6c48-D	3.1	5.5	53	87	13	MOLECULE: PROTEIN LIN-9 HOMOLOG;
396	5cwf-A	3.1	7	66	177	12	MOLECULE: DESIGNED HELICAL REPEAT PROTEIN;
397	6prk-A	3.1	10.8	62	115	11	MOLECULE: RICF;
398	4cc9-B	3.1	4.9	65	98	2	MOLECULE: PROTEIN VPRBP;
399	6yrf-A	3.1	15.9	84	777	7	MOLECULE: VEGETATIVE INSECTICIDAL PROTEIN;
400	1kmi-Z	3.1	4.8	45	177	11	MOLECULE: CHEMOTAXIS PROTEIN CHEY;
401	6jcx-F	3.1	5.4	67	186	6	MOLECULE: DNA-DIRECTED RNA POLYMERASE SUBUNIT ALPHA;
402	7a5p-S	3.1	3.1	41	46	12	MOLECULE: U2 SNRNA;
403	3gnl-A	3.1	11.6	70	234	6	MOLECULE: UNCHARACTERIZED PROTEIN, DUF633, LMOF2365_1472;
404	6m0r-M	3.1	5.9	57	71	11	MOLECULE: V-TYPE PROTON ATPASE SUBUNIT C';
405	6sum-A	3.1	4.3	58	335	7	MOLECULE: AMICOUMACIN KINASE;
406	3wdm-A	3.1	8.1	61	259	7	MOLECULE: 4-PHOSPHOPANTOATE--BETA-ALANINE LIGASE;
407	7et3-i	3.1	2.9	47	63	17	MOLECULE: TRIPLEX CAPSID PROTEIN 2;
408	5xef-A	3.1	5	43	123	7	MOLECULE: FLAGELLAR PROTEIN FLIS;
409	1yhd-A	3.1	4.2	55	92	11	MOLECULE: UPF0269 PROTEIN YGGX;
410	5iwz-A	3.1	5.7	78	372	9	MOLECULE: SYNAPTONEMAL COMPLEX PROTEIN 2;
411	6e3y-E	3.1	4.2	46	115	7	MOLECULE: CALCITONIN GENE-RELATED PEPTIDE 1;
412	6vm6-B	3.1	6.2	60	440	5	MOLECULE: SAVED DOMAIN-CONTAINING PROTEIN;
413	3rgu-A	3.1	3.7	57	88	5	MOLECULE: FIMBRIAE-ASSOCIATED PROTEIN FAP1;
414	7bwk-E	3	3.3	60	116	8	MOLECULE: ICMO (DOTL);
415	3glf-G	3	16.9	69	378	6	MOLECULE: DNA POLYMERASE III SUBUNIT DELTA;
416	4gae-A	3	13	75	416	5	MOLECULE: 1-DEOXY-D-XYLULOSE 5-PHOSPHATE REDUCTOISOMERASE,
417	2x2v-A	3	3.2	49	69	8	MOLECULE: ATP SYNTHASE SUBUNIT C;

418	7jgr-D	3	11.3	73	441	5	MOLECULE: ORIGIN RECOGNITION COMPLEX SUBUNIT 2;
419	6wmk-A	3	2.7	45	65	9	MOLECULE: BETA SHEET HETERODIMER LHD29 - CHAIN A;
420	3ilk-A	3	10.7	48	239	8	MOLECULE: UNCHARACTERIZED TRNA/RRNA METHYLTRANSFERASE HI038
421	6maf-A	3	4.8	70	259	11	MOLECULE: BBVCI ENDONUCLEASE SUBUNIT 1;
422	6vzd-E	3	5.9	51	85	6	MOLECULE: PULMONARY SURFACTANT-ASSOCIATED PROTEIN B;
423	5utg-A	3	5.9	59	134	7	MOLECULE: EGG-LYSIN;
424	4okv-F	3	4.3	45	66	9	MOLECULE: HEAVY CHAIN OF 8H7 MAB;
425	5h79-D	3	9.1	53	142	4	MOLECULE: IMMUNOGLOBULIN G-BINDING PROTEIN A;
426	5x41-F	3	14.5	77	244	10	MOLECULE: COBALT ABC TRANSPORTER ATP-BINDING PROTEIN;
427	1yns-A	3	7.3	96	254	7	MOLECULE: E-1 ENZYME;
428	3kdq-C	3	5.1	54	153	7	MOLECULE: UNCHARACTERIZED CONSERVED PROTEIN;
429	5h75-D	3	3	45	225	9	MOLECULE: MERSACIDIN DECARBOXYLASE,IMMUNOGLOBULIN G-BINDING
430	5fb1-A	3	3.6	72	174	11	MOLECULE: NUCLEAR AUTOANTIGEN SP-100;
431	5wp0-B	3	14.7	63	274	6	MOLECULE: NH(3)-DEPENDENT NAD(+) SYNTHETASE;
432	6w38-A	3	4.8	50	331	12	MOLECULE: TERMINAL NUCLEOTIDYLTRANSFERASE 5C;
433	7f1t-A	3	5.1	71	423	7	MOLECULE: C-C MOTIF CHEMOKINE 3,C-C CHEMOKINE RECEPTOR TYPE
434	2kyz-A	3	2.8	42	67	10	MOLECULE: HEAVY METAL BINDING PROTEIN;
435	6upn-A	3	9.7	54	245	17	MOLECULE: ENDOPHILIN-B1;
436	7t62-A	3	9.1	91	561	7	MOLECULE: GLYPICAN-2;
437	5c0x-G	3	10	67	237	7	MOLECULE: EXOSOME COMPLEX COMPONENT RRP45;
438	4ifd-H	3	12.3	61	295	2	MOLECULE: EXOSOME COMPLEX COMPONENT RRP45;
439	4bbp-A	3	7.6	72	266	15	MOLECULE: ZINC ABC TRANSPORTER, PERIPLASMIC ZINC-BINDING PR
440	6mur-B	3	7.9	56	162	11	MOLECULE: UNCHARACTERIZED PROTEIN;
441	7eu3-2	3	5.8	52	129	6	MOLECULE: NAD(P)H-QUINONE OXIDOREDUCTASE SUBUNIT 1, CHLOROP
442	4hqo-B	3	11.1	63	260	10	MOLECULE: SPOROZOITE SURFACE PROTEIN 2;
443	2etd-A	3	6.6	72	141	4	MOLECULE: LEMA PROTEIN;
444	4u8u-G	3	4.6	62	151	2	MOLECULE: GLOBIN A CHAIN;
445	3wx4-A	3	8.7	63	98	14	MOLECULE: ANTI-RESTRICTION ENDONUCLEASE;
446	3hw2-A	3	7.1	64	308	3	MOLECULE: PROTEIN SIFA;
447	6yj4-L	3	8.3	82	655	7	MOLECULE: NADH-UBIQUINONE OXIDOREDUCTASE CHAIN 3;
448	2dgj-A	3	7.7	71	246	7	MOLECULE: HYPOTHETICAL PROTEIN EBHA;
449	3iym-A	3	8.6	73	396	10	MOLECULE: CAPSID PROTEIN;
450	3odn-A	3	9.3	94	366	1	MOLECULE: DALLY-LIKE PROTEIN;
451	2d5w-A	3	9.7	75	602	5	MOLECULE: PEPTIDE ABC TRANSPORTER, PEPTIDE-BINDING PROTEIN;
452	1nu7-D	3	6.2	77	282	6	MOLECULE: THROMBIN LIGHT CHAIN;

453	6urt-A	3	10.1	68	331	4	MOLECULE: LOW CONDUCTANCE MECHANOSENSITIVE CHANNEL YNAI;
454	4x4w-A	3	10.1	63	398	10	MOLECULE: CCA TRNA NUCLEOTIDYLTRANSFERASE 1, MITOCHONDRIAL;
455	3oov-A	3	5.2	41	164	12	MOLECULE: METHYL-ACCEPTING CHEMOTAXIS PROTEIN, PUTATIVE;
456	5fno-A	3	9.8	65	568	9	MOLECULE: MANGANESE LIPOXYGENASE;
457	6xqk-A	3	2.1	37	47	11	MOLECULE: CAMP-DEPENDENT PROTEIN KINASE REGULATORY SUBUNIT;
458	7kgm-A	3	4.9	82	512	6	MOLECULE: PUTATIVE EXPORTED PROTEIN;
459	6dgc-A	3	10.5	42	147	0	MOLECULE: ISC1926 TNPA C-TERMINAL CATALYTIC DOMAIN;
460	6gse-A	3	10.8	73	159	8	MOLECULE: ACTIVITY-REGULATED CYTOSKELETON-ASSOCIATED PROTEI
461	7nza-A	3	7.7	64	141	5	MOLECULE: ODORANT BINDING PROTEIN FROM VARROA DESTRUCTOR, F
462	6xtt-B	3	11.1	61	116	7	MOLECULE: NTTA;
463	5n8k-C	3	8.2	49	80	4	MOLECULE: GALACTOCEREBROSIDASE;
464	3bjd-C	3	6.8	78	324	10	MOLECULE: PUTATIVE 3-OXOACYL-(ACYL-CARRIER-PROTEIN) SYNTHAS
465	5lfj-B	3	5.1	60	113	8	MOLECULE: BACTERIAL PROTEASOME ACTIVATOR;
466	6oiw-A	3	4.9	100	504	7	MOLECULE: DEOXYGUANOSINETRIPHOSPHATE TRIPHOSPHOHYDROLASE;
467	6br8-A	3	13.8	79	249	5	MOLECULE: PROTEIN A6 HOMOLOG;
468	2c2l-A	3	10.1	68	281	6	MOLECULE: CARBOXY TERMINUS OF HSP70-INTERACTING PROTEIN;
469	6z11-H	3	17	56	193	2	MOLECULE: DNA-DIRECTED RNA POLYMERASE SUBUNIT ALPHA;
470	6c6r-A	3	8.6	68	451	6	MOLECULE: SQUALENE MONOOXYGENASE;
471	2es4-E	3	2.6	46	278	7	MOLECULE: LIPASE;
472	3jrt-A	3	5.9	65	166	9	MOLECULE: INTEGRON CASSETTE PROTEIN VPC_CASS2;
473	6cgh-A	3	3.7	54	89	9	MOLECULE: DNAJ HOMOLOG SUBFAMILY C MEMBER 2;
474	6ysg-A	3	17.8	84	1264	11	MOLECULE: MG-CHELATASE SUBUNIT CHLH;
475	1jj2-O	3	7.2	65	143	9	MOLECULE: 23S RRNA;
476	6hyd-A	3	13.8	86	1574	10	MOLECULE: MIDASIN,MIDASIN,MIDASIN;
477	7jsj-A	3	10.6	77	468	9	MOLECULE: SOLUTE CARRIER FAMILY 13 MEMBER 5;
478	6ah0-w	3	5.4	53	443	4	MOLECULE: U5SNRNA;
479	6fwr-A	3	9.6	82	699	4	MOLECULE: ATP-DEPENDENT DNA HELICASE DING;
480	4wpe-A	3	6.1	79	275	13	MOLECULE: CYTOKINESIS PROTEIN 2;
481	6em5-R	3	3.6	45	120	18	MOLECULE: 5.8S RIBOSOMAL RNA;
482	5vvr-B	3	8	80	1207	14	MOLECULE: DNA-DIRECTED RNA POLYMERASE II SUBUNIT RPB1;
483	2e62-A	3	2.8	43	61	9	MOLECULE: PROTEIN AT5G25060;
484	4z7x-A	3	7.7	67	211	4	MOLECULE: MDBA;
485	2khv-A	3	3.7	41	106	0	MOLECULE: PHAGE INTEGRASE;
486	6xi6-A	3	5.7	53	269	8	MOLECULE: HELICAL FUSION DESIGN;
487	3bpt-A	3	17.6	80	362	5	MOLECULE: 3-HYDROXYISOBUTYRYL-COA HYDROLASE;

488	3owg-A	3	16.6	102	457	5	MOLECULE: POLY(A) POLYMERASE CATALYTIC SUBUNIT;
489	6w17-F	3	14.1	51	168	8	MOLECULE: ACTIN-RELATED PROTEIN 3;
490	5ztc-A	3	11.8	71	197	14	MOLECULE: LMO2088 PROTEIN;
491	6vjr-A	3	5	61	502	11	MOLECULE: VINYLACETYL-COA DELTA-ISOMERASE;
492	5j0l-A	3	3.9	65	130	11	MOLECULE: DESIGNED PROTEIN 3L6HC2_2;
493	4zs9-B	3	3.2	50	378	8	MOLECULE: SUGAR BINDING PROTEIN OF ABC TRANSPORTER SYSTEM;
494	6tdz-C	3	11.9	76	530	5	MOLECULE: OLIGOMYCIN SENSITIVITY CONFERRING PROTEIN (OSCP);
495	6z5u-F	3	9.9	53	193	9	MOLECULE: ABC TRANSPORTER PERMEASE;
496	7b93-L	3	9.6	92	607	10	MOLECULE: NADH-UBIQUINONE OXIDOREDUCTASE CHAIN 3;
497	5i04-A	3	13.4	81	668	6	MOLECULE: MALTOSE-BINDING PERIPLASMIC PROTEIN,ENDOGLIN;
498	6nd4-J	3	8.4	60	493	3	MOLECULE: ETS RRNA;
499	7a6h-O	3	10.3	54	512	7	MOLECULE: DNA-DIRECTED RNA POLYMERASE III SUBUNIT RPC1;
500	6z16-d	3	4.9	70	489	7	MOLECULE: MULTISUBUNIT NA+/H+ ANTIPORTER, A SUBUNIT;
501	4x2c-A	3	5.6	54	207	9	MOLECULE: FIC FAMILY PROTEIN PUTATIVE FILAMENTATION INDUCED
502	2w4s-A	3	3	56	86	13	MOLECULE: ANKYRIN-REPEAT PROTEIN;
503	4dn7-A	2.9	4.1	82	406	4	MOLECULE: ABC TRANSPORTER, ATP-BINDING PROTEIN;
504	7jrl-A	2.9	10.2	94	457	11	MOLECULE: F5/8 TYPE C DOMAIN PROTEIN;
505	1fx2-A	2.9	3.4	59	235	10	MOLECULE: RECEPTOR-TYPE ADENYLATE CYCLASE GRESAG 4.1;
506	3g5b-A	2.9	2.8	77	383	6	MOLECULE: NETRIN RECEPTOR UNC5B;
507	6rie-A	2.9	19.4	133	1285	9	MOLECULE: DNA-DEPENDENT RNA POLYMERASE SUBUNIT RPO147;
508	5anz-A	2.9	8.6	81	375	5	MOLECULE: SOLUBLE LYTIC TRANSGLYCOSYLASE B3;
509	3mkw-B	2.9	3.3	47	115	6	MOLECULE: DNA (5'-
510	7o8c-A	2.9	3.4	49	582	6	MOLECULE: SURFACE GLYCAN-BINDING PROTEIN BO2743;
511	6ks5-B	2.9	6.5	85	313	12	MOLECULE: TYPE IV SECRETION PROTEIN DOT;
512	5byh-D	2.9	11.1	86	1372	2	MOLECULE: DNA-DIRECTED RNA POLYMERASE SUBUNIT ALPHA;
513	5z7b-B	2.9	9.8	88	197	5	MOLECULE: PADR FAMILY TRANSCRIPTIONAL REGULATOR;
514	1k32-A	2.9	9.9	96	1023	3	MOLECULE: TRICORN PROTEASE;
515	6snr-A	2.9	9.5	58	402	10	MOLECULE: LIPID II:GLYCINE GLYCYLTRANSFERASE;
516	6ird-B	2.9	3.8	57	254	5	MOLECULE: 1-PHOSPHATIDYLINOSITOL 4,5-BISPHOSPHATE PHOSPHODI
517	4g33-A	2.9	14.2	86	661	9	MOLECULE: 15S-LIPOXYGENASE;
518	2qe9-A	2.9	8.1	68	165	4	MOLECULE: UNCHARACTERIZED PROTEIN YIZA;
519	6uva-E	2.9	15	51	111	4	MOLECULE: GUANINE NUCLEOTIDE-BINDING PROTEIN G(S) SUBUNIT A
520	6of3-A	2.9	5.1	75	187	7	MOLECULE: RIBONUCLEASE;
521	5jw9-B	2.9	8.4	47	115	4	MOLECULE: AF4/FMR2 FAMILY MEMBER 4;
522	4m70-R	2.9	2.6	41	56	0	MOLECULE: RX PROTEIN;

523	1hlv-A	2.9	13	57	131	11	MOLECULE: CENP-B BOX DNA;
524	1ysm-A	2.9	4.7	42	55	10	MOLECULE: CALCYCLIN-BINDING PROTEIN;
525	2pyw-A	2.9	10.6	77	417	6	MOLECULE: UNCHARACTERIZED PROTEIN;
526	6ff1-A	2.9	2.5	40	69	18	MOLECULE: CSOZ;
527	6v85-F	2.9	3.1	44	47	5	MOLECULE: RNA-DIRECTED RNA POLYMERASE L;
528	6vls-D	2.9	9.2	74	963	7	MOLECULE: MALTOSE/MALTODEXTRIN-BINDING PERIPLASMIC PROTEIN,
529	6z16-f	2.9	4.7	45	88	11	MOLECULE: MULTISUBUNIT NA+/H+ ANTIPORTER, A SUBUNIT;
530	5v8f-6	2.9	6.7	89	692	7	MOLECULE: DNA REPLICATION LICENSING FACTOR MCM2;
531	4ykn-A	2.9	12.9	65	1204	9	MOLECULE: PHOSPHATIDYLINOSITOL 3-KINASE REGULATORY SUBUNIT
532	7m4p-A	2.9	10.9	101	1049	6	MOLECULE: EFFLUX PUMP MEMBRANE TRANSPORTER;
533	2yia-A	2.9	12.7	73	790	7	MOLECULE: RNA-DIRECTED RNA POLYMERASE;
534	3ksy-A	2.9	4.9	96	1007	9	MOLECULE: SON OF SEVENLESS HOMOLOG 1;
535	1yre-B	2.9	9.3	45	187	0	MOLECULE: HYPOTHETICAL PROTEIN PA3270;
536	7ns4-i	2.9	4.4	53	358	6	MOLECULE: E3 UBIQUITIN-PROTEIN LIGASE RMD5;
537	3hj1-B	2.9	8.5	77	387	6	MOLECULE: MINOR EDITOSOME-ASSOCIATED TUTASE;
538	6bx3-F	2.9	5.6	54	215	11	MOLECULE: HISTONE-LYSINE N-METHYLTRANSFERASE, H3 LYSINE-4 S
539	4n4u-A	2.9	10.3	51	304	10	MOLECULE: PUTATIVE ABC TRANSPORTER PERIPLASMIC SOLUTE-BINDI
540	7ebe-G	2.9	21	88	535	8	MOLECULE: ISOCITRATE LYASE;
541	4jvy-A	2.9	5.6	66	191	11	MOLECULE: FEMALE GERMLINE-SPECIFIC TUMOR SUPPRESSOR GLD-1;
542	4xc6-A	2.9	12.2	98	1067	6	MOLECULE: ISOBUTYRYL-COA MUTASE FUSED;
543	6cfw-G	2.9	4.6	49	113	6	MOLECULE: MONOVALENT CATION/H+ ANTIPORTER SUBUNIT D;
544	3g06-A	2.9	5.9	77	601	6	MOLECULE: SSPH2 (LEUCINE-RICH REPEAT PROTEIN);
545	6qm7-H	2.9	9.2	72	229	3	MOLECULE: PROTEASOME ALPHA1 CHAIN;
546	5ue8-A	2.9	11.3	90	847	8	MOLECULE: PROTEIN UNC-13 HOMOLOG A;
547	7eeb-K	2.9	4.3	52	143	10	MOLECULE: ENHANCED GREEN FLUORESCENT PROTEIN,CATION CHANNEL
548	6ln2-A	2.9	5.3	73	437	3	MOLECULE: GLUCAGON-LIKE PEPTIDE 1 RECEPTOR,RUBREDOXIN,GLUCA
549	6ezn-B	2.9	8.9	46	110	9	MOLECULE: DOLICHYL-DIPHOSPHOOLIGOSACCHARIDE--PROTEIN
550	5y08-A	2.9	4.7	60	205	10	MOLECULE: 4-HYDROXY-2,2'-BIPYRROLE-5-METHANOL SYNTHASE PIGH
551	6t1z-A	2.9	6	65	393	6	MOLECULE: LMRP INTEGRAL MEMBRANE PROTEIN;
552	3f44-A	2.9	14.7	62	210	13	MOLECULE: PUTATIVE MONOOXYGENASE;
553	3mp8-A	2.9	14.3	74	513	15	MOLECULE: MALTOSE-BINDING PERIPLASMIC PROTEIN,LINKER,SAGA-A
554	7sr1-A	2.9	3	42	123	7	MOLECULE: SORTING NEXIN-25;
555	2rno-A	2.9	3.5	71	110	7	MOLECULE: PUTATIVE DNA-BINDING PROTEIN;
556	4eyy-R	2.9	4.8	45	59	7	MOLECULE: ICMR;
557	3t38-A	2.9	8.4	59	199	7	MOLECULE: ARSENATE REDUCTASE;

558	6v3f-A	2.9	8	96	1234	3	MOLECULE: NPC1-LIKE INTRACELLULAR CHOLESTEROL TRANSPORTER 1
559	2oxl-A	2.9	9.4	52	62	15	MOLECULE: HYPOTHETICAL PROTEIN YMGB;
560	6soy-A	2.9	10.1	77	323	8	MOLECULE: ESAG6, SUBUNIT OF HETERODIMERIC TRANSFERRIN RECEP
561	2qpx-A	2.9	4.3	47	376	9	MOLECULE: PREDICTED METAL-DEPENDENT HYDROLASE OF THE TIM-BA
562	5ukv-A	2.9	4.2	49	74	12	MOLECULE: ATP-BINDING PROTEIN;
563	2mpn-A	2.9	4.6	45	68	4	MOLECULE: INNER MEMBRANE PROTEIN YGAP;
564	6idp-A	2.9	8.5	77	438	3	MOLECULE: MATE FAMILY EFFLUX TRANSPORTER;
565	4oo8-D	2.9	11.4	87	1163	7	MOLECULE: CRISPR-ASSOCIATED ENDONUCLEASE CAS9/CSN1;
566	3bqy-A	2.9	10.8	69	205	6	MOLECULE: PUTATIVE TETR FAMILY TRANSCRIPTIONAL REGULATOR;
567	1pjq-B	2.9	12.4	69	456	4	MOLECULE: SIROHEME SYNTHASE;
568	5m41-A	2.9	4.6	115	712	11	MOLECULE: NIGRITOXINE;
569	6lum-G	2.9	4	58	123	7	MOLECULE: SUCCINATE DEHYDROGENASE SUBUNIT C;
570	4fzo-A	2.9	5	54	82	9	MOLECULE: URANYL BINDING PROTEIN;
571	7mi6-A	2.9	13.2	97	2419	5	MOLECULE: FUSION PROTEIN OF DYNEIN AND ENDOLYSIN;
572	2ols-A	2.9	15.1	59	725	7	MOLECULE: PHOSPHOENOLPYRUVATE SYNTHASE;
573	6wtw-A	2.9	9.7	66	489	9	MOLECULE: DASS FAMILY SODIUM-COUPLED ANION SYMPORTER;
574	5udb-7	2.9	16.3	94	726	5	MOLECULE: DNA REPLICATION LICENSING FACTOR MCM2;
575	5tj4-H	2.9	11.7	87	537	10	MOLECULE: SUGAR ABC TRANSPORTER SUBSTRATE-BINDING PROTEIN,G
576	4lws-A	2.9	7	58	100	7	MOLECULE: UNCHARACTERIZED PROTEIN;
577	6khn-A	2.9	11.2	71	490	8	MOLECULE: TRYPTOPHAN DECARBOXYLASE 1;
578	5vyl-A	2.9	10.8	80	502	1	MOLECULE: INNER TEGUMENT PROTEIN;
579	1iru-G	2.9	5	65	245	8	MOLECULE: 20S PROTEASOME;
580	4d7x-A	2.9	3.6	48	86	17	MOLECULE: MEDIATOR OF RNA POLYMERASE II TRANSCRIPTION SUBUN
581	5jm8-A	2.9	9.6	77	556	4	MOLECULE: AEROBACTIN SYNTHASE IUCA;
582	7aoz-I	2.9	3.9	71	464	11	MOLECULE: DNA-DIRECTED RNA POLYMERASE 147 KDA POLYPEPTIDE;
583	5c9e-A	2.9	10.5	63	263	13	MOLECULE: SEPL;
584	3t58-B	2.9	5.8	67	504	3	MOLECULE: SULFHYDRYL OXIDASE 1;
585	4cti-D	2.9	8.2	57	222	5	MOLECULE: OSMOLARITY SENSOR PROTEIN ENVZ, AF1503;
586	4z39-A	2.9	3.1	58	121	7	MOLECULE: ODORANT-BINDING PROTEIN;
587	1kyq-B	2.9	9.8	73	273	8	MOLECULE: SIROHEME BIOSYNTHESIS PROTEIN MET8;
588	5j10-A	2.9	4.4	39	70	23	MOLECULE: PEPTIDE DESIGN 2L4HC2_24;
589	5a1q-A	2.9	3.2	57	69	11	MOLECULE: AF1502;
590	5i5k-B	2.9	14.7	95	1634	9	MOLECULE: COMPLEMENT C5;
591	5lbm-C	2.9	2.7	41	90	5	MOLECULE: TRANSCRIPTIONAL REPRESSOR FRMR;
592	7sh3-A	2.9	3	49	63	12	MOLECULE: SYNTHETIC VIRB8 MINIPROTEIN BINDER;

593	5xsv-A	2.9	4.9	82	486	7	MOLECULE: CHITINASE;
594	7but-A	2.9	5.9	77	142	6	MOLECULE: ACINIFORM SPIDROIN;
595	5bq9-B	2.9	9.6	67	437	9	MOLECULE: UNCHARACTERIZED PROTEIN;
596	6yvu-B	2.9	8.4	75	1191	5	MOLECULE: STRUCTURAL MAINTENANCE OF CHROMOSOMES PROTEIN 2,S
597	1r4g-A	2.9	3	46	53	9	MOLECULE: RNA POLYMERASE ALPHA SUBUNIT;
598	7drt-B	2.9	4.3	65	496	6	MOLECULE: PROTEIN WNT-3A;
599	4f4h-A	2.9	17	87	540	10	MOLECULE: GLUTAMINE DEPENDENT NAD+ SYNTHETASE;
600	4bk0-B	2.9	4.1	38	97	8	MOLECULE: ATP-DEPENDENT DNA HELICASE Q5;
601	4gcz-B	2.9	8.4	60	378	2	MOLECULE: BLUE-LIGHT PHOTORECEPTOR, SENSOR PROTEIN FIXL;
602	4uxv-A	2.9	5.8	72	522	7	MOLECULE: SEPTATION RING FORMATION REGULATOR EZRA;
603	4zel-A	2.9	9.7	60	550	12	MOLECULE: DOPAMINE BETA-HYDROXYLASE;
604	6hwj-A	2.9	9.9	69	428	10	MOLECULE: MALTOKINASE;
605	5ogs-A	2.9	10	77	403	4	MOLECULE: WD REPEAT AND HMG-BOX DNA-BINDING PROTEIN 1;
606	2lqx-A	2.9	3.2	38	41	5	MOLECULE: TRYPSIN INHIBITOR BWI-2C;
607	6elu-A	2.9	4.3	62	193	8	MOLECULE: SERUM RESISTANCE ASSOCIATED; VSG PROTEIN;
608	5jje-B	2.9	3.9	44	63	0	MOLECULE: SENSORY RHODOPSIN-2;
609	7lt1-A	2.9	3.7	67	403	3	MOLECULE: PROTEIN MB21D2;
610	6t58-A	2.9	9.7	67	524	7	MOLECULE: CELLULAR TUMOR ANTIGEN P53,PROTEIN S100-A4,PROTEI
611	5t58-A	2.9	4.4	65	233	5	MOLECULE: KLLA0F02343P;
612	3wfw-A	2.9	8.2	61	138	10	MOLECULE: HEMOGLOBIN-LIKE FLAVOPROTEIN FUSED TO ROADBLOCK/L
613	2zy4-F	2.9	16.3	71	535	4	MOLECULE: L-ASPARTATE BETA-DECARBOXYLASE;
614	6gho-B	2.8	13.4	83	274	5	MOLECULE: REGULATORY PROTEIN SPX;
615	1ysy-A	2.8	3.4	52	85	4	MOLECULE: REPLICASE POLYPROTEIN 1AB (PP1AB) (ORF1AB);
616	2win-M	2.8	6.8	64	84	8	MOLECULE: COMPLEMENT C3 BETA CHAIN;
617	3s8r-B	2.8	10.5	71	686	4	MOLECULE: GLUTARYL-7-AMINOCEPHALOSPORANIC-ACID ACYLASE;
618	6fhs-C	2.8	8.3	67	459	6	MOLECULE: RUVB-LIKE HELICASE;
619	3omd-A	2.8	4	67	145	9	MOLECULE: UNCHARACTERIZED PROTEIN;
620	3hai-A	2.8	8.6	82	294	1	MOLECULE: HUMAN PACSIN1 F-BAR;
621	7kpx-C	2.8	15.6	68	1352	1	MOLECULE: MEIOTIC MRNA STABILITY PROTEIN KINASE SSN3;
622	6uz8-A	2.8	12.3	65	734	3	MOLECULE: SHORT TRANSIENT RECEPTOR POTENTIAL CHANNEL 6;
623	7abw-B	2.8	8.6	59	365	3	MOLECULE: PEPSY DOMAIN-CONTAINING PROTEIN;
624	3ats-A	2.8	8	76	352	8	MOLECULE: PUTATIVE UNCHARACTERIZED PROTEIN;
625	6c0f-D	2.8	10.6	82	190	10	MOLECULE: SACCHAROMYCES CEREVISIAE S288C 35S PRE-RIBOSOMAL
626	6xqj-A	2.8	5.2	71	173	8	MOLECULE: PROTEIN VPR,UV EXCISION REPAIR PROTEIN RAD23 HOMO
627	6y37-C	2.8	3.1	56	118	7	MOLECULE: HAPB;

628	1sgm-A	2.8	12.1	63	184	5	MOLECULE: PUTATIVE HTH-TYPE TRANSCRIPTIONAL REGULATOR YXAF;
629	5xfs-A	2.8	3.8	42	78	2	MOLECULE: PE FAMILY PROTEIN PE8;
630	6ei9-A	2.8	4.5	58	306	10	MOLECULE: TRNA-DIHYDROURIDINE SYNTHASE B;
631	7oca-E	2.8	4.7	57	158	21	MOLECULE: GLUTAMATE RECEPTOR 1;
632	6k4e-B	2.8	2.9	54	246	13	MOLECULE: HAMP DOMAIN-CONTAINING PROTEIN;
633	2o7t-A	2.8	11.2	59	188	10	MOLECULE: TRANSCRIPTIONAL REGULATOR;
634	5gvv-A	2.8	12.5	81	392	2	MOLECULE: GLYCOSYL TRANSFERASE FAMILY 8;
635	6nr8-4	2.8	4.7	43	104	9	MOLECULE: PREFOLDIN SUBUNIT 1;
636	2yfb-B	2.8	7	62	238	8	MOLECULE: METHYL-ACCEPTING CHEMOTAXIS TRANSDUCER;
637	2p22-A	2.8	5	44	168	9	MOLECULE: SUPPRESSOR PROTEIN STP22 OF TEMPERATURE-SENSITIVE
638	4he8-D	2.8	4.3	45	160	11	MOLECULE: NADH-QUINONE OXIDOREDUCTASE SUBUNIT 7;
639	6hd8-B	2.8	6.4	59	232	10	MOLECULE: NANOBODY,MALTOSE/MALTODEXTRIN-BINDING PERIPLASMIC
640	6n7o-B	2.8	2.8	37	39	16	MOLECULE: GIL01 GP7;
641	5z9w-A	2.8	11.5	50	388	10	MOLECULE: EBOLAVIRUS NUCLEOPROTEIN (RESIDUES 19-406);
642	6r24-A	2.8	12.7	79	159	5	MOLECULE: TRANSPOSON TY3-I GAG-POL POLYPROTEIN;
643	2bma-A	2.8	5	60	467	8	MOLECULE: GLUTAMATE DEHYDROGENASE (NADP+);
644	1te5-A	2.8	4.1	59	253	8	MOLECULE: CONSERVED HYPOTHETICAL PROTEIN;
645	7rsf-A	2.8	11.7	68	380	10	MOLECULE: ACETYLORNITHINE DEACETYLASE;
646	2qeb-B	2.8	4.2	82	145	7	MOLECULE: D7R4 PROTEIN;
647	5an6-A	2.8	10.7	67	123	1	MOLECULE: CRISPR-ASSOCIATED PROTEIN, CSM2 FAMILY;
648	5wwl-N	2.8	5.7	59	155	2	MOLECULE: CENTROMERE PROTEIN MIS12;
649	6thk-A	2.8	4.6	51	466	6	MOLECULE: PYOCIN S5;
650	1ixs-B	2.8	7.8	68	315	10	MOLECULE: HOLLIDAY JUNCTION DNA HELICASE RUVA;
651	2j5y-A	2.8	2.2	38	61	3	MOLECULE: PEPTOSTREPTOCOCCAL ALBUMIN-BINDING PROTEIN;
652	7ogp-E	2.8	7.7	60	377	7	MOLECULE: PHIKZ055,PHIKZ056.1;
653	2peg-B	2.8	3.3	63	139	3	MOLECULE: HEMOGLOBIN SUBUNIT ALPHA;
654	1lrz-A	2.8	13.1	77	400	5	MOLECULE: FACTOR ESSENTIAL FOR EXPRESSION OF METHICILLIN
655	2luh-A	2.8	7.3	59	167	7	MOLECULE: VACUOLAR PROTEIN SORTING-ASSOCIATED PROTEIN VTA1;
656	6rd4-1	2.8	13.2	82	595	6	MOLECULE: ASA-10: POLYTOMELLA F-ATP SYNTHASE ASSOCIATED SUB
657	7emf-D	2.8	6.9	60	158	13	MOLECULE: MEDIATOR OF RNA POLYMERASE II TRANSCRIPTION SUBUN
658	4eyw-B	2.8	4.1	46	634	4	MOLECULE: CARNITINE O-PALMITOYLTRANSFERASE 2, MITOCHONDRIAL
659	6ynw-H	2.8	3.6	44	75	5	MOLECULE: SUBUNIT C;
660	4ghl-A	2.8	3.3	62	132	8	MOLECULE: SHORT PALINDROMIC RNA AGACAGCAUAUGCUGUCU;
661	5y9o-A	2.8	6.3	61	401	8	MOLECULE: WIPA;
662	4bx9-C	2.8	5.2	53	95	11	MOLECULE: VACUOLAR PROTEIN SORTING-ASSOCIATED PROTEIN 33A;

663	6ae9-A	2.8	2.9	53	253	11	MOLECULE: PROBABLE PROTEIN PHOSPHATASE 2C 1;
664	6f1e-A	2.8	6.3	60	143	3	MOLECULE: INTERFERON GAMMA;
665	6lqo-L	2.8	5.7	49	94	10	MOLECULE: CYTOPLASMIC ENVELOPMENT PROTEIN 1;
666	3rpm-A	2.8	11.1	85	450	13	MOLECULE: BETA-N-ACETYL-HEXOSAMINIDASE;
667	2hl7-A	2.8	2.5	39	82	8	MOLECULE: CYTOCHROME C-TYPE BIOGENESIS PROTEIN CCMH;
668	3h36-A	2.8	3.2	47	78	11	MOLECULE: POLYRIBONUCLEOTIDE NUCLEOTIDYLTRANSFERASE;
669	2i1k-A	2.8	7.9	58	545	7	MOLECULE: MOESIN;
670	1wxq-A	2.8	10.1	78	344	14	MOLECULE: GTP-BINDING PROTEIN;
671	2vj4-A	2.8	9.9	62	292	13	MOLECULE: PROTEIN MXIC;
672	5hx2-A	2.8	13.5	82	1030	12	MOLECULE: BASEPLATE WEDGE PROTEIN GP7;
673	2klz-A	2.8	5.5	41	46	12	MOLECULE: ATAXIN-3;
674	6m9k-D	2.8	3.1	50	67	8	MOLECULE: EXONUCLEASE;
675	2khq-A	2.8	4.2	40	110	5	MOLECULE: INTEGRASE;
676	6bmt-B	2.8	3.4	49	70	4	MOLECULE: MYELOPEROXIDASE;
677	5b1q-A	2.8	8	74	448	8	MOLECULE: U14 PROTEIN;
678	7ou2-B	2.8	9.6	80	650	10	MOLECULE: DNA MISMATCH REPAIR PROTEIN MUTS;
679	6l1k-A	2.8	3.4	59	384	10	MOLECULE: NADH-DEPENDENT BUTANOL DEHYDROGENASE A;
680	6uuj-G	2.8	4.3	42	74	2	MOLECULE: PE FAMILY IMMUNOMODULATOR PE5;
681	3i3l-A	2.8	15.3	80	550	11	MOLECULE: ALKYLHALIDASE CMLS;
682	5gpo-B	2.8	7.5	57	124	7	MOLECULE: SENSOR PROTEIN CZCS;
683	5m4s-A	2.8	3.3	64	209	6	MOLECULE: TRANSCRIPTION INITIATION FACTOR IIA SUBUNIT 2,TRA
684	4v1a-w	2.8	3.9	68	387	12	MOLECULE: MITORIBOSOMAL PROTEIN ML37, MRPL37;
685	5gn1-D	2.8	8.4	76	312	12	MOLECULE: ATP-DEPENDENT HELICASE FUN30;
686	2ipc-A	2.8	4	75	939	8	MOLECULE: PREPROTEIN TRANSLOCASE SECA SUBUNIT;
687	7ac1-A	2.8	2.9	59	75	7	MOLECULE: TRANSCRIPTION INITIATION FACTOR TFIID SUBUNIT 4;
688	7cun-G	2.8	12.3	97	895	8	MOLECULE: INTEGRATOR COMPLEX SUBUNIT 1;
689	6th1-R	2.8	12.8	96	360	7	MOLECULE: IMMEDIATE EARLY PROTEIN 1;
690	4qvh-A	2.8	11.3	83	596	8	MOLECULE: MALTOSE-BINDING PERIPLASMIC PROTEIN, 4'-PHOSPHOPA
691	3lay-J	2.8	2.9	42	81	0	MOLECULE: ZINC RESISTANCE-ASSOCIATED PROTEIN;
692	4uyb-A	2.8	11.9	81	400	11	MOLECULE: SEC14-LIKE PROTEIN 3;
693	6mrr-A	2.8	3.3	46	68	7	MOLECULE: FOLDIT1;
694	6s7t-F	2.8	12.9	68	250	4	MOLECULE: DOLICHYL-DIPHOSPHOOLIGOSACCHARIDE--PROTEIN
695	4djg-A	2.8	4	41	49	7	MOLECULE: PLECTIN-RELATED PROTEIN;
696	4y2i-A	2.8	3.1	42	65	7	MOLECULE: PUTATIVE METAL-BINDING TRANSPORT PROTEIN;
697	1skv-A	2.8	5.2	40	64	10	MOLECULE: HYPOTHETICAL 7.5 KDA PROTEIN;

698	4uy3-A	2.8	7.1	82	194	11	MOLECULE: SEPTATION RING FORMATION REGULATOR EZRA;
699	6wuc-H	2.8	12.2	73	179	10	MOLECULE: INNER KINETOCHORE SUBUNIT MCM16;
700	6arh-B	2.8	12.2	57	303	11	MOLECULE: N-ACETYLNEURAMINATE LYASE;
701	5oqm-n	2.8	7.3	57	136	5	MOLECULE: DNA-DIRECTED RNA POLYMERASE II SUBUNIT RPB1;
702	6rfq-P	2.8	11.9	72	123	7	MOLECULE: SUBUNIT NUAM OF NADH:UBIQUINONE OXIDOREDUCTASE (C
703	3jbl-A	2.8	16.5	82	909	9	MOLECULE: NLR FAMILY CARD DOMAIN-CONTAINING PROTEIN 4;
704	5h5v-A	2.8	4.8	48	352	8	MOLECULE: FLAGELLAR HOOK-ASSOCIATED PROTEIN 2;
705	2nd2-A	2.8	2.3	39	44	5	MOLECULE: DE NOVO MINI PROTEIN HHH_06;
706	6rre-D	2.8	5.2	90	427	8	MOLECULE: ADENOSINE MONOPHOSPHATE-PROTEIN HYDROLASE SIDD;
707	6ogy-K	2.8	12.6	69	827	6	MOLECULE: RNA-DEPENDENT RNA POLYMERASE OF ROTAVIRUS A;
708	7jhj-R	2.8	4.3	64	277	6	MOLECULE: GUANINE NUCLEOTIDE-BINDING PROTEIN G(I) SUBUNIT A
709	5cd4-I	2.8	8.9	69	494	9	MOLECULE: CRISPR SYSTEM CASCADE SUBUNIT CASE;
710	1qgi-A	2.8	3.1	66	259	9	MOLECULE: PROTEIN (CHITOSANASE);
711	4aim-A	2.8	24.7	72	698	6	MOLECULE: POLYRIBONUCLEOTIDE NUCLEOTIDYLTRANSFERASE;
712	2z3x-A	2.8	3.8	44	56	9	MOLECULE: SMALL, ACID-SOLUBLE SPORE PROTEIN C;
713	4nsm-A	2.8	5.2	39	71	13	MOLECULE: COLLAGEN-LIKE PROTEIN SCLB;
714	6kyf-A	2.8	4.6	59	137	8	MOLECULE: ACRF11;
715	4af8-A	2.8	10.9	60	333	5	MOLECULE: METACASPASE MCA2;
716	1pix-A	2.8	6	57	586	9	MOLECULE: GLUTACONYL-COA DECARBOXYLASE A SUBUNIT;
717	5k12-A	2.8	10.2	74	294	3	MOLECULE: GLUTAMATE DEHYDROGENASE 1, MITOCHONDRIAL;
718	3c3w-A	2.8	8.9	66	211	5	MOLECULE: TWO COMPONENT TRANSCRIPTIONAL REGULATORY PROTEIN
719	4or2-B	2.8	4.9	80	366	3	MOLECULE: SOLUBLE CYTOCHROME B562, METABOTROPIC GLUTAMATE R
720	6c03-A	2.8	5	54	138	6	MOLECULE: PUTATIVE RNA POLYMERASE ECF-SUBFAMILY SIGMA FACTO
721	6r3q-A	2.8	20	83	841	5	MOLECULE: ADENYLATE CYCLASE 9;
722	7onu-F	2.8	11.4	80	276	10	MOLECULE: 3-HYDROXYACYL-COA DEHYDROGENASE TYPE-2;
723	4rta-B	2.8	2.5	41	71	7	MOLECULE: PROTEIN DPY-30 HOMOLOG;
724	6yj4-J	2.8	8.5	60	185	7	MOLECULE: NADH-UBIQUINONE OXIDOREDUCTASE CHAIN 3;
725	5j1f-A	2.8	10.2	76	188	3	MOLECULE: PLECTIN,PLECTIN;
726	5tck-A	2.8	3.7	48	139	8	MOLECULE: UNCHARACTERIZED PROTEIN;
727	2ota-B	2.8	2.5	50	61	12	MOLECULE: UPF0352 PROTEIN CPS_2611;
728	3x38-B	2.8	3.3	49	79	12	MOLECULE: MITOCHONDRIAL MORPHOGENESIS PROTEIN SLD7;
729	6c70-A	2.8	9.1	69	388	10	MOLECULE: ODORANT RECEPTOR;
730	4lid-A	2.8	8.2	56	86	7	MOLECULE: A-100;
731	7p47-B	2.8	4.9	45	69	18	MOLECULE: STRUCTURAL MAINTENANCE OF CHROMOSOMES PROTEIN 5;
732	2c5z-A	2.8	4.8	51	93	8	MOLECULE: SET DOMAIN PROTEIN 2;

733	2apl-A	2.8	9.2	48	149	8	MOLECULE: HYPOTHETICAL PROTEIN PG0816;
734	6wc3-B	2.8	4.2	47	94	23	MOLECULE: PROTEIN TRANSPORT PROTEIN TIP20;
735	6twr-A	2.8	2.1	37	169	3	MOLECULE: BETA-GLUCOSIDE BGL OPERON ANTITERMINATOR BGLG FAM
736	2l35-A	2.8	3.9	44	62	5	MOLECULE: DAP12-NKG2C_TM;
737	5j2l-A	2.8	5	53	76	13	MOLECULE: PROTEIN DESIGN 2L4HC2_11;
738	4c0j-A	2.8	17.1	96	404	8	MOLECULE: MITOCHONDRIAL RHO GTPASE;
739	5yfp-B	2.8	8	72	927	11	MOLECULE: EXOCYST COMPLEX COMPONENT SEC3;
740	7kpv-B	2.8	11.1	81	290	9	MOLECULE: MEIOTIC MRNA STABILITY PROTEIN KINASE SSN3;
741	6ozu-A	2.8	7.7	60	235	15	MOLECULE: EUKARYOTIC TRANSLATION INITIATION FACTOR 4 GAMMA
742	3gor-A	2.8	4	81	157	4	MOLECULE: PUTATIVE METAL-DEPENDENT HYDROLASE;
743	4ad8-A	2.8	4.3	62	452	5	MOLECULE: DNA REPAIR PROTEIN RECN;
744	4b94-A	2.8	5.4	55	141	11	MOLECULE: DUAL SPECIFICITY PROTEIN KINASE TTK;
745	3pvs-D	2.8	12.5	97	424	7	MOLECULE: REPLICATION-ASSOCIATED RECOMBINATION PROTEIN A;
746	6iva-A	2.7	5.7	106	420	9	MOLECULE: OXALOACETATE DECARBOXYLASE BETA CHAIN;
747	6yiz-A	2.7	4.5	46	211	11	MOLECULE: TRANSCRIPTIONAL REGULATOR MVFR;
748	6hpn-A	2.7	4.3	61	212	5	MOLECULE: ANTIGEN, P35;
749	2rkh-A	2.7	7.1	69	164	7	MOLECULE: PUTATIVE APHA-LIKE TRANSCRIPTION FACTOR;
750	6tj1-C	2.7	5.3	43	74	14	MOLECULE: DE NOVO DESIGNED WSHC6;
751	7mhz-A	2.7	5.7	97	490	7	MOLECULE: SONIC HEDGEHOG PROTEIN N-PRODUCT PEPTIDE;
752	6t8h-B	2.7	7.3	106	1188	9	MOLECULE: DNA POLYMERASE SLIDING CLAMP;
753	6vme-C	2.7	4.8	48	67	6	MOLECULE: TUMOR SUSCEPTIBILITY GENE 101 PROTEIN;
754	7l48-A	2.7	14	83	522	4	MOLECULE: CAS12F;
755	2rq6-A	2.7	10.2	58	138	5	MOLECULE: ATP SYNTHASE EPSILON CHAIN;
756	4jle-B	2.7	3.7	47	159	9	MOLECULE: PHIST;
757	6h0q-A	2.7	2.8	55	102	11	MOLECULE: TYPE I MODULAR POLYKETIDE SYNTHASE;
758	3o0y-A	2.7	3.8	64	585	3	MOLECULE: LIPOPROTEIN;
759	6zbj-B	2.7	16.9	100	501	6	MOLECULE: PRECURSOR OF THE MAJOR MEROZOITE SURFACE ANTIGENS
760	5ean-A	2.7	15.5	73	1051	8	MOLECULE: DNA REPLICATION ATP-DEPENDENT HELICASE/NUCLEASE D
761	6qm7-C	2.7	6.1	70	276	11	MOLECULE: PROTEASOME ALPHA1 CHAIN;
762	2imd-A	2.7	10.2	72	203	6	MOLECULE: 2-HYDROXYCHROMENE-2-CARBOXYLATE ISOMERASE;
763	5yx5-B	2.7	4.4	63	270	8	MOLECULE: 49 PROTEIN;
764	5h11-A	2.7	8.9	75	494	13	MOLECULE: UNCHARACTERIZED PROTEIN;
765	2ptm-A	2.7	7.7	43	192	14	MOLECULE: HYPERPOLARIZATION-ACTIVATED (IH) CHANNEL;
766	4pac-A	2.7	12.1	69	153	9	MOLECULE: HISTIDINE-CONTAINING PHOSPHOTRANSFER PROTEIN 2;
767	4r0g-A	2.7	11.1	84	253	11	MOLECULE: UNCHARACTERIZED PROTEIN;

768	5b0l-B	2.7	11.1	93	334	3	MOLECULE: MOEN5,DNA-BINDING PROTEIN 7D;
769	6wm6-A	2.7	13.6	82	536	4	MOLECULE: EXTRACELLULAR SOLUTE-BINDING PROTEIN, FAMILY 5;
770	5z79-C	2.7	6.9	77	599	8	MOLECULE: HYDROXYMETHYLDIHYDROPTERIN PYROPHOSPHOKINASE-
771	4tko-B	2.7	6.5	61	326	5	MOLECULE: EMRA;
772	6rd4-4	2.7	5.4	81	290	12	MOLECULE: ASA-10: POLYTOMELLA F-ATP SYNTHASE ASSOCIATED SUB
773	2khm-A	2.7	3.9	50	140	10	MOLECULE: FIBROIN-3;
774	4u9r-A	2.7	3	55	147	9	MOLECULE: CZCP CATION EFFLUX P1-ATPASE;
775	5z2c-A	2.7	10.1	89	445	3	MOLECULE: ALPHA-PROTEIN KINASE 1;
776	7awt-D	2.7	14.3	78	589	6	MOLECULE: NADH-QUINONE OXIDOREDUCTASE SUBUNIT B;
777	1flc-B	2.7	8.1	60	162	8	MOLECULE: HAEMAGGLUTININ-ESTERASE-FUSION GLYCOPROTEIN;
778	6dmp-B	2.7	4.6	49	82	14	MOLECULE: DESIGNED ORTHOGONAL PROTEIN DHD13_XAAA_A;
779	5nbs-A	2.7	11.4	87	842	2	MOLECULE: BETA-GLUCOSIDASE;
780	5dpl-A	2.7	14.2	103	521	5	MOLECULE: PROTEIN LYSINE METHYLTRANSFERASE 2;
781	6s6b-I	2.7	9.6	89	284	4	MOLECULE: CRISPR-ASSOCIATED PROTEIN, CMR5 FAMILY;
782	4b3i-A	2.7	9.9	96	730	10	MOLECULE: FATTY ACID BETA-OXIDATION COMPLEX ALPHA-CHAIN FAD
783	6kf4-G	2.7	5.5	37	166	8	MOLECULE: DNA-DIRECTED RNA POLYMERASE SUBUNIT;
784	6zzx-F	2.7	5.8	45	165	9	MOLECULE: PHOTOSYSTEM I P700 CHLOROPHYLL A APOPROTEIN A1;
785	6elc-A	2.7	7.9	61	356	10	MOLECULE: VARIANT SURFACE GLYCOPROTEIN;
786	6zdx-A	2.7	3	51	100	8	MOLECULE: RIFIN;
787	5wtn-A	2.7	9.4	65	268	8	MOLECULE: REPLICATION INITIATION AND MEMBRANE ATTACHMENT PR
788	2q0y-A	2.7	3.6	40	153	10	MOLECULE: GCN5-RELATED N-ACETYLTRANSFERASE;
789	2lyi-A	2.7	5.4	90	167	9	MOLECULE: PROTEIN (ENTITY);
790	6p18-Q	2.7	3.6	59	156	3	MOLECULE: DNA (67-MER) FRAGMENT CARRYING PHAGE-21 PR' PROMO
791	5awf-A	2.7	5	73	385	7	MOLECULE: FES CLUSTER ASSEMBLY PROTEIN SUFB;
792	3k33-A	2.7	3.4	66	124	8	MOLECULE: DEATH ON CURING PROTEIN;
793	6yse-A	2.7	4.8	37	46	11	MOLECULE: GP4;
794	4i0x-K	2.7	5.6	54	77	4	MOLECULE: ESAT-6-LIKE PROTEIN MAB_3112;
795	5y6o-D	2.7	15.8	50	108	4	MOLECULE: DEATH DOMAIN-ASSOCIATED PROTEIN 6,TRANSCRIPTIONAL
796	5xj5-A	2.7	10.8	71	200	7	MOLECULE: GLYCEROL-3-PHOSPHATE ACYLTRANSFERASE;
797	7c4o-A	2.7	4	39	48	8	MOLECULE: TRANSCRIPTION FACTOR HES-1;
798	6g2d-C	2.7	20.8	95	2121	5	MOLECULE: ACETYL-COA CARBOXYLASE 1;
799	4rp5-A	2.7	6.3	47	94	4	MOLECULE: DISKS LARGE 1 TUMOR SUPPRESSOR PROTEIN;
800	1iuf-A	2.7	8	66	144	6	MOLECULE: CENTROMERE ABP1 PROTEIN;
801	5idr-D	2.7	16.5	64	221	9	MOLECULE: DSBA-LIKE PROTEIN;
802	3p3o-A	2.7	2.7	57	416	11	MOLECULE: CYTOCHROME P450;

803	3nmz-A	2.7	17.9	78	406	9	MOLECULE: APC VARIANT PROTEIN;
804	5olk-A	2.7	3.4	64	395	2	MOLECULE: RIBONUCLEOSIDE-DIPHOSPHATE REDUCTASE, BETA SUBUNI
805	6iqc-A	2.7	2.5	45	103	9	MOLECULE: DNA-BINDING PROTEIN SSO0352;
806	5j5l-A	2.7	4.4	49	152	8	MOLECULE: UNCHARACTERIZED PROTEIN;
807	4fbj-A	2.7	3	48	250	13	MOLECULE: HYPOTHETICAL PROTEIN;
808	4myy-B	2.7	2.9	54	84	15	MOLECULE: CURG, CURH FUSION PROTEIN;
809	3bru-A	2.7	12.6	73	191	4	MOLECULE: REGULATORY PROTEIN, TETR FAMILY;
810	3p01-A	2.7	4.3	49	178	8	MOLECULE: TWO-COMPONENT RESPONSE REGULATOR;
811	5f15-A	2.7	5	91	541	2	MOLECULE: 4-AMINO-4-DEOXY-L-ARABINOSE (L-ARA4N) TRANSFERASE
812	2gnx-A	2.7	8.2	52	288	6	MOLECULE: HYPOTHETICAL PROTEIN;
813	6n04-B	2.7	13.6	76	366	9	MOLECULE: ABSH3;
814	6v6b-B	2.7	13	59	572	7	MOLECULE: GAMMA-TUBULIN COMPLEX COMPONENT 3;
815	4x00-A	2.7	4.2	64	273	9	MOLECULE: PUTATIVE HYDROLASE;
816	6n7m-A	2.7	6.2	47	63	6	MOLECULE: HYPOTHETICAL PROTEIN CD630_05490;
817	6m7d-A	2.7	13.6	97	412	5	MOLECULE: NUCLEOPROTEIN;
818	6jo5-F	2.7	5.8	45	165	9	MOLECULE: PHOTOSYSTEM I P700 CHLOROPHYLL A APOPROTEIN A1;
819	6ybw-y	2.7	6.7	62	162	6	MOLECULE: 40S RIBOSOMAL PROTEIN S4, X ISOFORM;
820	6e3d-A	2.7	3.5	61	510	7	MOLECULE: PERIPLASMIC DIPEPTIDE-BINDING LIPOPROTEIN DPPA;
821	6noc-A	2.7	15.6	84	402	10	MOLECULE: FEM-3 MRNA-BINDING FACTOR 2;
822	6k9k-A	2.7	15	87	2501	3	MOLECULE: SERINE-PROTEIN KINASE ATM;
823	1s12-A	2.7	3.6	57	94	9	MOLECULE: HYPOTHETICAL PROTEIN TM1457;
824	5k2a-A	2.7	6.5	74	398	12	MOLECULE: ADENOSINE RECEPTOR A2A/SOLUBLE CYTOCHROME B562 CH
825	4c2e-A	2.7	9.1	53	435	9	MOLECULE: CARBOXY-TERMINAL PROCESSING PROTEASE CTPB;
826	2qup-A	2.7	3.9	53	119	8	MOLECULE: BH1478 PROTEIN;
827	4g80-I	2.7	7.2	62	139	3	MOLECULE: VOLTAGE-SENSOR CONTAINING PHOSPHATASE;
828	3mem-A	2.7	17.1	84	453	7	MOLECULE: PUTATIVE SIGNAL TRANSDUCTION PROTEIN;
829	6ypu-p	2.7	7.4	44	88	14	MOLECULE: 16S RIBOSOMAL RNA;
830	3jac-A	2.7	13	67	918	0	MOLECULE: PIEZO-TYPE MECHANOSENSITIVE ION CHANNEL COMPONENT
831	1qrj-A	2.7	10.5	91	214	10	MOLECULE: HTLV-I CAPSID PROTEIN;
832	5ebz-A	2.7	13.3	91	655	9	MOLECULE: INHIBITOR OF NUCLEAR FACTOR KAPPA-B KINASE SUBUNI
833	5gox-A	2.7	7.2	61	181	11	MOLECULE: DNA REPAIR PROTEIN RAD50;
834	2opd-A	2.7	4.1	51	121	18	MOLECULE: PILX;
835	7eu3-F	2.7	5.2	74	671	9	MOLECULE: NAD(P)H-QUINONE OXIDOREDUCTASE SUBUNIT 1, CHLOROP
836	5xe7-B	2.7	8.3	101	280	6	MOLECULE: ECF RNA POLYMERASE SIGMA FACTOR SIGJ;
837	3ucq-A	2.7	11.7	77	651	9	MOLECULE: AMYLOSUCRASE;

838	2pih-A	2.7	6.3	57	123	9	MOLECULE: PROTEIN YMCA;
839	6to1-A	2.7	10.5	63	566	6	MOLECULE: MINOR FIMBRIUM SUBUNIT MFA5;
840	4gc5-A	2.7	2.7	53	318	4	MOLECULE: DIMETHYLADENOSINE TRANSFERASE 1, MITOCHONDRIAL;
841	5m0n-A	2.7	8.4	69	423	6	MOLECULE: TERMINAL OLEFIN-FORMING FATTY ACID DECARBOXYLASE;
842	6uak-A	2.7	8.9	66	298	9	MOLECULE: SAM DEPENDENT METHYLTRANSFERASE LAHSB;
843	6xgx-A	2.7	8.4	86	402	7	MOLECULE: MUTATOR FAMILY TRANSPOSASE;
844	4kpk-A	2.7	10.4	64	265	9	MOLECULE: ENOYL-COA HYDRATASE/ISOMERASE;
845	3f1i-H	2.7	8.4	56	98	13	MOLECULE: HEPATOCYTE GROWTH FACTOR-REGULATED TYROSINE
846	6epc-H	2.7	10.8	58	396	3	MOLECULE: PROTEASOME SUBUNIT ALPHA TYPE-6;
847	4i5s-B	2.7	7.4	61	395	5	MOLECULE: PUTATIVE HISTIDINE KINASE COVS; VICK-LIKE PROTEIN
848	2k3n-A	2.7	3.9	76	160	8	MOLECULE: TUSP1;
849	4qlb-D	2.7	5.1	59	651	10	MOLECULE: PROBABLE GLYCOGEN [STARCH] SYNTHASE;
850	5nug-A	2.7	6	70	2920	10	MOLECULE: CYTOPLASMIC DYNEIN 1 HEAVY CHAIN 1;
851	7asl-A	2.7	3.7	85	235	8	MOLECULE: GAG PROTEIN;
852	6vek-A	2.7	9.8	72	321	7	MOLECULE: CONTACT-DEPENDENT TOXIN CDIA;
853	6i3m-E	2.7	4.4	51	354	10	MOLECULE: TRANSLATION INITIATION FACTOR EIF-2B SUBUNIT ALPH
854	4zy8-A	2.7	3.5	49	146	6	MOLECULE: PROTEIN LST4;
855	6qrj-A	2.7	14.5	55	463	4	MOLECULE: HYBRID KINASE;
856	6vgc-D	2.7	8	58	155	9	MOLECULE: 5-LIPOXYGENASE-ACTIVATING PROTEIN;
857	7jtk-M	2.7	2.1	36	47	11	MOLECULE: FLAGELLAR RADIAL SPOKE PROTEIN 1;
858	7njn-C	2.7	12.7	73	536	10	MOLECULE: ATP SYNTHASE SUBUNIT ALPHA;
859	5xqi-B	2.7	8.7	78	276	3	MOLECULE: PROTEIN ROGDI HOMOLOG;
860	5v5t-A	2.7	7	77	306	8	MOLECULE: CONSERVED DOMAIN PROTEIN;
861	6zyx-C	2.7	9.5	48	278	6	MOLECULE: DYNEIN HEAVY CHAIN, OUTER ARM PROTEIN;
862	2i7u-A	2.7	3.5	42	62	5	MOLECULE: FOUR-ALPHA-HELIX BUNDLE;
863	1cm5-A	2.7	3.3	59	759	5	MOLECULE: PROTEIN (PYRUVATE FORMATE-LYASE);
864	5mnt-B	2.7	22.1	80	421	8	MOLECULE: A2 MATURATION PROTEIN;
865	6ys8-C	2.7	5.5	43	60	7	MOLECULE: GLDM;
866	1oks-A	2.7	3.2	45	53	13	MOLECULE: RNA POLYMERASE ALPHA SUBUNIT;
867	6qm5-A	2.7	10.1	81	673	5	MOLECULE: PREDICTED PROTEIN;
868	6cpu-A	2.7	7.5	89	526	4	MOLECULE: PHOSPHODIESTERASE;
869	4xmn-E	2.7	12.8	96	896	4	MOLECULE: PROTEIN TRANSPORT PROTEIN SEC13;
870	3sjr-A	2.7	3.7	54	126	4	MOLECULE: UNCHARACTERIZED PROTEIN;
871	7ah0-A	2.7	6.1	44	104	2	MOLECULE: 4D2;
872	2qsr-A	2.7	3.5	53	161	9	MOLECULE: TRANSCRIPTION-REPAIR COUPLING FACTOR;

873	4uis-B	2.7	9.7	75	241	3	MOLECULE: GAMMA-SECRETASE;
874	5o4j-A	2.7	7.6	63	265	5	MOLECULE: HCGC;
875	4ql6-B	2.7	3.2	60	509	10	MOLECULE: CARBOXY-TERMINAL PROCESSING PROTEASE;
876	7d3u-E	2.7	4.9	52	110	8	MOLECULE: MONOVALENT NA+/H+ ANTIPORTER SUBUNIT D;
877	6v4m-A	2.7	7.2	66	163	2	MOLECULE: BCL-2;
878	1r71-B	2.7	3.5	44	116	14	MOLECULE: 5'-D(*AP*(BRU)
879	5e6g-B	2.7	3.5	49	114	12	MOLECULE: DE NOVO DESIGNED PROTEIN CA01;
880	6jly-G	2.7	6.1	66	363	6	MOLECULE: TRANSLATION INITIATION FACTOR EIF-2B SUBUNIT ALPH
881	5vp3-A	2.7	3.9	58	195	7	MOLECULE: MNEMIOPSIN 1;
882	6njw-A	2.7	4.1	66	208	9	MOLECULE: XCC_CTR_PT;
883	1q16-A	2.7	5.7	83	1244	5	MOLECULE: RESPIRATORY NITRATE REDUCTASE 1 ALPHA CHAIN;
884	6w1h-A	2.7	11.8	78	450	5	MOLECULE: HYDROXYGLUTARATE SYNTHASE;
885	3sqn-A	2.7	6.8	75	468	11	MOLECULE: CONSERVED DOMAIN PROTEIN;
886	6bs9-A	2.7	4.2	49	130	6	MOLECULE: STAGE III SPORULATION PROTEIN AB;
887	6rql-A	2.7	9	86	1542	9	MOLECULE: TEMPLATE STRAND;
888	4ysx-G	2.7	14	77	155	8	MOLECULE: SUCCINATE DEHYDROGENASE FLAVOPROTEIN;
889	2jvw-A	2.7	4	62	82	10	MOLECULE: UNCHARACTERIZED PROTEIN;
890	5w10-A	2.7	4.3	44	173	9	MOLECULE: CGMP-SPECIFIC PHOSPHODIESTERASE;
891	3k29-A	2.7	6.3	55	162	15	MOLECULE: PUTATIVE UNCHARACTERIZED PROTEIN;
892	5ipx-A	2.7	8.3	84	282	7	MOLECULE: ORF49 PROTEIN;
893	7cm9-A	2.7	9.8	85	693	12	MOLECULE: DMSP LYASE;
894	4yo5-A	2.7	9.4	52	131	4	MOLECULE: TSSA;
895	7oa5-B	2.7	11.1	52	186	6	MOLECULE: HOLLIDAY JUNCTION ATP-DEPENDENT DNA HELICASE RUVA
896	6pw7-A	2.7	10.1	62	161	6	MOLECULE: STROMAL INTERACTION MOLECULE 1;
897	3tvi-D	2.7	16.3	77	439	3	MOLECULE: ASPARTOKINASE;
898	5cqq-B	2.7	4.1	51	75	12	MOLECULE: REGULATORY PROTEIN ZESTE;
899	5tub-A	2.7	4.3	81	335	4	MOLECULE: SHARK TBC1D15 GTPASE-ACTIVATING PROTEIN;
900	6iuy-A	2.7	10.6	87	585	5	MOLECULE: GLYCEROL-3-PHOSPHATE DEHYDROGENASE [NAD(+)];
901	7dms-A	2.7	3.5	34	94	18	MOLECULE: FE(II)-BINDING EFFECTOR;
902	4hb1-A	2.7	4.6	39	44	5	MOLECULE: DHP1;
903	4uig-A	2.7	8.3	55	91	11	MOLECULE: COPPER SENSITIVE OPERON REPRESSOR;
904	6k2e-A	2.7	7.1	49	88	14	MOLECULE: CRISPR/CAS2 PROTEIN;
905	7mtl-B	2.7	3.5	76	162	8	MOLECULE: COLIBACTIN SELF-PROTECTION PROTEIN CLBS;
906	6qzh-A	2.7	5.4	77	744	10	MOLECULE: HUMAN CHEMOKINE RECEPTOR 7;
907	6n0v-A	2.7	10.3	85	395	7	MOLECULE: TRNA LIGASE;

908	3mnl-A	2.7	12.2	75	188	8	MOLECULE: TRANSCRIPTIONAL REGULATORY PROTEIN (PROBABLY TETR
909	4geh-D	2.7	5.2	51	69	0	MOLECULE: PROGRAMMED CELL DEATH PROTEIN 10;
910	6hqa-A	2.6	6.3	97	887	9	MOLECULE: TAF2;
911	6mcp-A	2.6	5.5	44	520	7	MOLECULE: LEGK7;
912	4axd-A	2.6	9.4	76	433	1	MOLECULE: INOSITOL-PENTAKISPHOSPHATE 2-KINASE;
913	5v7p-A	2.6	10.4	69	281	6	MOLECULE: PROTEIN-S-ISOPRENYLCYSTEINE O-METHYLTRANSFERASE;
914	4ney-B	2.6	12.1	68	173	10	MOLECULE: ENGINEERED PROTEIN OR277;
915	5ea1-B	2.6	3.2	64	128	11	MOLECULE: TRANSCRIPTION ACTIVATOR BRG1;
916	6z8h-A	2.6	9.3	71	351	8	MOLECULE: VARIANT SURFACE GLYCOPROTEIN MITAT 1.13;
917	4atv-A	2.6	4.8	83	381	5	MOLECULE: NA(+)/H(+) ANTIPORTER NHAA;
918	5y06-A	2.6	7.3	60	235	8	MOLECULE: MSMEG_4306;
919	1otr-A	2.6	2.8	42	49	10	MOLECULE: PROTEIN CUE2;
920	6wti-D	2.6	7.8	55	99	9	MOLECULE: CYTOCHROME O UBIQUINOL OXIDASE, SUBUNIT I;
921	6jbh-C	2.6	15.9	87	267	6	MOLECULE: TARH;
922	6c0f-w	2.6	2.7	40	70	10	MOLECULE: SACCHAROMYCES CEREVISIAE S288C 35S PRE-RIBOSOMAL
923	3a8t-A	2.6	6.3	56	289	7	MOLECULE: ADENYLATE ISOPENTENYLTRANSFERASE;
924	7b9c-A	2.6	9.2	94	870	4	MOLECULE: SPLICING FACTOR 3B SUBUNIT 3;
925	6ff6-A	2.6	8.6	86	222	10	MOLECULE: BRIC1;
926	1cii-A	2.6	2.2	54	602	9	MOLECULE: COLICIN IA;
927	3iuk-A	2.6	7.4	71	552	1	MOLECULE: UNCHARACTERIZED PROTEIN;
928	2ld6-A	2.6	3.8	58	133	5	MOLECULE: CHEMOTAXIS PROTEIN CHEA;
929	4yy2-A	2.6	4.1	56	99	9	MOLECULE: DTOR_3X33L;
930	3jcf-A	2.6	3.1	40	349	5	MOLECULE: MAGNESIUM TRANSPORT PROTEIN CORA;
931	6q6g-O	2.6	5.3	84	703	6	MOLECULE: CELL DIVISION CYCLE PROTEIN 20 HOMOLOG;
932	3dl8-E	2.6	6.2	42	65	7	MOLECULE: PROTEIN TRANSLOCASE SUBUNIT SECA;
933	5hzd-A	2.6	5.3	84	476	10	MOLECULE: 3' TERMINAL URIDYLYL TRANSFERASE;
934	2f4q-A	2.6	6.3	61	309	5	MOLECULE: TYPE I TOPOISOMERASE, PUTATIVE;
935	6wql-A	2.6	4.5	44	49	7	MOLECULE: SEED PEPTIDE C2 (VBP-1);
936	5a5t-M	2.6	13.7	74	365	5	MOLECULE: EUKARYOTIC TRANSLATION INITIATION FACTOR 3 SUBUNI
937	6zui-A	2.6	12.1	70	420	3	MOLECULE: HTH-TYPE TRANSCRIPTIONAL REPRESSOR NEMR,GREEN FLU
938	6ds9-A	2.6	8.3	52	93	2	MOLECULE: DE NOVO DESIGNED THREE HELIX BUNDLE GRA3D;
939	6xl7-A	2.6	4.1	61	119	13	MOLECULE: SG7.AF;
940	3un9-A	2.6	9.9	76	294	8	MOLECULE: NLR FAMILY MEMBER X1;
941	3let-B	2.6	10.9	83	310	5	MOLECULE: ADENOSINE MONOPHOSPHATE-PROTEIN TRANSFERASE VOPS;
942	6thh-C	2.6	6.5	69	816	12	MOLECULE: SIRV2 ACRID1 (GP02) ANTI-CRISPR PROTEIN;

943	3hkl-B	2.6	5.5	56	153	4	MOLECULE: MUSCLE, SKELETAL RECEPTOR TYROSINE PROTEIN KINASE
944	3pla-A	2.6	10.5	68	375	7	MOLECULE: PRE MRNA SPLICING PROTEIN;
945	5td6-B	2.6	3.4	45	136	4	MOLECULE: FOG-3 PROTEIN;
946	6ka4-A	2.6	5.9	66	534	6	MOLECULE: F22L4.1 PROTEIN;
947	4m70-L	2.6	4	56	112	2	MOLECULE: RX PROTEIN;
948	7a6u-A	2.6	7.2	82	332	11	MOLECULE: SHORT TRANSIENT RECEPTOR POTENTIAL CHANNEL 6;
949	1zw0-D	2.6	4.7	38	66	5	MOLECULE: TYPE III SECRETION PROTEIN;
950	5xah-C	2.6	6.9	55	376	7	MOLECULE: IMPORTIN-4;
951	5kbc-A	2.6	5.5	63	195	5	MOLECULE: DSBA;
952	1gqe-A	2.6	7.9	83	362	11	MOLECULE: RELEASE FACTOR 2;
953	6cjd-A	2.6	10.8	58	128	14	MOLECULE: PUTATIVE CYTOPLASMIC PROTEIN;
954	5orf-A	2.6	10.6	87	586	3	MOLECULE: SERUM ALBUMIN;
955	2p7n-A	2.6	3.6	75	335	4	MOLECULE: PATHOGENICITY ISLAND 1 EFFECTOR PROTEIN;
956	6wqj-A	2.6	2.1	32	35	3	MOLECULE: VICILIN-BURIED PEPTIDE-10;
957	4dci-B	2.6	5.4	60	148	5	MOLECULE: UNCHARACTERIZED PROTEIN;
958	3lqi-C	2.6	3.6	66	181	12	MOLECULE: MLL1 PHD3-BROMO;
959	6n5x-A	2.6	8.5	57	168	7	MOLECULE: SORTING NEXIN-5,CATION-INDEPENDENT MANNOSE-6-PHOS
960	5mmj-c	2.6	7.9	51	216	6	MOLECULE: 50S RIBOSOMAL PROTEIN L31;
961	3jbr-F	2.6	9.9	71	872	7	MOLECULE: VOLTAGE-DEPENDENT L-TYPE CALCIUM CHANNEL SUBUNIT
962	5iy6-U	2.6	12.1	85	170	5	MOLECULE: DNA-DIRECTED RNA POLYMERASE II SUBUNIT RPB1;
963	7jw1-e	2.6	6.4	51	235	2	MOLECULE: CAPSID PROTEINS;
964	6ey4-A	2.6	8.6	92	483	10	MOLECULE: GLDM;
965	6wq2-a	2.6	8.2	63	202	8	MOLECULE: A-DNA;
966	2guz-A	2.6	5.7	47	71	13	MOLECULE: MITOCHONDRIAL IMPORT INNER MEMBRANE TRANSLOCASE S
967	6wej-A	2.6	14.9	76	513	4	MOLECULE: CYCLIC NUCLEOTIDE-GATED CATION CHANNEL;
968	1zbp-A	2.6	9.3	60	266	7	MOLECULE: HYPOTHETICAL PROTEIN VPA1032;
969	5xki-B	2.6	7	70	259	7	MOLECULE: FAD-LINKED SULFHYDRYL OXIDASE;
970	4qvr-A	2.6	4.5	65	318	11	MOLECULE: UNCHARACTERIZED HYPOTHETICAL PROTEIN FTT_1539C;
971	1g8p-A	2.6	4.3	71	321	8	MOLECULE: MAGNESIUM-CHELATASE 38 KDA SUBUNIT;
972	7cma-A	2.6	3.7	61	155	10	MOLECULE: A151R;
973	1z0j-B	2.6	3.8	40	51	3	MOLECULE: RAS-RELATED PROTEIN RAB-22A;
974	7emf-H	2.6	9.5	58	181	14	MOLECULE: MEDIATOR OF RNA POLYMERASE II TRANSCRIPTION SUBUN
975	2xco-A	2.6	14.4	66	636	8	MOLECULE: DNA GYRASE SUBUNIT B, DNA GYRASE SUBUNIT A;
976	6l85-B	2.6	9.9	64	401	3	MOLECULE: PHOSPHATE TRANSPORTER;
977	2h1n-A	2.6	13.6	75	566	9	MOLECULE: OLIGOENDOPEPTIDASE F;

978	6spb-Y	2.6	5.7	42	60	12	MOLECULE: 23S RIBOSOMAL RNA;
979	6kko-A	2.6	5.4	64	170	5	MOLECULE: PUTATIVE SERINE PHOSPHATASE;
980	4clv-A	2.6	8.5	70	142	6	MOLECULE: NICKEL-COBALT-CADMIUM RESISTANCE PROTEIN NCCX;
981	3a6m-A	2.6	6.5	50	168	6	MOLECULE: PROTEIN GRPE;
982	4an8-A	2.6	10.5	89	468	4	MOLECULE: CSE1;
983	3tsy-A	2.6	8.9	71	828	3	MOLECULE: FUSION PROTEIN 4-COUMARATE--COA LIGASE 1, RESVERA
984	4zi2-C	2.6	5.9	55	131	11	MOLECULE: ADP-RIBOSYLATION FACTOR-LIKE PROTEIN 3;
985	6ww2-R	2.6	4.3	63	425	3	MOLECULE: ANTI-BRIL FAB HEAVY CHAIN;
986	5w99-A	2.6	6.2	58	319	9	MOLECULE: PBTD;
987	1ghe-B	2.6	3.2	59	171	12	MOLECULE: ACETYLTRANSFERASE;
988	5ld2-D	2.6	8.4	66	605	3	MOLECULE: RECBCD ENZYME SUBUNIT RECB,RECBCD ENZYME SUBUNIT
989	6n8a-B	2.6	3.5	75	194	9	MOLECULE: TRANSCRIPTION REGULATOR ACAB;
990	2mqk-A	2.6	2.7	45	65	11	MOLECULE: ATP-DEPENDENT TARGET DNA ACTIVATOR B;
991	6ybv-s	2.6	4.1	66	138	8	MOLECULE: EUKARYOTIC TRANSLATION INITIATION FACTOR 2 SUBUNI
992	7apk-j	2.6	14.2	58	707	16	MOLECULE: THO COMPLEX SUBUNIT 1;
993	2nr5-E	2.6	4.4	43	65	12	MOLECULE: HYPOTHETICAL PROTEIN SO2669;
994	7etw-B	2.6	6.6	85	483	7	MOLECULE: INSULIN-INDUCED GENE 2 PROTEIN;
995	6lqz-A	2.6	3	49	71	10	MOLECULE: TRANSCRIPTION INITIATION FACTOR TFIID SUBUNIT 14;
996	3fd9-A	2.6	12.7	80	240	6	MOLECULE: UNCHARACTERIZED PROTEIN;
997	6njp-G	2.6	5.1	43	64	12	MOLECULE: TRANSLOCATOR ESCN;
998	6xxv-C	2.6	2.5	41	111	12	MOLECULE: ANTIBODY C57, HEAVY CHAIN;
999	6qum-N	2.6	8	70	649	7	MOLECULE: V-TYPE ATP SYNTHASE ALPHA CHAIN;
1000	3nl9-A	2.6	9.1	44	169	11	MOLECULE: PUTATIVE NTP PYROPHOSPHOHYDROLASE;
1001	4rkm-B	2.6	5.2	62	660	10	MOLECULE: MCCA;
1002	3etz-B	2.6	5.2	47	115	11	MOLECULE: ADHESIN A;
1003	2zoz-B	2.6	12.7	74	181	4	MOLECULE: TRANSCRIPTIONAL REGULATOR;
1004	5uac-C	2.6	9.8	65	1342	8	MOLECULE: DNA-DIRECTED RNA POLYMERASE SUBUNIT ALPHA;
1005	5w65-P	2.6	12.3	94	481	6	MOLECULE: DNA-DIRECTED RNA POLYMERASE I SUBUNIT RPA190;
1006	1au1-A	2.6	3.9	48	166	8	MOLECULE: INTERFERON-BETA;
1007	2mx9-B	2.6	4.5	71	133	3	MOLECULE: MINOR AMPULLATE SPIDROIN;
1008	7bin-V	2.6	4.6	48	133	13	MOLECULE: FLAGELLAR BIOSYNTHETIC PROTEIN FLIP;
1009	5n9j-G	2.6	6.9	54	163	15	MOLECULE: MEDIATOR OF RNA POLYMERASE II TRANSCRIPTION SUBUN
1010	5g4y-A	2.6	3.9	58	147	7	MOLECULE: CHEMOTAXIS PROTEIN;
1011	6rxd-A	2.6	10.1	76	509	5	MOLECULE: HISTIDINE ACID PHOSPHATASE;
1012	6okd-C	2.6	3.1	36	43	11	MOLECULE: TRANSFERRIN RECEPTOR PROTEIN 1;

1013	3otv-C	2.6	12.1	67	257	6	MOLECULE: PROBABLE CONSERVED TRANSMEMBRANE PROTEIN;
1014	1yz7-A	2.6	9	66	176	9	MOLECULE: PROBABLE TRANSLATION INITIATION FACTOR 2 ALPHA
1015	2d32-A	2.6	4.5	61	512	7	MOLECULE: GLUTAMATE--CYSTEINE LIGASE;
1016	5dgk-A	2.6	7.4	84	519	6	MOLECULE: ACTIVE HELICASE;
1017	7cbc-A	2.6	7.7	71	319	6	MOLECULE: DE NOVO DESIGNED SWITCH PROTEIN CAGING A HEMAGGLU
1018	3lph-A	2.6	4.1	42	62	17	MOLECULE: PROTEIN REV;
1019	5eqw-B	2.6	3.8	59	123	8	MOLECULE: PUTATIVE MAJOR COAT PROTEIN;
1020	5ul2-A	2.6	11.7	90	731	4	MOLECULE: OXSB PROTEIN;
1021	2vxo-B	2.6	11.9	57	658	7	MOLECULE: GMP SYNTHASE [GLUTAMINE-HYDROLYZING];
1022	6gv8-A	2.6	8.7	69	157	12	MOLECULE: HYPEROSMOLARITY RESISTANCE PROTEIN EMB;
1023	7ppo-A	2.6	6	76	500	12	MOLECULE: UBIQUITINATING/DEUBIQUITINATING ENZYME SDEA;
1024	5zk4-C	2.6	3.3	47	73	15	MOLECULE: DISD PROTEIN;
1025	2axt-Z	2.6	5.4	41	62	15	MOLECULE: PHOTOSYSTEM Q(B) PROTEIN;
1026	2mqa-A	2.6	3	65	125	8	MOLECULE: MINOR AMPULLATE FIBROIN 1;
1027	5zr1-B	2.6	11.3	68	374	4	MOLECULE: ORIGIN RECOGNITION COMPLEX SUBUNIT 1;
1028	7ey7-A	2.6	3.7	48	102	8	MOLECULE: TAIL FIBER PROTEIN;
1029	6qv7-A	2.6	12.6	72	316	8	MOLECULE: UNCHARACTERIZED PROTEIN;
1030	6g7c-C	2.6	12.7	79	239	11	MOLECULE: IMPA-RELATED DOMAIN PROTEIN;
1031	6ezn-H	2.6	8.8	54	259	7	MOLECULE: DOLICHYL-DIPHOSPHOOLIGOSACCHARIDE--PROTEIN
1032	4jza-A	2.6	14.6	104	817	8	MOLECULE: UNCHARACTERIZED PROTEIN;
1033	7nj0-C	2.6	4.6	66	270	3	MOLECULE: SECURIN,SEPARIN;
1034	3pyb-B	2.6	4.9	80	689	10	MOLECULE: ENT-COPALYL DIPHOSPHATE SYNTHASE, CHLOROPLASTIC;
1035	7lu4-A	2.6	12.1	63	978	6	MOLECULE: MULTIFUNCTIONAL FUSION PROTEIN;
1036	4q9u-A	2.6	3.8	62	303	3	MOLECULE: RAB5 GDP/GTP EXCHANGE FACTOR;
1037	7cxt-A	2.6	6.2	78	348	5	MOLECULE: GDP-L-FUCOSE SYNTHASE;
1038	5l1a-A	2.6	4.1	53	108	9	MOLECULE: UNCHARACTERIZED PROTEIN;
1039	5yti-A	2.6	18.3	69	330	9	MOLECULE: FLAGELLAR HOOK ASSOCIATED PROTEIN TYPE 3 FLGL;
1040	5yhf-A	2.6	11.3	102	734	6	MOLECULE: PROTEIN TRANSLOCASE SUBUNIT SECDF;
1041	6zls-A	2.6	5.1	61	292	5	MOLECULE: HISTIDINE KINASE;
1042	7ctq-A	2.6	12.5	72	429	7	MOLECULE: PEPTIDYL TRYPTOPHAN DIHYDROXYLASE;
1043	2qzg-B	2.6	3.1	56	90	13	MOLECULE: CONSERVED UNCHARACTERIZED ARCHAEAL PROTEIN;
1044	4l0r-A	2.6	5.3	49	74	6	MOLECULE: CENTROSOMAL PROTEIN OF 57 KDA;
1045	2rgo-A	2.6	5.4	64	557	11	MOLECULE: ALPHA-GLYCEROPHOSPHATE OXIDASE;
1046	7a23-J	2.6	10.6	55	96	2	MOLECULE: 51KDA;
1047	1abz-A	2.6	2.7	37	38	5	MOLECULE: ALPHA-T-ALPHA;

1048	4kyz-A	2.6	12.5	68	167	10	MOLECULE: DESIGNED PROTEIN OR327;
1049	4xng-D	2.6	10.2	65	144	6	MOLECULE: UNCHARACTERIZED PROTEIN MG218.1;
1050	6tpq-B	2.6	10.2	70	183	10	MOLECULE: RIBONUCLEASE M5;
1051	2keb-A	2.6	3.6	53	78	11	MOLECULE: DNA POLYMERASE SUBUNIT ALPHA B;
1052	3m1c-B	2.6	7.5	61	146	10	MOLECULE: ENVELOPE GLYCOPROTEIN H;
1053	2ve7-B	2.6	12.9	70	303	6	MOLECULE: KINETOCHORE PROTEIN HEC1, KINETOCHORE PROTEIN SPC
1054	6vbu-2	2.6	16.5	69	659	12	MOLECULE: BARDET-BIEDL SYNDROME 18 PROTEIN;
1055	4c3b-C	2.6	4.3	69	178	12	MOLECULE: MATRIX PROTEIN 2-1;
1056	3c4a-A	2.6	2.8	45	365	11	MOLECULE: PROBABLE TRYPTOPHAN HYDROXYLASE VIOD;
1057	2uwj-E	2.6	5.2	48	70	8	MOLECULE: TYPE III EXPORT PROTEIN PSCE;
1058	4nv0-B	2.6	11.9	73	300	4	MOLECULE: 7-METHYLGUANOSINE PHOSPHATE-SPECIFIC 5'-NUCLEOTID
1059	2cq8-A	2.6	2.8	54	110	13	MOLECULE: 10-FORMYLTETRAHYDROFOLATE DEHYDROGENASE;
1060	6psk-R	2.6	2.7	47	72	2	MOLECULE: ANTIHOLIN;
1061	3e7q-B	2.6	11	66	210	8	MOLECULE: TRANSCRIPTIONAL REGULATOR;
1062	1jud-A	2.6	18.6	71	220	11	MOLECULE: L-2-HALOACID DEHALOGENASE;
1063	6dkm-A	2.6	5.1	42	77	21	MOLECULE: DHD131_A;
1064	3me5-A	2.6	5.6	70	414	7	MOLECULE: CYTOSINE-SPECIFIC METHYLTRANSFERASE;
1065	6o3s-A	2.6	3.7	37	47	8	MOLECULE: RIBOSOME-INACTIVATING PROTEIN LUFFIN P1;
1066	1i49-A	2.6	8.3	60	201	8	MOLECULE: ARFAPTIN 2;
1067	4p6v-E	2.6	3.1	45	189	9	MOLECULE: NA(+)-TRANSLOCATING NADH-QUINONE REDUCTASE SUBUNI
1068	3ang-C	2.6	14.7	75	201	8	MOLECULE: TRANSCRIPTIONAL REPRESSOR, TETR FAMILY;
1069	2oo2-A	2.6	4.7	52	76	4	MOLECULE: HYPOTHETICAL PROTEIN AF_1782;
1070	3few-X	2.6	10.3	59	431	2	MOLECULE: COLICIN S4;
1071	5a60-A	2.6	10.1	104	429	12	MOLECULE: INORGANIC TRIPHOSPHATASE;
1072	3bk6-A	2.6	3.1	52	170	8	MOLECULE: PH STOMATIN;
1073	3wxx-B	2.6	7.5	78	183	10	MOLECULE: ACRH;
1074	3jd5-h	2.6	6.9	73	103	8	MOLECULE: 28S RIBOSOMAL RNA, MITOCHONDIAL;
1075	6cnb-R	2.6	10.7	71	522	11	MOLECULE: DNA-DIRECTED RNA POLYMERASE III SUBUNIT RPC1;
1076	6dxx-A	2.5	3.7	47	97	9	MOLECULE: N-ACYLETHANOLAMINE-HYDROLYZING ACID AMIDASE SUBUN
1077	2mx7-A	2.5	2.9	52	112	10	MOLECULE: SYNERGIN GAMMA;
1078	6awl-A	2.5	11.5	88	217	11	MOLECULE: UBIQUINONE BIOSYNTHESIS PROTEIN COQ9, MITOCHONDRI
1079	6qm7-K	2.5	6.2	66	206	5	MOLECULE: PROTEASOME ALPHA1 CHAIN;
1080	4uet-A	2.5	7.4	69	170	6	MOLECULE: NEMATODE FATTY ACID RETINOID BINDING PROTEIN;
1081	4cid-A	2.5	3.8	61	497	7	MOLECULE: EH DOMAIN-CONTAINING PROTEIN 2;
1082	6nd4-L	2.5	17.6	67	473	7	MOLECULE: ETS RRNA;

1083	3pwx-A	2.5	4.7	47	224	6	MOLECULE: PUTATIVE FLAGELLAR HOOK-ASSOCIATED PROTEIN;
1084	6dkm-D	2.5	4.6	42	79	0	MOLECULE: DHD131_A;
1085	1qoy-A	2.5	13.5	99	303	5	MOLECULE: HEMOLYSIN E;
1086	2n8p-A	2.5	3.3	44	53	16	MOLECULE: LACTICIN Q;
1087	6mj1-A	2.5	5	58	200	12	MOLECULE: PROBABLE HTH-TYPE TRANSCRIPTIONAL REGULATOR YTTP;
1088	3pux-G	2.5	9.1	70	293	10	MOLECULE: MALTOSE-BINDING PERIPLASMIC PROTEIN;
1089	4ebj-A	2.5	8.2	78	258	5	MOLECULE: AMINOGLYCOSIDE NUCLEOTIDYLTRANSFERASE;
1090	6q6e-A	2.5	6	77	221	8	MOLECULE: CONDENSIN COMPLEX SUBUNIT 2,STRUCTURAL MAINTENANC
1091	2p58-B	2.5	1.9	34	38	9	MOLECULE: PUTATIVE TYPE III SECRETION PROTEIN YSCE;
1092	2np9-A	2.5	5.3	59	423	8	MOLECULE: DPGC;
1093	6ofa-A	2.5	2.3	31	32	10	MOLECULE: WASABI RECEPTOR TOXIN;
1094	5ikn-A	2.5	9	87	639	6	MOLECULE: DNA-DIRECTED DNA POLYMERASE;
1095	1ng6-A	2.5	6.3	54	148	13	MOLECULE: HYPOTHETICAL PROTEIN YQEY;
1096	1w5s-B	2.5	6.2	65	396	6	MOLECULE: ORIGIN RECOGNITION COMPLEX SUBUNIT 2 ORC2;
1097	6g1f-A	2.5	11.8	77	443	6	MOLECULE: D-PHENYLGLYCINE AMINOTRANSFERASE;
1098	6x1g-A	2.5	12.6	57	216	4	MOLECULE: ULP_PROTEASE DOMAIN-CONTAINING PROTEIN;
1099	3oos-A	2.5	11.8	57	278	4	MOLECULE: ALPHA/BETA HYDROLASE FAMILY PROTEIN;
1100	6vtk-A	2.5	5.4	54	446	7	MOLECULE: ACID-SENSING ION CHANNEL 1;
1101	6tgb-B	2.5	10.9	69	680	9	MOLECULE: DEDICATOR OF CYTOKINESIS PROTEIN 2;
1102	5ys9-A	2.5	12.3	88	692	3	MOLECULE: ACYL-COENZYME A OXIDASE 3;
1103	3bos-B	2.5	3.7	50	231	16	MOLECULE: PUTATIVE DNA REPLICATION FACTOR;
1104	2ras-B	2.5	12.1	76	204	11	MOLECULE: TRANSCRIPTIONAL REGULATOR, TETR FAMILY;
1105	5csk-A	2.5	15.4	73	1996	11	MOLECULE: ACETYL-COA CARBOXYLASE;
1106	6ta5-D	2.5	10.2	56	345	5	MOLECULE: OUTER MEMBRANE PROTEIN OPRM;
1107	6ux5-A	2.5	2.9	37	50	5	MOLECULE: U-ACTITOXIN-AEQ5A;
1108	2oeq-C	2.5	5.2	49	117	10	MOLECULE: PROTEIN OF UNKNOWN FUNCTION, DUF964;
1109	1nbw-A	2.5	8	93	606	4	MOLECULE: GLYCEROL DEHYDRATASE REACTIVASE ALPHA SUBUNIT;
1110	4whj-A	2.5	6.4	79	565	9	MOLECULE: INTERFERON-INDUCED GTP-BINDING PROTEIN MX2;
1111	3may-C	2.5	3	52	97	4	MOLECULE: POSSIBLE EXPORTED PROTEIN;
1112	6zyx-Y	2.5	16.8	94	1067	10	MOLECULE: DYNEIN HEAVY CHAIN, OUTER ARM PROTEIN;
1113	3b5o-A	2.5	8.9	60	230	7	MOLECULE: CADD-LIKE PROTEIN OF UNKNOWN FUNCTION;
1114	3nuf-A	2.5	6.5	54	118	15	MOLECULE: PRD-CONTAINING TRANSCRIPTION REGULATOR;
1115	5npl-A	2.5	4.2	39	181	3	MOLECULE: SIMILAR TO TR|Q8YYT1|Q8YYT1;
1116	2pgc-A	2.5	14.8	55	206	4	MOLECULE: UNCHARACTERIZED PROTEIN;
1117	4fmt-A	2.5	8.3	59	209	10	MOLECULE: CHPT PROTEIN;

1118	6jbc-A	2.5	13.6	54	295	6	MOLECULE: PANTOATE KINASE;
1119	2gf4-A	2.5	5.7	58	89	9	MOLECULE: PROTEIN VNG1086C;
1120	6ts2-D	2.5	9.3	100	1123	5	MOLECULE: UDP-GLUCOSE-GLYCOPROTEIN GLUCOSYLTRANSFERASE-
1121	3stq-A	2.5	6.1	62	86	6	MOLECULE: PUTATIVE UNCHARACTERIZED PROTEIN;
1122	3enc-A	2.5	7.4	48	79	4	MOLECULE: PROTEIN PCC1;
1123	6sxf-A	2.5	12.1	70	263	3	MOLECULE: ION TRANSPORT PROTEIN;
1124	5gvx-A	2.5	7.5	66	386	11	MOLECULE: TREHALOSE-PHOSPHATE PHOSPHATASE;
1125	6pwy-B	2.5	4.8	89	513	8	MOLECULE: ZK177.8;
1126	6so5-E	2.5	3.5	45	103	4	MOLECULE: ATPASE ASNA1;
1127	6lk8-J	2.5	9.6	60	1026	2	MOLECULE: MGC83295 PROTEIN;
1128	5yma-A	2.5	7.4	57	179	7	MOLECULE: PUTATIVE RRNA PROCESSING PROTEIN;
1129	1kf6-D	2.5	9.1	54	119	7	MOLECULE: FUMARATE REDUCTASE FLAVOPROTEIN;
1130	5hc9-A	2.5	10.2	69	425	10	MOLECULE: TRNA NUCLEOTIDYL TRANSFERASE-RELATED PROTEIN;
1131	1gvn-A	2.5	6.7	51	87	4	MOLECULE: EPSILON;
1132	5ejk-D	2.5	7	72	250	11	MOLECULE: GAG-PRO-POL POLYPROTEIN;
1133	1ghh-A	2.5	2.5	39	81	10	MOLECULE: DNA-DAMAGE-INDUCIBLE PROTEIN I;
1134	7khw-a	2.5	6.8	56	174	11	MOLECULE: TRANSLOCON ESPA;
1135	7emf-G	2.5	14.4	67	161	3	MOLECULE: MEDIATOR OF RNA POLYMERASE II TRANSCRIPTION SUBUN
1136	6a70-B	2.5	11	52	704	4	MOLECULE: POLYCYSTIN-2;
1137	1ixs-A	2.5	3.2	42	50	19	MOLECULE: HOLLIDAY JUNCTION DNA HELICASE RUVA;
1138	2z51-A	2.5	3.5	53	154	9	MOLECULE: NIFU-LIKE PROTEIN 2, CHLOROPLAST;
1139	6c94-A	2.5	5.9	50	482	10	MOLECULE: CYTOCHROME P450 4B1;
1140	6c14-B	2.5	6	59	164	2	MOLECULE: PROTOCADHERIN-15;
1141	6pe4-A	2.5	7.7	73	758	8	MOLECULE: V-TYPE PROTON ATPASE SUBUNIT A, VACUOLAR ISOFORM;
1142	3cra-A	2.5	11.9	48	239	2	MOLECULE: PROTEIN MAZG;
1143	3gfi-A	2.5	5.9	51	143	6	MOLECULE: 146AA LONG HYPOTHETICAL TRANSCRIPTIONAL REGULATOR
1144	6vfi-A	2.5	4.4	75	197	8	MOLECULE: O43_DN18B;
1145	2ccy-A	2.5	5.3	43	127	7	MOLECULE: CYTOCHROME C;
1146	3ezu-A	2.5	7.5	76	336	5	MOLECULE: GGDEF DOMAIN PROTEIN;
1147	6blj-A	2.5	8	70	473	10	MOLECULE: SERINE-TRNA LIGASE;
1148	4cz8-A	2.5	12.8	75	422	15	MOLECULE: NA+/H+ ANTIPORTER, PUTATIVE;
1149	4y68-A	2.5	13.3	75	287	8	MOLECULE: PUTATIVE NISIN-RESISTANCE PROTEIN;
1150	2q83-A	2.5	6.5	69	332	3	MOLECULE: YTAA PROTEIN;
1151	2p11-A	2.5	10.2	70	219	7	MOLECULE: HYPOTHETICAL PROTEIN;
1152	5htf-B	2.5	16.1	80	260	6	MOLECULE: FOLDASE PROTEIN PRSA 1;

1153	4ghn-A	2.5	7.8	85	388	9	MOLECULE: UNCHARACTERIZED PROTEIN;
1154	3c98-B	2.5	11.4	88	230	10	MOLECULE: SYNTAXIN-BINDING PROTEIN 1;
1155	3sjb-D	2.5	6.6	51	72	10	MOLECULE: ATPASE GET3;
1156	4noo-B	2.5	4	49	95	8	MOLECULE: VGRG PROTEIN;
1157	3o0q-A	2.5	14	61	617	11	MOLECULE: RIBONUCLEOSIDE-DIPHOSPHATE REDUCTASE;
1158	5twa-A	2.5	10.4	47	161	11	MOLECULE: BCL-X HOMOLOGOUS PROTEIN, BHP2;
1159	1p49-A	2.5	5.6	55	549	4	MOLECULE: STERYL-SULFATASE;
1160	6tgb-A	2.5	17.4	90	1450	4	MOLECULE: DEDICATOR OF CYTOKINESIS PROTEIN 2;
1161	6y07-A	2.5	5.8	60	154	10	MOLECULE: SOHAIR;
1162	2gb7-B	2.5	6.4	68	295	9	MOLECULE: DNA STRAND 1;
1163	5dzr-A	2.5	12.4	67	965	9	MOLECULE: FRQ-INTERACTING RNA HELICASE;
1164	7nvr-n	2.5	8.5	58	132	16	MOLECULE: TFIIH BASAL TRANSCRIPTION FACTOR COMPLEX HELICASE
1165	3wvz-A	2.5	5.4	42	192	0	MOLECULE: PROTEIN HIKESHI;
1166	6sny-A	2.5	4.1	50	107	14	MOLECULE: SYNTHETIC EPCR BINDING PROTEIN;
1167	5z9o-A	2.5	7	70	373	9	MOLECULE: CYCLOPROPANE-FATTY-ACYL-PHOSPHOLIPID SYNTHASE;
1168	2ou3-A	2.5	8.7	57	160	2	MOLECULE: TELLURITE RESISTANCE PROTEIN OF COG3793;
1169	5zt3-A	2.5	3.5	51	114	8	MOLECULE: WA352;
1170	6gwj-B	2.5	3.6	47	84	11	MOLECULE: EKC/KEOPS COMPLEX SUBUNIT LAGE3;
1171	2g0u-A	2.5	6.5	62	92	6	MOLECULE: TYPE III SECRETION SYSTEM NEEDLE PROTEIN;
1172	5vj4-A	2.5	9.7	74	275	9	MOLECULE: UNCHARACTERIZED PROTEIN;
1173	3hr0-A	2.5	5.2	56	250	9	MOLECULE: COG4;
1174	3n71-A	2.5	15.5	91	469	11	MOLECULE: HISTONE LYSINE METHYLTRANSFERASE SMYD1;
1175	5tth-A	2.5	12.4	85	671	7	MOLECULE: C-TERMINAL SPYCATCHER FUSION OF WILDTYPE ZEBRAFIS
1176	7kzn-F	2.5	4.5	55	100	11	MOLECULE: HEAVY CHAIN ALPHA;
1177	5da9-A	2.5	6.5	63	433	5	MOLECULE: PUTATIVE UNCHARACTERIZED PROTEIN,PUTATIVE UNCHARA
1178	3dm0-A	2.5	10.7	64	675	3	MOLECULE: MALTOSE-BINDING PERIPLASMIC PROTEIN FUSED WITH RA
1179	6k0b-G	2.5	5.5	46	120	11	MOLECULE: RIBONUCLEASE P PROTEIN COMPONENT 2;
1180	6vld-B	2.5	5.4	52	470	6	MOLECULE: ALPHA-(1,6)-FUCOSYLTRANSFERASE;
1181	3b40-A	2.5	14.9	70	400	6	MOLECULE: PROBABLE DIPEPTIDASE;
1182	2id3-A	2.5	12.3	72	191	4	MOLECULE: PUTATIVE TRANSCRIPTIONAL REGULATOR;
1183	6so5-C	2.5	7.5	58	144	7	MOLECULE: ATPASE ASNA1;
1184	6jl7-A	2.5	10.1	88	444	8	MOLECULE: TBC1 DOMAIN FAMILY MEMBER 23;
1185	3wqy-A	2.5	12	71	906	11	MOLECULE: ALANINE--TRNA LIGASE;
1186	3r6n-A	2.5	10	80	450	5	MOLECULE: DESMOPLAKIN;
1187	5ot4-A	2.5	10.9	75	846	7	MOLECULE: INTERAPTIN;

1188	4zsf-A	2.5	10.4	72	272	8	MOLECULE: BSAWI ENDONUCLEASE;
1189	1ls4-A	2.5	4.4	76	164	7	MOLECULE: APOLIPOPHORIN-III;
1190	3zh9-B	2.5	11.8	76	339	17	MOLECULE: DELTA;
1191	7mi4-A	2.5	12.7	72	554	7	MOLECULE: CRISPR-ASSOCIATED EXONUCLEASE CAS4/ENDONUCLEASE C
1192	6h3a-B	2.5	2.5	39	57	8	MOLECULE: SWI/SNF-RELATED MATRIX-ASSOCIATED ACTIN-DEPENDENT
1193	6yj4-p	2.5	6.9	52	91	15	MOLECULE: NADH-UBIQUINONE OXIDOREDUCTASE CHAIN 3;
1194	5kbw-B	2.5	6.2	68	171	0	MOLECULE: RIBOFLAVIN TRANSPORTER RIBU;
1195	7rpk-A	2.5	7.5	103	953	8	MOLECULE: PROTEIN DISPATCHED HOMOLOG 1;
1196	4ua3-B	2.5	11.1	58	193	7	MOLECULE: UNCHARACTERIZED N-ACETYLTRANSFERASE C825.04C;
1197	3e98-A	2.5	4.5	71	178	6	MOLECULE: GAF DOMAIN OF UNKNOWN FUNCTION;
1198	2hkv-A	2.5	3.7	72	148	4	MOLECULE: HYPOTHETICAL PROTEIN;
1199	6zw0-A	2.5	17.1	65	299	11	MOLECULE: CONNECTASE MJ0548;
1200	5ikf-A	2.5	7.2	63	150	6	MOLECULE: CHROMATIN REMODELING FACTOR MIT1;
1201	6y86-A	2.5	5.7	75	425	8	MOLECULE: MEMBRANE PROTEIN INSERTASE YIDC;
1202	4am6-A	2.5	12.7	62	623	13	MOLECULE: ACTIN-LIKE PROTEIN ARP8;
1203	6wi5-A	2.5	3.8	55	90	11	MOLECULE: DE NOVO DESIGNED PROTEIN FOLDIT4;
1204	6zy2-E	2.5	5.6	67	259	4	MOLECULE: YRBD PROTEIN;
1205	6v8o-O	2.5	16.8	72	384	10	MOLECULE: HIGH TEMPERATURE LETHAL PROTEIN 1;
1206	6nqx-A	2.5	5.7	82	246	10	MOLECULE: FLAGELLAR COILING PROTEIN A;
1207	4q5i-B	2.5	16.6	80	269	6	MOLECULE: FERROUS IRON TRANSPORT PROTEIN B;
1208	5b2o-A	2.5	9.5	96	1455	9	MOLECULE: CRISPR-ASSOCIATED ENDONUCLEASE CAS9;
1209	3err-A	2.5	7.8	62	527	10	MOLECULE: FUSION PROTEIN OF MICROTUBULE BINDING DOMAIN FROM
1210	4bpm-A	2.5	2.9	51	162	14	MOLECULE: PROSTAGLANDIN E SYNTHASE, FUSION PEPTIDE;
1211	5fyw-M	2.5	11	67	231	4	MOLECULE: DNA-DIRECTED RNA POLYMERASE II SUBUNIT RPB1;
1212	6yj4-V	2.5	7.1	49	126	6	MOLECULE: NADH-UBIQUINONE OXIDOREDUCTASE CHAIN 3;
1213	5gj8-D	2.5	4.6	60	121	3	MOLECULE: ACYL-COA DEHYDROGENASE TYPE 2 DOMAIN PROTEIN;
1214	6yxa-A	2.5	7.9	61	536	10	MOLECULE: GTP PYROPHOSPHOKINASE;
1215	6j98-A	2.5	2.3	48	75	6	MOLECULE: P8;
1216	7lgu-A	2.5	19.6	82	680	7	MOLECULE: PRESTIN;
1217	5w66-M	2.5	7.7	72	396	8	MOLECULE: DNA-DIRECTED RNA POLYMERASE I SUBUNIT RPA190;
1218	7b1s-B	2.5	5.8	82	466	6	MOLECULE: ETHYL-COENZYME M REDUCTASE ALPHA SUBUNIT;
1219	6vjy-B	2.5	11	97	280	5	MOLECULE: ERAD-ASSOCIATED E3 UBIQUITIN-PROTEIN LIGASE HRD1;
1220	5x6b-J	2.5	8.3	74	377	11	MOLECULE: O-PHOSPHO-L-SERYL-TRNA:CYS-TRNA SYNTHASE;
1221	5ee5-A	2.5	8.2	63	202	8	MOLECULE: BREFELDIN A-INHIBITED GUANINE NUCLEOTIDE-EXCHANGE
1222	7jv7-B	2.5	5.3	81	310	6	MOLECULE: CTD KINASE SUBUNIT ALPHA;

1223	4avm-A	2.5	9.5	78	230	8	MOLECULE: BRIDGING INTEGRATOR 2;
1224	7mi1-A	2.5	11.4	100	2628	5	MOLECULE: CHIMERA PROTEIN OF DYNEIN AND ENDOLYSIN;
1225	1rso-B	2.5	4.9	44	56	16	MOLECULE: PRESYNAPTIC PROTEIN SAP97;
1226	7jts-s	2.5	5.7	58	290	12	MOLECULE: RADIAL SPOKE PROTEIN 3;
1227	5gad-i	2.5	6.6	73	450	8	MOLECULE: ESRP 4.5S RNA;
1228	6k6l-B	2.5	6.6	73	263	8	MOLECULE: PSEUDO DEUBIQUITINASE;
1229	4nb5-B	2.5	4.8	49	149	4	MOLECULE: DNA BINDING PROTEIN;
1230	5sva-V	2.5	6.9	57	85	11	MOLECULE: DNA-DIRECTED RNA POLYMERASE II SUBUNIT RPB1;
1231	2y1v-A	2.5	8.9	73	604	7	MOLECULE: CELL WALL SURFACE ANCHOR FAMILY PROTEIN;
1232	3s84-B	2.5	9.8	78	241	6	MOLECULE: APOLIPOPROTEIN A-IV;
1233	6w2r-B	2.5	10	65	221	9	MOLECULE: JUNCTION 19 DHR54-DHR79;
1234	1qdb-A	2.5	19.3	92	473	10	MOLECULE: CYTOCHROME C NITRITE REDUCTASE;
1235	5yud-C	2.5	4.8	49	75	12	MOLECULE: BACULOVIRAL IAP REPEAT-CONTAINING PROTEIN 1E;
1236	6ty9-B	2.5	8.8	55	501	4	MOLECULE: RNA-DEPENDENT RNA POLYMERASE;
1237	6qyi-B	2.5	11	81	519	5	MOLECULE: 4-HYDROXYPHENYLACETATE 3-MONOOXYGENASE
1238	7nyx-A	2.5	7.7	65	1467	8	MOLECULE: CHROMOSOME PARTITION PROTEIN MUKB;
1239	1jeq-A	2.5	16.9	72	548	10	MOLECULE: KU70;
1240	3bvo-A	2.5	9.2	65	197	6	MOLECULE: CO-CHAPERONE PROTEIN HSCB, MITOCHONDRIAL PRECURSO
1241	5yz0-A	2.5	13.3	84	2362	8	MOLECULE: SERINE/THREONINE-PROTEIN KINASE ATR;
1242	6cc4-A	2.5	4.9	67	608	4	MOLECULE: SOLUBLE CYTOCHROME B562, LIPID II FLIPPASE MURJ C
1243	7cpx-A	2.5	4.8	87	2262	8	MOLECULE: LOVASTATIN NONAKETIDE SYNTHASE, POLYKETIDE SYNTHA
1244	3n27-B	2.5	4.6	43	79	5	MOLECULE: FUSION GLYCOPROTEIN F0, LINKER, FUSION GLYCOPROTE
1245	4cej-A	2.5	7.6	65	1177	5	MOLECULE: ATP-DEPENDENT HELICASE/NUCLEASE SUBUNIT A;
1246	6v69-J	2.5	7.5	81	484	10	MOLECULE: GAMMA-TUBULIN COMPLEX COMPONENT 5;
1247	7cfa-A	2.5	5.9	54	234	9	MOLECULE: R.PAB1 FAMILY RESTRICTION ENDONUCLEASE;
1248	3di5-A	2.5	3.8	76	150	5	MOLECULE: DINB-LIKE PROTEIN;
1249	6tdx-O	2.5	4.1	50	81	8	MOLECULE: ATP SYNTHASE F1 SUBUNIT GAMMA;
1250	7emf-P	2.5	7.9	78	766	12	MOLECULE: MEDIATOR OF RNA POLYMERASE II TRANSCRIPTION SUBUN
1251	3ph0-A	2.5	5	46	60	7	MOLECULE: ASCE;
1252	6s7j-A	2.5	10.8	110	497	8	MOLECULE: UNCHARACTERIZED PROTEIN;
1253	2is5-C	2.5	3.3	55	134	4	MOLECULE: HYPOTHETICAL PROTEIN;
1254	5a63-C	2.5	11.4	84	243	6	MOLECULE: NICASTRIN;
1255	6dlm-B	2.5	5	48	72	6	MOLECULE: DHD127_A;
1256	4uzx-A	2.5	4.1	41	67	27	MOLECULE: PROTEIN THO1;
1257	6vq6-A	2.5	9.1	82	600	5	MOLECULE: ATPASE H+-TRANSPORTING V1 SUBUNIT A;

1258	7lkm-A	2.4	2.9	58	149	2	MOLECULE: PILUS BIOGENESIS PROTEIN;
1259	4d2i-A	2.4	22.8	93	464	9	MOLECULE: HERA;
1260	5lde-B	2.4	2.9	64	225	8	MOLECULE: IMMUNOGLOBULIN G-BINDING PROTEIN G,VIRAL FLICE PR
1261	4zhe-D	2.4	10.8	86	206	7	MOLECULE: ASPR2 PROTEIN;
1262	3n0r-A	2.4	11.4	69	258	3	MOLECULE: RESPONSE REGULATOR;
1263	6sp2-A	2.4	7.6	83	366	8	MOLECULE: MEMBRANE PROTEIN TMS1D;
1264	5wd8-A	2.4	5.3	44	90	7	MOLECULE: UNCHARACTERIZED PROTEIN;
1265	5lxj-A	2.4	2.9	44	53	5	MOLECULE: PHOSPHOPROTEIN;
1266	6zka-s	2.4	7.3	57	122	5	MOLECULE: NADH-UBIQUINONE OXIDOREDUCTASE CHAIN 3;
1267	1x2g-C	2.4	2.3	46	337	7	MOLECULE: LIPOATE-PROTEIN LIGASE A;
1268	1x04-A	2.4	7.7	73	200	10	MOLECULE: SH3-CONTAINING GRB2-LIKE PROTEIN 2;
1269	7kyp-F	2.4	5.4	65	290	5	MOLECULE: MANGANESE ABC TRANSPORTER, ATP-BINDING PROTEIN;
1270	6gyr-A	2.4	14.6	79	589	9	MOLECULE: HISTONE ACETYLTRANSFERASE P300;
1271	2x43-S	2.4	4.4	48	67	8	MOLECULE: SHERP;
1272	2rji-A	2.4	2.1	41	84	15	MOLECULE: ERYTHROCYTE BINDING ANTIGEN 175;
1273	1gjs-A	2.4	2.6	41	65	7	MOLECULE: IMMUNOGLOBULIN G BINDING PROTEIN G;
1274	2k3q-A	2.4	4.1	64	118	11	MOLECULE: TUSP1;
1275	6tmh-H	2.4	3.9	42	71	7	MOLECULE: INHIBITOR OF F1;
1276	5m1m-A	2.4	5	47	154	4	MOLECULE: MATRIX PROTEIN 1;
1277	2zc2-A	2.4	2.9	46	75	9	MOLECULE: DNAD-LIKE REPLICATION PROTEIN;
1278	2dhy-A	2.4	3.4	45	67	9	MOLECULE: CUE DOMAIN-CONTAINING PROTEIN 1;
1279	5hsb-A	2.4	8.6	46	202	9	MOLECULE: RNA POLYMERASE;
1280	6nsm-A	2.4	10.4	63	194	3	MOLECULE: COPPER OUTER MEMBRANE REGULATOR;
1281	4i0x-F	2.4	4.3	44	85	9	MOLECULE: ESAT-6-LIKE PROTEIN MAB_3112;
1282	4oge-A	2.4	12.7	84	977	7	MOLECULE: HNH ENDONUCLEASE DOMAIN PROTEIN;
1283	3uo3-B	2.4	6.6	53	147	9	MOLECULE: J-TYPE CO-CHAPERONE JAC1, MITOCHONDRIAL;
1284	2yev-C	2.4	4.7	47	64	11	MOLECULE: CYTOCHROME C OXIDASE POLYPEPTIDE I+III;
1285	6eqo-A	2.4	8.8	86	1804	9	MOLECULE: ACETYL-COENZYME A SYNTHETASE;
1286	3gnw-B	2.4	10.6	83	568	8	MOLECULE: RNA-DIRECTED RNA POLYMERASE;
1287	5gar-O	2.4	4.8	44	79	7	MOLECULE: V-TYPE ATP SYNTHASE ALPHA CHAIN;
1288	6qbi-A	2.4	11.7	48	102	8	MOLECULE: SURFACE PROTEIN, MLP LIPOPROTEIN FAMILY;
1289	3aai-A	2.4	8.2	57	78	14	MOLECULE: COPPER HOMEOSTASIS OPERON REGULATORY PROTEIN;
1290	6row-B	2.4	9.3	77	619	8	MOLECULE: PUTATIVE ZINC METALLOPEPTIDASE;
1291	6ffv-A	2.4	8.4	76	194	9	MOLECULE: BTUM;
1292	6e67-B	2.4	4.2	59	476	7	MOLECULE: BETA-2 ADRENERGIC RECEPTOR,ENDOLYSIN,GUANINE NUCL

1293	2oh3-A	2.4	6.3	63	149	10	MOLECULE: COG1633: UNCHARACTERIZED CONSERVED PROTEIN;
1294	2pjw-H	2.4	8.3	62	88	3	MOLECULE: UNCHARACTERIZED PROTEIN YHL002W;
1295	3b4q-B	2.4	2.7	48	90	10	MOLECULE: UNCHARACTERIZED PROTEIN;
1296	6n10-A	2.4	11.3	52	403	10	MOLECULE: DIPHOSPHOMEVALONATE DECARBOXYLASE MVD1,
1297	6h4b-B	2.4	8	48	84	6	MOLECULE: ORF026;
1298	5oqj-W	2.4	13	62	258	6	MOLECULE: DNA-DIRECTED RNA POLYMERASE II SUBUNIT RPB1;
1299	6p8r-B	2.4	9.8	55	168	9	MOLECULE: HORMA DOMAIN CONTAINING PROTEIN;
1300	2p1a-B	2.4	3.2	53	150	9	MOLECULE: HYPOTHETICAL PROTEIN;
1301	7ol3-B	2.4	3.2	42	425	14	MOLECULE: ATLASTIN-1;
1302	5l10-A	2.4	8.7	53	172	4	MOLECULE: N-ACYLHOMOSERINE LACTONE DEPENDENT REGULATORY
1303	6rd4-R	2.4	8.2	53	177	6	MOLECULE: ASA-10: POLYTOMELLA F-ATP SYNTHASE ASSOCIATED SUB
1304	6l81-A	2.4	2.7	49	96	14	MOLECULE: GAMMA-TUBULIN COMPLEX COMPONENT 5;
1305	7rj1-A	2.4	11.1	77	266	4	MOLECULE: CHORISMATE MUTASE;
1306	5tue-A	2.4	6.9	62	388	8	MOLECULE: TETRACYCLINE DESTRUCTASE TET(50);
1307	6snh-X	2.4	3.4	60	479	2	MOLECULE: DOLICHYL PYROPHOSPHATE MAN9GLCNAC2 ALPHA-1,3-
1308	7d7q-B	2.4	4.4	78	310	10	MOLECULE: PHOSPHODIESTERASE;
1309	2r01-A	2.4	5.8	70	195	6	MOLECULE: NITROREDUCTASE FAMILY PROTEIN;
1310	1tua-A	2.4	12	59	189	7	MOLECULE: HYPOTHETICAL PROTEIN APE0754;
1311	5oxe-A	2.4	4.9	45	70	4	MOLECULE: MAJOR VIRION PROTEIN;
1312	6rwb-A	2.4	6.3	92	1873	5	MOLECULE: TOXIN,TOXIN COMPLEX SUBUNIT TCAB,PUTATIVE TOXIN S
1313	6xm1-D	2.4	9.7	74	181	9	MOLECULE: VPS45;
1314	5xmk-G	2.4	4.6	38	54	5	MOLECULE: VACUOLAR PROTEIN SORTING-ASSOCIATED PROTEIN 4;
1315	6vk0-C	2.4	7.7	68	180	7	MOLECULE: U1 SNP1-ASSOCIATING PROTEIN 1;
1316	6yeu-A	2.4	3.1	58	107	9	MOLECULE: CALCIUM-BINDING EF HAND FAMILY PROTEIN;
1317	6ysf-F	2.4	10	74	257	1	MOLECULE: CHEMOTAXIS MOTB PROTEIN;
1318	6y1y-B	2.4	6.1	56	128	4	MOLECULE: CHEA;
1319	6jho-A	2.4	5.6	55	200	4	MOLECULE: CAG PATHOGENICITY ISLAND PROTEIN (CAG6);
1320	1vku-A	2.4	2.4	49	85	10	MOLECULE: ACYL CARRIER PROTEIN;
1321	7nhr-A	2.4	7.3	75	663	7	MOLECULE: PUTATIVE TRANSMEMBRANE PROTEIN WZC;
1322	1w8i-A	2.4	3.1	44	155	2	MOLECULE: PUTATIVE VAPC RIBONUCLEASE AF_1683;
1323	2iie-A	2.4	6.2	53	204	15	MOLECULE: PHAGE P H' SITE;
1324	5mmi-Z	2.4	6.7	58	101	9	MOLECULE: 50S RIBOSOMAL PROTEIN L31;
1325	6en8-A	2.4	3.2	47	191	13	MOLECULE: TRANSCRIPTIONAL REGULATOR TETR FAMILY;
1326	6yp7-z	2.4	5.5	41	62	12	MOLECULE: CHLOROPHYLL A-B BINDING PROTEIN 8, CHLOROPLASTIC;
1327	2mmu-A	2.4	4.4	40	50	5	MOLECULE: CELL DIVISION PROTEIN CRGA;

1328	2ymj-A	2.4	7.2	39	52	8	MOLECULE: PROTEIN QUAKING-A;
1329	2qko-B	2.4	13.1	75	176	7	MOLECULE: POSSIBLE TRANSCRIPTIONAL REGULATOR, TETR FAMILY P
1330	6m6z-A	2.4	7	67	203	6	MOLECULE: TMH4C4;
1331	2ixp-B	2.4	4.2	66	316	8	MOLECULE: SERINE/THREONINE-PROTEIN PHOSPHATASE 2A ACTIVATOR
1332	6g7o-A	2.4	12.8	55	350	4	MOLECULE: ALKALINE CERAMIDASE 3,SOLUBLE CYTOCHROME B562;
1333	5yo8-A	2.4	6.6	66	349	6	MOLECULE: TETRAPRENYL-BETA-CURCUMENE SYNTHASE;
1334	3fx7-B	2.4	6.5	52	87	6	MOLECULE: PUTATIVE UNCHARACTERIZED PROTEIN;
1335	3di3-A	2.4	5.4	46	118	13	MOLECULE: INTERLEUKIN-7;
1336	4kzs-A	2.4	9.6	58	242	12	MOLECULE: LPP20 LIPOFAMILY PROTEIN;
1337	4mou-A	2.4	11.7	63	261	5	MOLECULE: ENOYL-COA HYDRATASE/ISOMERASE FAMILY PROTEIN;
1338	4gmq-A	2.4	2.7	54	92	15	MOLECULE: PUTATIVE RIBOSOME ASSOCIATED PROTEIN;
1339	5ly0-A	2.4	6.4	66	108	3	MOLECULE: LOB FAMILY TRANSFACTOR RAMOSA2.1;
1340	5c37-A	2.4	8	66	632	14	MOLECULE: FATTY ACID SYNTHASE;
1341	2g7s-A	2.4	11.2	72	190	6	MOLECULE: TRANSCRIPTIONAL REGULATOR, TETR FAMILY;
1342	5gm2-B	2.4	13	72	283	7	MOLECULE: O-METHYLRANSFERASE;
1343	3i5q-A	2.4	4.4	45	248	16	MOLECULE: NUCLEOPORIN NUP170;
1344	2zdi-C	2.4	5.7	49	148	6	MOLECULE: PREFOLDIN SUBUNIT BETA;
1345	6dtd-A	2.4	4.3	77	1053	6	MOLECULE: NUCLEASE;
1346	6pl5-A	2.4	8.5	89	336	15	MOLECULE: PEPTIDOGLYCAN GLYCOSYLTRANSFERASE RODA;
1347	7lg5-A	2.4	5.4	64	871	9	MOLECULE: CYANOPHYCIN SYNTHASE;
1348	2byd-A	2.4	5.6	77	283	6	MOLECULE: HSPC223;
1349	5zr1-E	2.4	9.6	71	460	7	MOLECULE: ORIGIN RECOGNITION COMPLEX SUBUNIT 1;
1350	2job-A	2.4	2.9	54	102	9	MOLECULE: ANTILIPOPOLYSACCHARIDE FACTOR;
1351	7asm-W	2.4	4.3	43	66	14	MOLECULE: 50S RIBOSOMAL PROTEIN L19;
1352	3e7g-C	2.4	12.7	89	423	2	MOLECULE: NITRIC OXIDE SYNTHASE, INDUCIBLE;
1353	5a1u-E	2.4	17.7	75	822	9	MOLECULE: ADP-RIBOSYLATION FACTOR 1;
1354	6eml-p	2.4	3	48	185	6	MOLECULE: PRE-18S RIBOSOMAL RNA;
1355	3hh0-A	2.4	2	34	134	18	MOLECULE: TRANSCRIPTIONAL REGULATOR, MERR FAMILY;
1356	7jfs-A	2.4	13	74	958	8	MOLECULE: F5/8 TYPE C DOMAIN PROTEIN;
1357	6eud-A	2.4	14.8	87	808	11	MOLECULE: ATP-DEPENDENT RNA HELICASE HRPB;
1358	5jno-A	2.4	2.9	52	97	4	MOLECULE: BEN DOMAIN-CONTAINING PROTEIN 3;
1359	6ck0-B	2.4	4.3	66	210	3	MOLECULE: BIOTIN ACETYL COENZYME A CARBOXYLASE SYNTHETASE;
1360	6ejq-B	2.4	9.7	51	141	8	MOLECULE: TERMINASE SMALL SUBUNIT;
1361	2zop-A	2.4	3.6	51	113	12	MOLECULE: PUTATIVE UNCHARACTERIZED PROTEIN TTHB164;
1362	6zr2-Y	2.4	7.4	69	140	10	MOLECULE: NADH-UBIQUINONE OXIDOREDUCTASE CHAIN 3;

1363	3c2b-A	2.4	2.7	48	200	13	MOLECULE: TRANSCRIPTIONAL REGULATOR, TETR FAMILY;
1364	4y2f-A	2.4	3.7	43	143	12	MOLECULE: SENSOR PROTEIN KDPD;
1365	3v9r-A	2.4	11	62	88	2	MOLECULE: UNCHARACTERIZED PROTEIN YOL086W-A;
1366	5i2l-A	2.4	2.9	44	105	5	MOLECULE: EF-HAND DOMAIN-CONTAINING PROTEIN D2;
1367	2ch5-A	2.4	8.3	90	344	7	MOLECULE: NAGK PROTEIN;
1368	6xmv-A	2.4	3.5	61	307	8	MOLECULE: HEMOLYSIN,ENDOLYSIN;
1369	5lut-F	2.4	3.6	48	55	6	MOLECULE: BLM HELICASE;
1370	6ywd-C	2.4	8.2	45	75	7	MOLECULE: ANTIBODY MOTA, HEAVY CHAIN;
1371	6vy1-A	2.4	4.7	43	121	26	MOLECULE: PREFOLDIN SUBUNIT ALPHA 2;
1372	5nh2-B	2.4	3.3	38	56	8	MOLECULE: ADENOSINE MONOPHOSPHATE-PROTEIN TRANSFERASE;
1373	5z62-B	2.4	9.7	64	227	9	MOLECULE: CYTOCHROME C OXIDASE SUBUNIT 1;
1374	2i2x-B	2.4	8.5	73	258	7	MOLECULE: METHYLTRANSFERASE 1;
1375	3v7o-B	2.4	3.5	65	203	14	MOLECULE: MINOR NUCLEOPROTEIN VP30;
1376	5dic-A	2.4	4.1	50	115	4	MOLECULE: ODORANT-BINDING PROTEIN;
1377	5ndd-A	2.4	6.1	69	567	7	MOLECULE: LYSOZYME,PROTEINASE-ACTIVATED RECEPTOR 2,SOLUBLE
1378	1wpk-A	2.4	10.2	69	146	6	MOLECULE: ADA REGULATORY PROTEIN;
1379	5hus-A	2.4	12.3	61	293	7	MOLECULE: TREHALOSE SYNTHASE REGULATORY PROTEIN;
1380	2o57-A	2.4	6.5	69	282	7	MOLECULE: PUTATIVE SARCOSINE DIMETHYLGLYCINE METHYLTRANSFER
1381	4jcs-A	2.4	9.3	61	265	3	MOLECULE: ENOYL-COA HYDRATASE/ISOMERASE;
1382	2y31-A	2.4	12.5	67	242	4	MOLECULE: PUTATIVE REPRESSOR SIMREG2;
1383	6qi8-E	2.4	8.4	67	335	13	MOLECULE: RUVB-LIKE 1;
1384	6nnw-A	2.4	5.7	50	208	12	MOLECULE: TETRONASIN;
1385	4u67-P	2.4	7	63	130	6	MOLECULE: 50S RIBOSOMAL PROTEIN L2;
1386	5kd2-A	2.4	13.9	89	591	7	MOLECULE: METALLOPEPTIDASE;
1387	6p8v-C	2.4	9.6	53	303	8	MOLECULE: ATPASE, AAA FAMILY;
1388	2fna-A	2.4	12.7	67	352	7	MOLECULE: CONSERVED HYPOTHETICAL PROTEIN;
1389	6gef-A	2.4	3.9	63	388	0	MOLECULE: TYPE IV SECRETION SYSTEM PROTEIN DOTB;
1390	5t58-N	2.4	3.8	54	196	6	MOLECULE: KLLA0F02343P;
1391	3cqx-D	2.4	4.6	39	84	3	MOLECULE: HEAT SHOCK COGNATE 71 KDA PROTEIN;
1392	6wq0-A	2.4	9.4	56	131	4	MOLECULE: DNA (301-MER);
1393	6zie-A	2.4	4.6	53	123	9	MOLECULE: CMPX-383B;
1394	3mka-J	2.4	8.3	75	252	5	MOLECULE: PROTEASOME SUBUNIT ALPHA;
1395	4y9j-B	2.4	8.7	81	593	9	MOLECULE: PROTEIN ACDH-11, ISOFORM B;
1396	4ip8-A	2.4	5.6	52	105	4	MOLECULE: SERUM AMYLOID A-1 PROTEIN;
1397	6scj-A	2.4	7.1	89	2551	8	MOLECULE: THYROGLOBULIN;

1398	4fz4-A	2.4	9	76	154	13	MOLECULE: UNCHARACTERIZED PROTEIN CONSERVED IN BACTERIA;
1399	1e1d-A	2.4	10.2	78	553	8	MOLECULE: HYDROXYLAMINE REDUCTASE;
1400	3s1e-A	2.4	14.1	74	499	8	MOLECULE: CYTOKININ DEHYDROGENASE 1;
1401	6o3v-B	2.4	5.9	79	831	8	MOLECULE: PROTEIN VP3;
1402	6tnn-I	2.4	3.9	73	133	5	MOLECULE: 50S RIBOSOMAL PROTEIN L10;
1403	4abx-A	2.4	4.1	55	167	7	MOLECULE: DNA REPAIR PROTEIN RECN;
1404	6ewz-A	2.4	11.4	66	202	8	MOLECULE: GTP PYROPHOSPHOKINASE;
1405	1xbn-A	2.4	3.9	71	195	8	MOLECULE: METHYL-ACCEPTING CHEMOTAXIS PROTEIN;
1406	2uui-A	2.4	9.2	60	155	2	MOLECULE: LEUKOTRIENE C4 SYNTHASE;
1407	6edw-B	2.4	14.5	81	746	7	MOLECULE: ISOCITRATE LYASE 2;
1408	5me8-A	2.4	6.2	53	107	9	MOLECULE: INHIBITOR OF GROWTH PROTEIN 5;
1409	7ahd-A	2.4	6.4	71	547	8	MOLECULE: ABC-TYPE PROLINE/GLYCINE BETAINE TRANSPORT SYSTEM
1410	5dku-B	2.4	12	73	581	8	MOLECULE: PREX DNA POLYMERASE;
1411	3d1b-A	2.4	3.6	49	111	8	MOLECULE: RNA-INDUCED TRANSCRIPTIONAL SILENCING COMPLEX PRO
1412	6sku-A	2.4	14.4	82	785	10	MOLECULE: PHOSPHOCHOLINE TRANSFERASE ANKX;
1413	3eps-A	2.4	14.3	94	566	6	MOLECULE: ISOCITRATE DEHYDROGENASE KINASE/PHOSPHATASE;
1414	5hwy-A	2.4	4.2	63	300	5	MOLECULE: UNCHARACTERIZED MEMBRANE PROTEIN MJ0091;
1415	6al9-B	2.4	6	68	91	7	MOLECULE: CHORISMATE MUTASE;
1416	4j41-B	2.4	5.3	53	86	4	MOLECULE: SECRETED PROTEIN ESXB;
1417	2ajq-A	2.4	6.7	58	704	9	MOLECULE: DNA PRIMER;
1418	6ty9-A	2.4	13.2	99	1208	5	MOLECULE: RNA-DEPENDENT RNA POLYMERASE;
1419	2au5-A	2.4	3.7	67	129	10	MOLECULE: CONSERVED DOMAIN PROTEIN;
1420	4f0x-A	2.4	6.4	74	456	3	MOLECULE: MALONYL-COA DECARBOXYLASE, MITOCHONDRIAL;
1421	5cr4-B	2.4	8.5	63	226	10	MOLECULE: SLEEPING BEAUTY TRANSPOSASE, SB100X;
1422	4c00-A	2.4	15.3	67	544	12	MOLECULE: TRANSLOCATION AND ASSEMBLY MODULE TAMA;
1423	5mqf-M	2.4	4.7	60	706	5	MOLECULE: PRE-MRNA-PROCESSING-SPLICING FACTOR 8;
1424	4hga-A	2.4	8.1	48	207	15	MOLECULE: DEATH DOMAIN-ASSOCIATED PROTEIN 6;
1425	5mpd-Z	2.4	15	89	906	2	MOLECULE: 26S PROTEASOME REGULATORY SUBUNIT RPN10;
1426	2raj-A	2.4	5.9	91	382	7	MOLECULE: SORTING NEXIN-9;
1427	6zsi-D	2.4	4.7	45	133	9	MOLECULE: RAS-RELATED PROTEIN RAB-8A;
1428	5ecj-A	2.4	11.1	55	263	4	MOLECULE: PR DOMAIN ZINC FINGER PROTEIN 14,PROTEIN CBFA2T2;
1429	7bgy-C	2.4	3.6	45	190	11	MOLECULE: POTASSIUM-TRANSPORTING ATPASE POTASSIUM-BINDING S
1430	6s1k-A	2.4	13	58	383	7	MOLECULE: CHEMOTAXIS PROTEIN CHEA;
1431	2fm8-C	2.4	14.1	58	220	10	MOLECULE: SURFACE PRESENTATION OF ANTIGENS PROTEIN SPAK;
1432	5xu0-B	2.4	5	58	231	5	MOLECULE: MEMBRANE-FUSION PROTEIN;

1433	3boy-A	2.4	5.1	66	150	8	MOLECULE: 5'-R(*UP*UP*UP*AP*GP*UP*UP*UP*UP*UP*AP*GP*UP*UP*U
1434	7krw-A	2.4	12.3	73	608	10	MOLECULE: CHAPERONE PROTEIN DNAK FUSED WITH SUBSTRATE PEPTI
1435	3u24-A	2.4	10.3	80	546	5	MOLECULE: PUTATIVE LIPOPROTEIN;
1436	4qes-A	2.4	4.6	61	441	5	MOLECULE: NON-HAEM BROMOPEROXIDASE BPO-A2, MATRIX PROTEIN 1
1437	7k5c-A	2.4	12.8	88	650	10	MOLECULE: INTERNAL VIRION PROTEIN GP15;
1438	6zyv-A	2.4	7.1	57	238	9	MOLECULE: CIR PROTEIN;
1439	4gl6-B	2.4	3.8	48	241	10	MOLECULE: HYPOTHETICAL PROTEIN;
1440	5lnk-M	2.4	9.7	83	459	14	MOLECULE: MITOCHONDRIAL COMPLEX I, 51 KDA SUBUNIT;
1441	2ex3-B	2.4	11.3	50	196	8	MOLECULE: DNA POLYMERASE;
1442	6f2d-F	2.4	7	62	258	3	MOLECULE: FLAGELLAR BIOSYNTHETIC PROTEIN FLIP;
1443	6yaq-A	2.4	10.5	71	499	8	MOLECULE: CYTOKININ DEHYDROGENASE 8;
1444	7emf-J	2.4	8	61	122	13	MOLECULE: MEDIATOR OF RNA POLYMERASE II TRANSCRIPTION SUBUN
1445	6rax-N	2.4	11	69	207	9	MOLECULE: DNA REPLICATION LICENSING FACTOR MCM2;
1446	6f7s-C	2.4	16.8	96	319	8	MOLECULE: SERRATE RNA EFFECTOR MOLECULE HOMOLOG;
1447	1y6d-A	2.4	3.5	59	114	5	MOLECULE: PHOSPHORELAY PROTEIN LUXU;
1448	7rtm-A	2.4	20.1	71	571	7	MOLECULE: ELECTRONEUTRAL SODIUM BICARBONATE EXCHANGER 1;
1449	3fhf-A	2.4	12.2	55	214	5	MOLECULE: N-GLYCOSYLASE/DNA LYASE;
1450	6grd-A	2.4	14	78	407	14	MOLECULE: NUCLEASE-LIKE PROTEIN;
1451	2hsb-A	2.4	5.9	48	126	13	MOLECULE: HYPOTHETICAL UPF0332 PROTEIN AF0298;
1452	1ij6-A	2.4	3.4	54	305	6	MOLECULE: PLASMODIAL SPECIFIC LAV1-2 PROTEIN;
1453	4cht-B	2.4	7.3	58	215	7	MOLECULE: DNA TOPOISOMERASE 3-ALPHA;
1454	6wg3-A	2.4	29	49	561	2	MOLECULE: STRUCTURAL MAINTENANCE OF CHROMOSOMES PROTEIN 1A;
1455	1sfx-A	2.4	4.2	37	109	8	MOLECULE: CONSERVED HYPOTHETICAL PROTEIN AF2008;
1456	4zua-A	2.4	4.2	54	155	9	MOLECULE: EXOENZYME S SYNTHESIS REGULATORY PROTEIN EXSA;
1457	3bo0-A	2.4	13.9	77	442	4	MOLECULE: 23S RIBOSOMAL RNA;
1458	3p9y-A	2.4	10.3	49	198	6	MOLECULE: CG14216;
1459	4wid-A	2.4	8.5	66	353	11	MOLECULE: RHUL123;
1460	5lsw-A	2.4	8.2	75	273	7	MOLECULE: CELL DIFFERENTIATION PROTEIN RCD1 HOMOLOG;
1461	5ewp-A	2.4	13.1	84	241	6	MOLECULE: ARO (ARMADILLO REPEATS ONLY PROTEIN);
1462	5jp6-A	2.4	5.2	56	339	5	MOLECULE: PUTATIVE POLYSACCHARIDE DEACETYLASE;
1463	3mx2-B	2.4	22.4	79	517	8	MOLECULE: NUCLEOPROTEIN;
1464	6tdv-H	2.4	9.1	58	388	14	MOLECULE: ATPTB1;
1465	3rc8-A	2.4	8.2	88	609	8	MOLECULE: ATP-DEPENDENT RNA HELICASE SUPV3L1, MITOCHONDRIAL
1466	6rth-A	2.4	12	80	504	4	MOLECULE: RTX TOXIN AND CA2+-BINDING PROTEIN;
1467	2ve7-D	2.4	5	73	242	3	MOLECULE: KINETOCHORE PROTEIN HEC1, KINETOCHORE PROTEIN SPC

1468	2i7x-A	2.4	4.6	61	499	5	MOLECULE: PROTEIN CFT2;
1469	2xeq-C	2.4	12.2	68	241	21	MOLECULE: PAT1 HOMOLOG 1,;
1470	6l82-A	2.4	7.3	65	98	12	MOLECULE: SPINDLE POLE BODY COMPONENT;
1471	2jdi-H	2.4	6.5	45	88	7	MOLECULE: ATP SYNTHASE SUBUNIT ALPHA HEART ISOFORM;
1472	5ijz-K	2.4	7.3	65	304	8	MOLECULE: NADP-SPECIFIC GLUTAMATE DEHYDROGENASE;
1473	6ubz-A	2.3	8.4	76	792	5	MOLECULE: UNCHARACTERIZED PROTEIN GOXA;
1474	4ok7-A	2.3	7.3	88	223	6	MOLECULE: ENDOLYSIN;
1475	2pbi-A	2.3	14.7	86	415	12	MOLECULE: REGULATOR OF G-PROTEIN SIGNALING 9;
1476	2lck-A	2.3	15.7	99	296	5	MOLECULE: MITOCHONDRIAL UNCOUPLING PROTEIN 2;
1477	1qdm-A	2.3	4.1	56	430	4	MOLECULE: PROPHYTEPSIN;
1478	6bbm-D	2.3	8.7	65	455	6	MOLECULE: REPLICATIVE DNA HELICASE;
1479	3wvo-A	2.3	10	78	543	4	MOLECULE: CRISPR-ASSOCIATED PROTEIN, CSE1 FAMILY;
1480	4cth-A	2.3	11.8	83	698	11	MOLECULE: NEPRILYSIN;
1481	6wcw-A	2.3	11.3	90	247	2	MOLECULE: RAS-RELATED PROTEIN RAB-7A;
1482	2ncj-A	2.3	4.2	54	171	19	MOLECULE: UNCHARACTERIZED PROTEIN;
1483	6tpk-A	2.3	4.1	69	461	7	MOLECULE: OXYTOCIN RECEPTOR;
1484	1sf9-A	2.3	5.8	65	118	8	MOLECULE: YFHH HYPOTHETICAL PROTEIN;
1485	1knz-A	2.3	7.1	53	154	6	MOLECULE: 5'-R(*UP*GP*AP*CP*C)-3';
1486	6hgc-A	2.3	10.4	62	304	5	MOLECULE: UBIQUITIN CARBOXYL-TERMINAL HYDROLASE CALYPSO,UBI
1487	6jbn-A	2.3	7.4	66	391	5	MOLECULE: PEROXIDASE EFEB;
1488	6hk5-B	2.3	4.8	36	66	11	MOLECULE: COOJ;
1489	2q06-A	2.3	5.8	71	467	6	MOLECULE: NUCLEOPROTEIN;
1490	7c79-B	2.3	4	54	793	7	MOLECULE: RIBONUCLEASE MRP RNA SUBUNIT NME1;
1491	6ymw-A	2.3	10.7	87	913	14	MOLECULE: MITOCHONDRIAL TRANSCRIPTION FACTOR 1;
1492	2a3l-A	2.3	5.5	50	616	6	MOLECULE: AMP DEAMINASE;
1493	4aup-B	2.3	4.6	54	123	7	MOLECULE: PHOSPHOLIPASE A2 GROUP XIII;
1494	2n39-A	2.3	4.7	60	108	8	MOLECULE: CHROMODOMAIN-HELICASE-DNA-BINDING PROTEIN 1;
1495	6z6e-A	2.3	9.4	57	104	9	MOLECULE: TERMINASE SMALL SUBUNIT;
1496	2xwb-F	2.3	15.5	71	714	6	MOLECULE: COMPLEMENT C3B BETA CHAIN;
1497	6bfi-B	2.3	6.5	79	806	5	MOLECULE: VIN1;
1498	4cgz-A	2.3	10.9	61	629	8	MOLECULE: BLOOM'S SYNDROME HELICASE;
1499	1rfz-A	2.3	3.8	70	164	9	MOLECULE: HYPOTHETICAL PROTEIN APC35681;
1500	3ont-A	2.3	8.1	65	112	8	MOLECULE: SPOT 14 PROTEIN;
1501	3jd5-b	2.3	13.1	51	135	8	MOLECULE: 28S RIBOSOMAL RNA, MITOCHONDIAL;
1502	6jhm-A	2.3	11.5	97	494	5	MOLECULE: CHLOROPHENOL MONOOXYGENASE;

1503	3l9f-A	2.3	6.6	62	170	5	MOLECULE: PUTATIVE UNCHARACTERIZED PROTEIN SMU.1604C;
1504	5uke-A	2.3	7.6	78	124	10	MOLECULE: INTERLEUKIN-1 RECEPTOR-ASSOCIATED KINASE 3;
1505	5ip0-D	2.3	3.3	41	108	15	MOLECULE: PHA GRANULE-ASSOCIATED PROTEIN;
1506	7die-A	2.3	3.4	46	164	2	MOLECULE: FERRITIN;
1507	5kko-C	2.3	6.3	46	55	7	MOLECULE: UNCHARACTERISED PROTEIN;
1508	6vin-A	2.3	11.6	83	363	5	MOLECULE: THREONINE ASPARTASE 1;
1509	4x8d-A	2.3	4.3	66	429	5	MOLECULE: SULFOXIDE SYNTHASE EGTB;
1510	1tu3-J	2.3	3.2	35	53	11	MOLECULE: RAS-RELATED PROTEIN RAB-5A;
1511	6lea-C	2.3	4.8	54	126	13	MOLECULE: FLAGELLAR SECRETION CHAPERONE FLIS;
1512	5nmo-A	2.3	5.5	57	162	14	MOLECULE: CHROMOSOME PARTITION PROTEIN SMC,CHROMOSOME
1513	2p22-C	2.3	5.5	49	186	10	MOLECULE: SUPPRESSOR PROTEIN STP22 OF TEMPERATURE-SENSITIVE
1514	1w6j-A	2.3	14.8	95	727	5	MOLECULE: LANOSTEROL SYNTHASE;
1515	3c46-A	2.3	7.3	102	1095	10	MOLECULE: VIRION RNA POLYMERASE;
1516	6uxe-B	2.3	4.2	63	85	6	MOLECULE: CYSTEINE DESULFURASE, MITOCHONDRIAL;
1517	4dnr-A	2.3	10.6	103	1031	8	MOLECULE: CATION EFFLUX SYSTEM PROTEIN CUSB;
1518	6ef3-A	2.3	13.2	68	247	12	MOLECULE: PROTEASOME SUBUNIT BETA TYPE-1;
1519	3kyj-A	2.3	4.3	64	129	11	MOLECULE: PUTATIVE HISTIDINE PROTEIN KINASE;
1520	6wq2-A	2.3	6.8	60	154	10	MOLECULE: A-DNA;
1521	5cwb-A	2.3	3.8	70	197	13	MOLECULE: DESIGNED HELICAL REPEAT PROTEIN;
1522	6zr2-d	2.3	4.9	56	120	2	MOLECULE: NADH-UBIQUINONE OXIDOREDUCTASE CHAIN 3;
1523	5y69-A	2.3	8.8	67	147	10	MOLECULE: CHAIN A;
1524	2pyq-A	2.3	8	47	114	6	MOLECULE: UNCHARACTERIZED PROTEIN;
1525	6csv-A	2.3	5.1	52	90	8	MOLECULE: CENTROSOMAL PROTEIN OF 63 KDA,CENTROSOMAL PROTEIN
1526	6oap-A	2.3	10.5	76	305	8	MOLECULE: DUAL SENSOR HISTIDINE KINASE;
1527	6rkw-A	2.3	10.4	66	828	3	MOLECULE: DNA GYRASE SUBUNIT A;
1528	6ptg-B	2.3	6.9	49	86	4	MOLECULE: DNAK SUPPRESSOR;
1529	3q4i-A	2.3	12.9	69	204	9	MOLECULE: PHOSPHOHYDROLASE (MUTT/NUDIX FAMILY PROTEIN);
1530	7lmz-C	2.3	10.7	66	752	5	MOLECULE: TRANSITIONAL ENDOPLASMIC RETICULUM ATPASE;
1531	6jx7-A	2.3	9.1	80	1245	11	MOLECULE: FELINE INFECTIOUS PERITONITIS VIRUS SPIKE PROTEIN
1532	7bwk-A	2.3	7	57	113	4	MOLECULE: ICMO (DOTL);
1533	3ke3-A	2.3	9.8	58	371	12	MOLECULE: PUTATIVE SERINE-PYRUVATE AMINOTRANSFERASE;
1534	7b6d-D	2.3	3.1	47	219	2	MOLECULE: TRAFFICKING PROTEIN PARTICLE COMPLEX SUBUNIT;
1535	3j9a-A	2.3	7.3	66	81	9	MOLECULE: CAPSID PROTEIN VP26 HOMOLOG;
1536	3fey-A	2.3	5.2	76	761	8	MOLECULE: NUCLEAR CAP-BINDING PROTEIN SUBUNIT 1;
1537	7ljn-A	2.3	12.6	80	342	4	MOLECULE: CD-NTASE;

1538	3l0i-C	2.3	4.2	49	322	8	MOLECULE: DRRA;
1539	6rwx-A	2.3	7.6	59	189	14	MOLECULE: PROTEIN MXIG;
1540	6sjl-A	2.3	5.4	53	324	8	MOLECULE: PUTATIVE TYPE VI SECRETION PROTEIN;
1541	4xwj-A	2.3	3.8	66	153	9	MOLECULE: REGULATOR OF SIGMA D;
1542	6l80-C	2.3	7.5	49	107	6	MOLECULE: GAMMA-TUBULIN COMPLEX SUBUNIT MOD21;
1543	5j6f-A	2.3	6.5	77	352	9	MOLECULE: 3-DEOXY-D-ARABINO-HEPTULOSONATE 7-PHOSPHATE SYNTH
1544	6r6n-A	2.3	3.7	58	111	7	MOLECULE: SMALL SOLUBLE CYT C;
1545	3ck9-B	2.3	7.4	87	515	5	MOLECULE: SUSD;
1546	1zs3-A	2.3	10.3	66	171	8	MOLECULE: LACTOCOCCUS LACTIS MG1363 DPSA;
1547	1yoz-B	2.3	6	48	116	0	MOLECULE: HYPOTHETICAL PROTEIN AF0941;
1548	6ahp-A	2.3	2.3	46	110	9	MOLECULE: FLAGELLAR PROTEIN FLIL;
1549	2db7-A	2.3	4	36	57	6	MOLECULE: HAIRY/ENHANCER-OF-SPLIT RELATED WITH YRPW MOTIF
1550	4r04-A	2.3	20.2	92	1793	9	MOLECULE: TOXIN A;
1551	7ls0-B	2.3	4.5	69	362	10	MOLECULE: ALK TYROSINE KINASE RECEPTOR FUSED WITH ALK AND L
1552	6ero-A	2.3	3.7	64	296	9	MOLECULE: DIMETHYLADENOSINE TRANSFERASE 2, MITOCHONDRIAL,
1553	4xk8-k	2.3	2.6	36	46	6	MOLECULE: PHOTOSYSTEM I P700 CHLOROPHYLL A APOPROTEIN A1;
1554	3izq-1	2.3	13.1	55	516	5	MOLECULE: PROTEIN DOM34;
1555	7kfl-A	2.3	4.4	66	356	14	MOLECULE: MYOSIN-17;
1556	3t3o-A	2.3	5.9	57	553	11	MOLECULE: METAL DEPENDENT HYDROLASE;
1557	5xmg-A	2.3	7.7	64	344	6	MOLECULE: UNCHARACTERIZED PROTEIN;
1558	1g4u-S	2.3	10.3	70	360	13	MOLECULE: PROTEIN TYROSINE PHOSPHATASE SPTP;
1559	4roe-A	2.3	10.6	60	307	7	MOLECULE: TRANSCRIPTION FACTOR IIIB 50 KDA SUBUNIT;
1560	5azm-A	2.3	10.8	62	254	5	MOLECULE: N-ACETYLGLUCOSAMINIDASE;
1561	6v6d-A	2.3	4.2	60	209	10	MOLECULE: PANNEXIN-1;
1562	7ole-K	2.3	5	44	394	5	MOLECULE: RUVB-LIKE 1;
1563	1zpv-C	2.3	4.9	48	88	10	MOLECULE: ACT DOMAIN PROTEIN;
1564	3b77-A	2.3	8.9	54	188	6	MOLECULE: UNCHARACTERIZED PROTEIN;
1565	5k7l-A	2.3	13	51	701	6	MOLECULE: POTASSIUM VOLTAGE-GATED CHANNEL SUBFAMILY H MEMBE
1566	1zp2-A	2.3	4.6	70	227	9	MOLECULE: RNA POLYMERASE II HOLOENZYME CYCLIN-LIKE SUBUNIT;
1567	1gh6-A	2.3	5.2	54	114	9	MOLECULE: LARGE T ANTIGEN;
1568	2js1-A	2.3	5.3	51	80	8	MOLECULE: UNCHARACTERIZED PROTEIN YVFG;
1569	6m3q-F	2.3	11.1	86	301	8	MOLECULE: ANKYRIN-2;
1570	2qby-B	2.3	7.5	61	368	10	MOLECULE: CELL DIVISION CONTROL PROTEIN 6 HOMOLOG 1;
1571	5tsz-A	2.3	4.9	54	130	4	MOLECULE: PV CELL-TRAVERSAL PROTEIN;
1572	6vwb-A	2.3	3.5	50	90	8	MOLECULE: CROSSOVER JUNCTION ENDONUCLEASE MUS81;

1573	3nqw-A	2.3	5.1	46	178	9	MOLECULE: CG11900;
1574	6o9l-3	2.3	4.3	54	309	6	MOLECULE: DNA-DIRECTED RNA POLYMERASE II SUBUNIT RPB1;
1575	5w7p-A	2.3	14.5	73	397	7	MOLECULE: OXAC;
1576	4wai-A	2.3	4.3	49	91	14	MOLECULE: COMF OPERON PROTEIN 2;
1577	2pff-A	2.3	8.5	68	1683	7	MOLECULE: FATTY ACID SYNTHASE SUBUNIT ALPHA;
1578	5lsk-D	2.3	3.7	44	178	5	MOLECULE: PROTEIN MIS12 HOMOLOG;
1579	4eek-A	2.3	6	64	229	5	MOLECULE: BETA-PHOSPHOGLUCOMUTASE-RELATED PROTEIN;
1580	5l4k-P	2.3	15.7	78	456	5	MOLECULE: 26S PROTEASOME NON-ATPASE REGULATORY SUBUNIT 4;
1581	2rdc-B	2.3	5.3	64	135	8	MOLECULE: UNCHARACTERIZED PROTEIN;
1582	2e87-A	2.3	6.6	82	356	9	MOLECULE: HYPOTHETICAL PROTEIN PH1320;
1583	5i6c-A	2.3	7.1	98	480	10	MOLECULE: URIC ACID-XANTHINE PERMEASE;
1584	4ozq-B	2.3	11.1	81	687	6	MOLECULE: CHIMERA OF MALTOSE-BINDING PERIPLASMIC PROTEIN AN
1585	4y7s-A	2.3	3.2	60	111	7	MOLECULE: SURFACE ANTIGEN PROTEIN 2;
1586	5j9q-D	2.3	8.3	52	120	8	MOLECULE: HISTONE ACETYLTRANSFERASE ESA1;
1587	5xsj-L	2.3	5.9	58	122	9	MOLECULE: PERIPLASMIC BINDING PROTEIN/LACI TRANSCRIPTIONAL
1588	6lkc-B	2.3	14.1	86	533	6	MOLECULE: POLYUNSATURATED FATTY ACID SYNTHASE PFAD;
1589	5jcp-A	2.3	15.3	82	360	6	MOLECULE: ARF-GAP WITH RHO-GAP DOMAIN, ANK REPEAT AND PH DO
1590	5x3q-A	2.3	8	72	313	6	MOLECULE: ENVELOPE GLYCOPROTEIN;
1591	2ffl-A	2.3	13.5	87	732	6	MOLECULE: DICER;
1592	2f22-B	2.3	3.8	60	143	2	MOLECULE: BH3987;
1593	2qnd-B	2.3	2.7	39	143	13	MOLECULE: FMR1 PROTEIN;
1594	1sed-A	2.3	2.9	45	112	16	MOLECULE: HYPOTHETICAL PROTEIN YHAI;
1595	6tdw-H	2.3	9.1	58	249	14	MOLECULE: ATPTB1;
1596	5jbr-A	2.3	4.2	41	149	7	MOLECULE: UNCHARACTERIZED PROTEIN BCAV_2135;
1597	2m5z-A	2.3	2.9	40	44	13	MOLECULE: ENTEROCIN JSA;
1598	5tj5-A	2.3	6.8	56	570	4	MOLECULE: V-TYPE PROTON ATPASE SUBUNIT A;
1599	5lox-1	2.3	15.2	68	241	0	MOLECULE: PEPTIDASE;
1600	4ycz-B	2.3	7.7	81	590	7	MOLECULE: FUSION PROTEIN OF SEC13 AND NUP145C;
1601	5oeu-A	2.3	8.3	100	460	7	MOLECULE: GLUTATHIONE SYNTHETASE-LIKE EFFECTOR 22 (GPA-GSS2
1602	3ct9-A	2.3	5	61	346	8	MOLECULE: ACETYLORNITHINE DEACETYLASE;
1603	6xwx-B	2.3	5.9	53	84	6	MOLECULE: UNCHARACTERIZED PROTEIN,UNCHARACTERIZED PROTEIN;
1604	5vyk-C	2.3	8	59	203	12	MOLECULE: CHIMERA PROTEIN OF BRS DOMAIN OF BRAF AND CC-SAM
1605	4hlq-C	2.3	4.1	66	281	9	MOLECULE: TBC1 DOMAIN FAMILY MEMBER 20;
1606	6au8-A	2.3	14.6	65	280	5	MOLECULE: GOLGI TO ER TRAFFIC PROTEIN 4 HOMOLOG;
1607	4zvc-A	2.3	6.1	61	116	8	MOLECULE: DIGUANYLATE CYCLASE DOSC;

1608	6cs2-C	2.3	11.8	83	893	13	MOLECULE: SPIKE GLYCOPROTEIN,FIBRITIN;
1609	6hua-B	2.3	5.8	47	229	9	MOLECULE: UNCHARACTERIZED PROTEIN;
1610	2fbq-A	2.3	11.5	57	213	7	MOLECULE: PROBABLE TRANSCRIPTIONAL REGULATOR;
1611	7p5v-B	2.3	4.8	75	732	5	MOLECULE: VOLUME-REGULATED ANION CHANNEL SUBUNIT LRRC8A;
1612	5tfp-A	2.3	6.3	47	59	4	MOLECULE: HISTONE-LYSINE N-METHYLTRANSFERASE SETDB2;
1613	5kp7-B	2.3	2.8	43	79	21	MOLECULE: CURD;
1614	3ogi-B	2.3	5.3	55	89	5	MOLECULE: PUTATIVE ESAT-6-LIKE PROTEIN 6;
1615	2x0l-A	2.3	4.9	55	670	9	MOLECULE: LYSINE-SPECIFIC HISTONE DEMETHYLASE 1;
1616	6j1x-B	2.3	8	71	472	10	MOLECULE: NEDD4-LIKE E3 UBIQUITIN-PROTEIN LIGASE WWP1;
1617	6psi-A	2.3	10.2	70	280	7	MOLECULE: CHAPERONE PROTEIN DNAJ 2;
1618	6wuc-I	2.3	8.2	77	713	4	MOLECULE: INNER KINETOCHORE SUBUNIT MCM16;
1619	7ar9-J	2.3	4	47	145	9	MOLECULE: ND3;
1620	2pms-C	2.3	5	46	109	13	MOLECULE: LACTOTRANSFERRIN;
1621	2pc1-A	2.3	10	58	173	7	MOLECULE: ACETYLTRANSFERASE, GNAT FAMILY;
1622	4wfc-B	2.3	10.6	69	118	9	MOLECULE: EXOSOME COMPLEX EXONUCLEASE RRP6;
1623	3bzk-A	2.3	5.4	48	728	8	MOLECULE: TEX;
1624	5od9-A	2.3	8.8	54	95	7	MOLECULE: MID1SC9;
1625	5an3-B	2.3	6.4	53	136	9	MOLECULE: SGT1;
1626	1kdo-A	2.3	7.8	60	223	8	MOLECULE: CYTIDYLATE KINASE;
1627	5l09-B	2.3	4.2	43	164	12	MOLECULE: QUORUM-SENSING TRANSCRIPTIONAL ACTIVATOR;
1628	6w1s-Y	2.3	9.4	61	132	8	MOLECULE: MEDIATOR OF RNA POLYMERASE II TRANSCRIPTION SUBUN
1629	6cuq-B	2.3	3.3	47	117	6	MOLECULE: MACROPHAGE MIGRATION INHIBITORY FACTOR-LIKE PROTE
1630	4fym-D	2.3	12.2	65	227	12	MOLECULE: OROTATE PHOSPHORIBOSYLTRANSFERASE;
1631	5wah-A	2.3	6.4	67	99	9	MOLECULE: IGA FC RECEPTOR;
1632	6l7r-A	2.3	8.8	61	105	5	MOLECULE: PUTATIVE SPINDLE POLE BODY COMPONENT ALP6 PROTEIN
1633	4l8k-A	2.3	8.4	86	315	6	MOLECULE: PUTATIVE PEPTIDASE;
1634	2ex5-A	2.3	14.9	57	207	11	MOLECULE: I-CEUI DNA TARGET SITE;
1635	3fmt-A	2.3	3.3	47	162	15	MOLECULE: PROTEIN SEQA;
1636	3ay5-A	2.3	10.7	96	298	9	MOLECULE: CYCLIN-D1-BINDING PROTEIN 1;
1637	4oyd-B	2.3	3.9	46	117	9	MOLECULE: APOPTOSIS REGULATOR BHRF1;
1638	1jr3-E	2.3	14.7	69	334	10	MOLECULE: DNA POLYMERASE III SUBUNIT GAMMA;
1639	6btm-C	2.3	4.4	62	457	10	MOLECULE: ALTERNATIVE COMPLEX III SUBUNIT A;
1640	4hr1-B	2.3	4.7	61	119	10	MOLECULE: PUTATIVE UNCHARACTERIZED PROTEIN;
1641	6zfw-A	2.3	3.1	39	70	3	MOLECULE: PEROXIN-14;
1642	6tg9-D	2.3	2.9	46	69	0	MOLECULE: FORMATE DEHYDROGENASE SUBUNIT ALPHA;

1643	4ec5-A	2.3	13.2	69	218	10	MOLECULE: GENERAL SECRETION PATHWAY PROTEIN D;
1644	5nv9-A	2.3	4.6	55	480	2	MOLECULE: PUTATIVE SODIUM:SOLUTE SYMPORTER;
1645	1t7s-A	2.3	7.8	68	129	6	MOLECULE: BAG-1 COCHAPERONE;
1646	5a0j-A	2.3	9.3	76	311	8	MOLECULE: LABDANE-RELATED DITERPENE SYNTHASE;
1647	5b86-A	2.3	8.4	85	579	9	MOLECULE: TUMOR NECROSIS FACTOR ALPHA-INDUCED PROTEIN 2;
1648	6u45-A	2.3	15	99	726	9	MOLECULE: ELONGATION FACTOR 2;
1649	6mdm-D	2.3	11.1	80	713	4	MOLECULE: VESICLE-FUSING ATPASE;
1650	6yxq-A	2.3	5.6	81	188	4	MOLECULE: ACTIVATING SIGNAL COINTEGRATOR 1 COMPLEX SUBUNIT
1651	4hte-A	2.3	12.2	71	340	10	MOLECULE: NICKING ENZYME;
1652	1ii0-B	2.3	3.5	53	553	4	MOLECULE: ARSENICAL PUMP-DRIVING ATPASE;
1653	1fxk-C	2.3	6.5	60	133	7	MOLECULE: PREFOLDIN;
1654	3g9g-A	2.3	10.7	66	250	12	MOLECULE: SUPPRESSOR OF YEAST PROFILIN DELETION;
1655	3trc-A	2.3	4.1	53	168	9	MOLECULE: PHOSPHOENOLPYRUVATE-PROTEIN PHOSPHOTRANSFERASE;
1656	3fp5-A	2.3	3.3	63	106	5	MOLECULE: ACYL-COA BINDING PROTEIN;
1657	3plt-A	2.3	2.9	51	214	14	MOLECULE: SPHINGOLIPID LONG CHAIN BASE-RESPONSIVE PROTEIN L
1658	7c7e-A	2.3	5	61	142	5	MOLECULE: PUTATIVE DNA-BINDING TRANSCRIPTIONAL REGULATOR;
1659	5aww-G	2.3	5.6	46	75	9	MOLECULE: PROTEIN TRANSLOCASE SUBUNIT SECY;
1660	6r7i-D	2.3	13.1	84	407	7	MOLECULE: COP9 SIGNALOSOME COMPLEX SUBUNIT 1;
1661	3i0o-A	2.3	8.9	82	329	4	MOLECULE: SPECTINOMYCIN PHOSPHOTRANSFERASE;
1662	6w17-H	2.3	6.6	54	132	7	MOLECULE: ACTIN-RELATED PROTEIN 3;
1663	7nsc-H	2.3	9.7	83	158	10	MOLECULE: RAN-BINDING PROTEIN 9;
1664	5h69-A	2.3	5	48	252	4	MOLECULE: CHROMOSOME PARTITION PROTEIN SMC;
1665	5n3u-B	2.3	10.7	59	170	12	MOLECULE: PHYCOCYANOBILIN LYASE SUBUNIT ALPHA;
1666	3bqz-A	2.3	12.1	71	186	8	MOLECULE: HTH-TYPE TRANSCRIPTIONAL REGULATOR QACR;
1667	6o3q-A	2.3	4.9	39	49	8	MOLECULE: VICILIN;
1668	7d3u-F	2.3	4.5	46	85	9	MOLECULE: MONOVALENT NA+/H+ ANTIPORTER SUBUNIT D;
1669	3jvo-G	2.3	3.9	49	97	6	MOLECULE: GP6;
1670	1osd-A	2.3	3.8	44	72	7	MOLECULE: HYPOTHETICAL PROTEIN MERP;
1671	6es9-A	2.3	11.2	84	545	8	MOLECULE: ACYL-COA DEHYDROGENASE;
1672	6qg0-G	2.3	11.7	62	355	5	MOLECULE: TRANSLATION INITIATION FACTOR EIF-2B SUBUNIT ALPH
1673	4bjm-A	2.3	8.4	66	226	6	MOLECULE: AVRM;
1674	5n9y-A	2.3	3.3	41	327	10	MOLECULE: ZINC TRANSPORT PROTEIN ZNTB;
1675	5xq3-A	2.3	17.1	78	901	5	MOLECULE: PCRGLX PROTEIN;
1676	6f2d-G	2.3	3.3	44	89	5	MOLECULE: FLAGELLAR BIOSYNTHETIC PROTEIN FLIP;
1677	1e7p-A	2.3	10.3	67	655	1	MOLECULE: FUMARATE REDUCTASE FLAVOPROTEIN SUBUNIT;

1678	2kru-A	2.3	3.8	47	63	13	MOLECULE: LIGHT-INDEPENDENT PROTOCHLOROPHYLLIDE REDUCTASE S
1679	6nd4-Q	2.3	8	63	862	2	MOLECULE: ETS RRNA;
1680	5z51-A	2.3	6.1	46	161	9	MOLECULE: DNA PRIMASE;
1681	5k94-A	2.3	11.3	57	506	14	MOLECULE: MALTOSE-BINDING PERIPLASMIC PROTEIN,PROTEIN TRANS
1682	7pow-B	2.3	4.9	60	205	5	MOLECULE: CDP-DIACYLGLYCEROL--SERINE O-PHOSPHATIDYLTRANSFER
1683	7ar9-M	2.3	10.3	93	438	10	MOLECULE: ND3;
1684	3gwl-A	2.3	5.3	59	106	7	MOLECULE: FAD-LINKED SULFHYDRYL OXIDASE;
1685	5zki-B	2.3	8.8	49	304	8	MOLECULE: NUCLEASE EXOG, MITOCHONDRIAL;
1686	1a8r-A	2.3	11.9	76	221	7	MOLECULE: GTP CYCLOHYDROLASE I;
1687	6xmg-A	2.3	9.7	77	638	6	MOLECULE: CRISPR-CAS;
1688	5lc5-n	2.3	10.3	63	166	8	MOLECULE: NADH-UBIQUINONE OXIDOREDUCTASE CHAIN 3;
1689	7ns4-b	2.3	8	59	340	3	MOLECULE: E3 UBIQUITIN-PROTEIN LIGASE RMD5;
1690	7a46-A	2.3	9.7	66	223	11	MOLECULE: PUTATIVE TRANSPORT PROTEIN;
1691	6swy-5	2.3	13.3	91	789	9	MOLECULE: VACUOLAR IMPORT AND DEGRADATION PROTEIN 28;
1692	5izo-A	2.3	7.2	61	290	10	MOLECULE: BIFUNCTIONAL OLIGORIBONUCLEASE AND PAP PHOSPHATAS
1693	7f3h-D	2.3	3.4	61	464	8	MOLECULE: BIFUNCTIONAL CYTOCHROME P450/NADPH--P450 REDUCTAS
1694	5mmc-A	2.3	2.2	35	70	3	MOLECULE: PEROXIN 14;
1695	6z7p-A	2.3	8.5	75	1025	4	MOLECULE: S-LAYER PROTEIN;
1696	4ily-B	2.3	12.4	76	250	7	MOLECULE: CHITOSANASE;
1697	6s6y-L	2.3	4.1	61	311	3	MOLECULE: FORMYLMETHANOFURAN DEHYDROGENASE SUBUNIT A;
1698	6a5n-A	2.3	14	84	502	4	MOLECULE: HISTONE-LYSINE N-METHYLTRANSFERASE, H3 LYSINE-9 S
1699	6jo5-K	2.3	3.3	41	45	2	MOLECULE: PHOTOSYSTEM I P700 CHLOROPHYLL A APOPROTEIN A1;
1700	6oce-A	2.3	8.3	82	689	7	MOLECULE: STRESS-GATED CATION CHANNEL 1.2;
1701	5i0n-A	2.3	16.8	100	489	6	MOLECULE: PHOSPHATIDYLINOSITOL 4-KINASE TYPE 2-ALPHA,LYSOZY
1702	4o5p-A	2.2	10.7	74	742	1	MOLECULE: UNCHARACTERIZED PROTEIN;
1703	1u96-A	2.2	3	45	69	9	MOLECULE: CYTOCHROME C OXIDASE COPPER CHAPERONE;
1704	2qnl-A	2.2	6.1	59	162	5	MOLECULE: UNCHARACTERIZED PROTEIN;
1705	1z0x-A	2.2	12.8	67	216	10	MOLECULE: TRANSCRIPTIONAL REGULATOR, TETR FAMILY;
1706	6pvg-A	2.2	7.2	75	445	5	MOLECULE: FAD MONOOXYGENASE;
1707	1w36-C	2.2	22.3	142	1121	8	MOLECULE: EXODEOXYRIBONUCLEASE V BETA CHAIN;
1708	2gsc-B	2.2	7.7	58	117	2	MOLECULE: PUTATIVE UNCHARACTERIZED PROTEIN XCC0516;
1709	7cgp-J	2.2	5	57	83	5	MOLECULE: MITOCHONDRIAL IMPORT INNER MEMBRANE TRANSLOCASE S
1710	6tdv-A	2.2	22.4	92	486	5	MOLECULE: ATPTB1;
1711	3bpx-A	2.2	4.3	49	147	4	MOLECULE: TRANSCRIPTIONAL REGULATOR;
1712	5xyf-A	2.2	5.5	45	192	7	MOLECULE: TERF1-INTERACTING NUCLEAR FACTOR 2;

1713	7cae-E	2.2	10.9	64	443	6	MOLECULE: ABC TRANSPORTER, ATP-BINDING PROTEIN SUGC;
1714	1zs4-A	2.2	7.2	46	82	4	MOLECULE: DNA - 27MER;
1715	6s3l-K	2.2	6.5	49	193	6	MOLECULE: FLAGELLAR BIOSYNTHETIC PROTEIN FLIP;
1716	6rmo-I	2.2	5	75	432	3	MOLECULE: IMP-SPECIFIC 5'-NUCLEOTIDASE, PUTATIVE;
1717	2oap-1	2.2	12	53	498	9	MOLECULE: TYPE II SECRETION SYSTEM PROTEIN;
1718	1vp7-B	2.2	7	52	77	17	MOLECULE: EXODEOXYRIBONUCLEASE VII SMALL SUBUNIT;
1719	5u30-A	2.2	8.7	72	1085	10	MOLECULE: CRISPR-ASSOCIATED ENDONUCLEASE C2C1;
1720	6ekr-A	2.2	5	76	303	4	MOLECULE: TYPE II SITE-SPECIFIC DEOXYRIBONUCLEASE;
1721	3b5m-B	2.2	13.3	64	195	9	MOLECULE: UNCHARACTERIZED PROTEIN;
1722	4bjt-A	2.2	4.8	63	152	10	MOLECULE: DNA-BINDING PROTEIN RAP1;
1723	7kuw-A	2.2	7.3	56	62	7	MOLECULE: SEQUENCE-BASED DESIGNED PROTEIN NMT_0994_GUIDED_0
1724	7b54-X	2.2	4.2	87	1732	5	MOLECULE: VAR2CSA IN PRESENCE OF PLCS, DBL1-DBL4,ERYTHROCYT
1725	2guz-B	2.2	5.5	43	65	9	MOLECULE: MITOCHONDRIAL IMPORT INNER MEMBRANE TRANSLOCASE S
1726	2n5j-A	2.2	4.4	43	49	5	MOLECULE: RIBONUCLEASE ZC3H12A;
1727	3u8p-A	2.2	8.6	70	336	9	MOLECULE: CYTOCHROME B562 INTEGRAL FUSION WITH ENHANCED GRE
1728	2p5t-E	2.2	5.8	62	95	10	MOLECULE: FRAGMENT OF PEZA HELIX-TURN-HELIX MOTIF;
1729	6toa-D	2.2	3.6	44	195	11	MOLECULE: ADAPTOR PROTEIN RCC01688;
1730	5lj3-G	2.2	7	50	97	10	MOLECULE: U5 SNRNA (SMALL NUCLEAR RNA);
1731	7bqr-A	2.2	2.8	48	137	21	MOLECULE: MUSSOC;
1732	4gfq-A	2.2	3.1	46	186	9	MOLECULE: RIBOSOME-RECYCLING FACTOR;
1733	4a9a-A	2.2	13.8	62	356	10	MOLECULE: RIBOSOME-INTERACTING GTPASE 1;
1734	5e5w-B	2.2	8	51	149	18	MOLECULE: HEMAGGLUTININ-ESTERASE;
1735	2kw2-A	2.2	4.6	54	101	7	MOLECULE: SPECIALIZED ACYL CARRIER PROTEIN;
1736	3gac-C	2.2	3.3	47	117	13	MOLECULE: MACROPHAGE MIGRATION INHIBITORY FACTOR-LIKE
1737	4y13-A	2.2	11.6	68	244	4	MOLECULE: TRANSCRIPTIONAL REGULATOR OF FTSQAZ GENE CLUSTER;
1738	6fnp-A	2.2	13.5	65	158	3	MOLECULE: MEMBRANE PROTEIN;
1739	6wg3-E	2.2	14	57	1226	7	MOLECULE: STRUCTURAL MAINTENANCE OF CHROMOSOMES PROTEIN 1A;
1740	6bv7-A	2.2	3.9	41	57	12	MOLECULE: SODIUM/CALCIUM EXCHANGER 1;
1741	7d3u-D	2.2	7.7	83	534	10	MOLECULE: MONOVALENT NA+/H+ ANTIPORTER SUBUNIT D;
1742	6qly-A	2.2	13.7	87	330	8	MOLECULE: E3 UBIQUITIN-PROTEIN LIGASE MYLIP;
1743	6rjb-B	2.2	10.3	57	622	2	MOLECULE: TRANSKETOLASE;
1744	4ckg-A	2.2	12.1	85	368	11	MOLECULE: ARF-GAP WITH COILED-COIL, ANK REPEAT AND PH DOMAI
1745	6fon-A	2.2	2.7	40	252	15	MOLECULE: COPPER CHAPERONE FOR SUPEROXIDE DISMUTASE;
1746	7tbs-A	2.2	14.6	64	221	16	MOLECULE: GLUTAREDOXIN 2;
1747	2jxu-A	2.2	4.1	56	153	7	MOLECULE: TERB;

1748	4uzz-A	2.2	4	52	108	2	MOLECULE: INTRAFLAGELLAR TRANSPORT COMPLEX B PROTEIN 46
1749	4fyg-A	2.2	8.9	72	743	7	MOLECULE: SIDF, INHIBITOR OF GROWTH FAMILY, MEMBER 3;
1750	5a5t-L	2.2	10.9	90	372	2	MOLECULE: EUKARYOTIC TRANSLATION INITIATION FACTOR 3 SUBUNI
1751	7apk-i	2.2	10.8	59	275	8	MOLECULE: THO COMPLEX SUBUNIT 1;
1752	3u9j-A	2.2	3.3	68	157	7	MOLECULE: F-BOX/LRR-REPEAT PROTEIN 5;
1753	6dku-A	2.2	2.9	43	125	7	MOLECULE: VP35;
1754	7cm3-A	2.2	8.7	100	1278	3	MOLECULE: SODIUM LEAK CHANNEL NON-SELECTIVE PROTEIN;
1755	6ar7-C	2.2	4.5	63	207	8	MOLECULE: UNCHARACTERIZED PROTEIN;
1756	3mse-B	2.2	5.5	62	168	2	MOLECULE: CALCIUM-DEPENDENT PROTEIN KINASE, PUTATIVE;
1757	6zr2-e	2.2	13.2	68	105	9	MOLECULE: NADH-UBIQUINONE OXIDOREDUCTASE CHAIN 3;
1758	6msr-A	2.2	5.6	54	76	17	MOLECULE: PRO-2.5;
1759	1fe8-C	2.2	11.2	59	191	2	MOLECULE: VON WILLEBRAND FACTOR;
1760	6o84-A	2.2	7.6	95	415	11	MOLECULE: LOC100127796 PROTEIN,LOC100127796 PROTEIN,OTOP3,
1761	5im3-A	2.2	10.1	69	874	7	MOLECULE: RIBONUCLEOSIDE-DIPHOSPHATE REDUCTASE;
1762	2kw7-A	2.2	3	52	157	15	MOLECULE: CONSERVED DOMAIN PROTEIN;
1763	3ub0-D	2.2	6.8	63	194	16	MOLECULE: NON-STRUCTURAL PROTEIN 6, NSP6,;
1764	6fvb-A	2.2	6.7	86	1001	6	MOLECULE: IMPORTIN BETA-LIKE PROTEIN KAP120;
1765	6f2p-A	2.2	15	80	1040	4	MOLECULE: PAENIBACILLUS XANTHAN LYASE;
1766	3mk7-C	2.2	8.1	56	303	9	MOLECULE: CYTOCHROME C OXIDASE, CBB3-TYPE, SUBUNIT N;
1767	4iw7-A	2.2	10.1	68	335	9	MOLECULE: 8-AMINO-7-OXONONANOATE SYNTHASE;
1768	4lmg-B	2.2	3.9	50	121	10	MOLECULE: IRON-REGULATED TRANSCRIPTIONAL ACTIVATOR AFT2;
1769	3l09-C	2.2	4.5	81	253	5	MOLECULE: PUTATIVE TRANSCRIPTIONAL REGULATOR;
1770	2vz9-B	2.2	4.3	82	2086	5	MOLECULE: FATTY ACID SYNTHASE;
1771	6nbn-A	2.2	4	65	123	5	MOLECULE: AAEL005772-PA;
1772	6kth-A	2.2	8	57	190	7	MOLECULE: JUVENILE HORMONE DIOL KINASE;
1773	7nnl-B	2.2	9.1	74	682	5	MOLECULE: POTASSIUM-TRANSPORTING ATPASE POTASSIUM-BINDING S
1774	5d06-A	2.2	8	114	1526	15	MOLECULE: UNCHARACTERIZED PROTEIN;
1775	3k1r-A	2.2	5.6	59	192	5	MOLECULE: HARMONIN;
1776	2a7o-A	2.2	3.8	51	100	8	MOLECULE: HUNTINGTIN INTERACTING PROTEIN B;
1777	6xz4-A	2.2	16.4	78	304	8	MOLECULE: TALIN ROD DOMAIN-CONTAINING PROTEIN 1;
1778	7a23-X	2.2	10.9	47	103	4	MOLECULE: 51KDA;
1779	4v1f-A	2.2	4.7	45	86	2	MOLECULE: F0F1 ATP SYNTHASE SUBUNIT C;
1780	5lm2-B	2.2	7.7	71	340	14	MOLECULE: TYROSINE-PROTEIN PHOSPHATASE NON-RECEPTOR TYPE 23
1781	2o3f-A	2.2	3.3	50	83	14	MOLECULE: PUTATIVE HTH-TYPE TRANSCRIPTIONAL REGULATOR YBBH;
1782	6c4v-A	2.2	2.6	45	81	9	MOLECULE: POLYKETIDE SYNTHASE PKS13;

1783	3pas-B	2.2	10.8	59	192	8	MOLECULE: TETR FAMILY TRANSCRIPTION REGULATOR;
1784	7lga-D	2.2	3.7	49	91	10	MOLECULE: RETROTRANSPOSON-DERIVED PROTEIN PEG10;
1785	4nqi-D	2.2	6.3	71	232	11	MOLECULE: SH3 DOMAIN-CONTAINING PROTEIN;
1786	3rzs-A	2.2	3.5	59	119	7	MOLECULE: OBP14;
1787	2gd5-D	2.2	12	61	162	8	MOLECULE: CHARGED MULTIVESICULAR BODY PROTEIN 3;
1788	6u4k-A	2.2	7.2	58	391	12	MOLECULE: TALIN-2;
1789	6v9z-A	2.2	10.9	54	715	4	MOLECULE: ABC-TYPE BACTERIOCIN TRANSPORTER;
1790	3utk-A	2.2	3.1	44	95	7	MOLECULE: LIPOPROTEIN OUTS;
1791	2hf6-A	2.2	9.6	54	149	11	MOLECULE: COATOMER SUBUNIT ZETA-1;
1792	3mn2-A	2.2	5.1	63	108	11	MOLECULE: PROBABLE ARAC FAMILY TRANSCRIPTIONAL REGULATOR;
1793	6iie-A	2.2	5.5	50	87	12	MOLECULE: DIACYLGLYCEROL KINASE ALPHA;
1794	4c5f-A	2.2	3.3	61	330	5	MOLECULE: MEMBRANE-BOUND LYTIC MUREIN TRANSGLYCOSYLASE C;
1795	6v4l-A	2.2	11.9	70	479	7	MOLECULE: TRK SYSTEM POTASSIUM UPTAKE PROTEIN TRKH;
1796	2dzn-F	2.2	3	44	69	9	MOLECULE: PROBABLE 26S PROTEASOME REGULATORY SUBUNIT P28;
1797	5u70-A	2.2	9.5	81	901	10	MOLECULE: POTASSIUM CHANNEL SUBFAMILY T MEMBER 1;
1798	2bw3-A	2.2	14	63	518	16	MOLECULE: TRANSPOSASE;
1799	3aei-A	2.2	4.5	40	94	10	MOLECULE: PREFOLDIN BETA SUBUNIT 2;
1800	1hyn-Q	2.2	12	59	302	2	MOLECULE: BAND 3 ANION TRANSPORT PROTEIN;
1801	3zc7-B	2.2	7	40	63	5	MOLECULE: ADENOSINE MONOPHOSPHATE-PROTEIN TRANSFERASE VBHT;
1802	3h4z-B	2.2	10	71	563	8	MOLECULE: MALTOSE-BINDING PERIPLASMIC PROTEIN FUSED WITH AL
1803	2mbg-A	2.2	4.4	60	265	5	MOLECULE: RALA-BINDING PROTEIN 1;
1804	6ww7-C	2.2	8.9	67	209	6	MOLECULE: ER MEMBRANE PROTEIN COMPLEX SUBUNIT 1;
1805	1og6-C	2.2	12.8	56	298	9	MOLECULE: HYPOTHETICAL OXIDOREDUCTASE YDHF;
1806	1zae-A	2.2	8.1	52	70	13	MOLECULE: EARLY PROTEIN GP16.7;
1807	2p0n-A	2.2	2.7	52	161	6	MOLECULE: HYPOTHETICAL PROTEIN NMB1532;
1808	6wb9-6	2.2	4	47	98	11	MOLECULE: ENDOPLASMIC RETICULUM MEMBRANE PROTEIN COMPLEX SU
1809	3vw4-A	2.2	2.2	44	122	9	MOLECULE: REP;
1810	5k8c-A	2.2	5.4	76	358	4	MOLECULE: 3-DEOXY-ALPHA-D-MANNO-OCTULOSONATE 8-OXIDASE;
1811	5a34-C	2.2	5.9	74	170	7	MOLECULE: BIFUNCTIONAL GLUTAMATE/PROLINE--TRNA LIGASE;
1812	5h0j-A	2.2	10.4	61	234	7	MOLECULE: UNCHARACTERIZED PROTEIN;
1813	7n8n-B	2.2	3.3	55	202	7	MOLECULE: HISTONE H4-H3 DOUBLET;
1814	3tdw-A	2.2	8.8	76	302	4	MOLECULE: GENTAMICIN RESISTANCE PROTEIN;
1815	2ymb-D	2.2	13.6	65	231	12	MOLECULE: MIT DOMAIN-CONTAINING PROTEIN 1;
1816	7my4-A	2.2	9.4	48	66	15	MOLECULE: SPERM AUTOANTIGENIC PROTEIN 17;
1817	3ed5-A	2.2	21	65	232	11	MOLECULE: YFNB;

1818	1wud-A	2.2	3	41	77	12	MOLECULE: ATP-DEPENDENT DNA HELICASE RECQ;
1819	6iu4-A	2.2	9.7	68	225	10	MOLECULE: VIT1;
1820	3o3u-N	2.2	11.2	76	580	4	MOLECULE: MALTOSE-BINDING PERIPLASMIC PROTEIN, ADVANCED GLY
1821	2omo-A	2.2	11.3	52	105	6	MOLECULE: DUF176;
1822	4xa6-A	2.2	7.1	55	168	2	MOLECULE: GP7-MYH7(1777-1855)-EB1 CHIMERA PROTEIN;
1823	5kkp-A	2.2	16.9	67	509	1	MOLECULE: PSEUDOURIDYLATE SYNTHASE 7;
1824	1v32-A	2.2	8.4	55	101	9	MOLECULE: HYPOTHETICAL PROTEIN RAFL09-47-K03;
1825	2ntz-A	2.2	11.6	87	183	11	MOLECULE: 5'-
1826	6mrs-A	2.2	2.6	38	77	18	MOLECULE: PEAK6;
1827	3c7n-A	2.2	7.9	76	648	3	MOLECULE: HEAT SHOCK PROTEIN HOMOLOG SSE1;
1828	6jmg-A	2.2	9.2	65	267	14	MOLECULE: DNAJ HOMOLOG SUBFAMILY C MEMBER 27-A;
1829	2ptf-A	2.2	11.7	52	200	12	MOLECULE: UNCHARACTERIZED PROTEIN MTH_863;
1830	6wqz-A	2.2	4.5	57	536	9	MOLECULE: AUTOPHAGY-RELATED PROTEIN 9A;
1831	3ziu-A	2.2	8.4	74	621	12	MOLECULE: LEUCYL-TRNA SYNTHETASE;
1832	3twb-C	2.2	4.4	67	417	4	MOLECULE: PUTATIVE DEHYDRATASE;
1833	5h3i-D	2.2	4.9	53	94	9	MOLECULE: PUTATIVE ACYL-COA-BINDING PROTEIN;
1834	3fvq-B	2.2	13.2	80	349	3	MOLECULE: FE(3+) IONS IMPORT ATP-BINDING PROTEIN FBPC;
1835	4eyy-Q	2.2	13.3	63	188	8	MOLECULE: ICMR;
1836	5mc1-B	2.2	11.6	87	487	7	MOLECULE: XAA-PRO DIPEPTIDASE;
1837	3iwf-B	2.2	4.8	56	90	4	MOLECULE: TRANSCRIPTION REGULATOR RPIR FAMILY;
1838	6psd-A	2.2	2.3	37	73	0	MOLECULE: EF-HAND CALCIUM-BINDING DOMAIN-CONTAINING PROTEIN
1839	3uw3-B	2.2	7.6	68	375	9	MOLECULE: ASPARTATE-SEMIALDEHYDE DEHYDROGENASE;
1840	2dae-A	2.2	3	44	75	14	MOLECULE: KIAA0733 PROTEIN;
1841	2cru-A	2.2	10.6	60	118	10	MOLECULE: PROGRAMMED CELL DEATH PROTEIN 5;
1842	6ek4-A	2.2	12.7	69	342	7	MOLECULE: PAXB;
1843	7ad3-A	2.2	5.2	98	298	7	MOLECULE: PHEROMONE ALPHA FACTOR RECEPTOR;
1844	2vsg-A	2.2	5.7	67	358	4	MOLECULE: VARIANT SURFACE GLYCOPROTEIN ILTAT 1.24;
1845	2fbi-A	2.2	6	53	136	11	MOLECULE: PROBABLE TRANSCRIPTIONAL REGULATOR;
1846	5uyo-A	2.2	4.7	34	44	9	MOLECULE: HEEH_RD4_0097;
1847	2y9z-B	2.2	14.4	82	595	6	MOLECULE: IMITATION SWITCH PROTEIN 1 (DEL_ATPASE);
1848	2nb2-A	2.2	2.5	37	38	11	MOLECULE: NIGELLIN-1.1;
1849	3ce8-A	2.2	4.7	46	90	7	MOLECULE: PUTATIVE PII-LIKE NITROGEN REGULATORY PROTEIN;
1850	6zka-K	2.2	5.6	50	98	4	MOLECULE: NADH-UBIQUINONE OXIDOREDUCTASE CHAIN 3;
1851	7f9m-A	2.2	14.9	71	158	4	MOLECULE: RIFIN;
1852	5bs1-D	2.2	6.5	69	121	6	MOLECULE: CRRBCX-IIA;

1853	3c4r-C	2.2	4.3	84	127	10	MOLECULE: UNCHARACTERIZED PROTEIN;
1854	6rd4-2	2.2	13.3	90	441	4	MOLECULE: ASA-10: POLYTOMELLA F-ATP SYNTHASE ASSOCIATED SUB
1855	4pl0-B	2.2	11.9	77	576	3	MOLECULE: MICROCIN-J25 EXPORT ATP-BINDING/PERMEASE PROTEIN
1856	1i7q-A	2.2	4.2	64	517	13	MOLECULE: ANTHRANILATE SYNTHASE;
1857	5fd4-A	2.2	13.7	89	309	8	MOLECULE: COMR;
1858	2gta-A	2.2	5.7	56	98	11	MOLECULE: HYPOTHETICAL PROTEIN YPJD;
1859	3qth-B	2.2	4	75	164	8	MOLECULE: UNCHARACTERIZED PROTEIN;
1860	2p62-A	2.2	10.1	69	241	7	MOLECULE: HYPOTHETICAL PROTEIN PH0156;
1861	2iho-A	2.2	5	56	292	4	MOLECULE: LECTIN;
1862	2w96-A	2.2	10.9	76	249	7	MOLECULE: CELL DIVISION PROTEIN KINASE 4;
1863	6lcu-A	2.2	14	79	756	15	MOLECULE: MTSASE;
1864	5adz-A	2.2	10.6	67	566	4	MOLECULE: ALKYLDIHYDROXYACETONEPHOSPHATE SYNTHASE,
1865	6ygh-D	2.2	3	46	193	7	MOLECULE: CAPSID PROTEIN;
1866	6vmb-g	2.2	5.2	49	323	4	MOLECULE: ATP SYNTHASE SUBUNIT ALPHA, CHLOROPLASTIC;
1867	1rj1-A	2.2	5.9	66	148	2	MOLECULE: INVERTASE INHIBITOR;
1868	3hc1-A	2.2	13.1	78	298	9	MOLECULE: UNCHARACTERIZED HDOD DOMAIN PROTEIN;
1869	2owy-A	2.2	7.5	60	306	12	MOLECULE: RECOMBINATION-ASSOCIATED PROTEIN RDGC;
1870	7ef9-A	2.2	12.5	64	414	9	MOLECULE: ADENINE DNA GLYCOSYLASE;
1871	7m4m-A	2.2	4.4	47	173	13	MOLECULE: E3 UBIQUITIN-PROTEIN LIGASE RNF216;
1872	6dlm-A	2.2	4	38	74	8	MOLECULE: DHD127_A;
1873	5zhy-B	2.2	4.6	40	89	8	MOLECULE: SPIKE GLYCOPROTEIN, SPIKE GLYCOPROTEIN;
1874	7etw-A	2.2	5.2	92	197	4	MOLECULE: INSULIN-INDUCED GENE 2 PROTEIN;
1875	6tky-A	2.2	10.5	65	417	9	MOLECULE: DEDICATOR OF CYTOKINESIS PROTEIN 10;
1876	6w1s-J	2.2	2.9	53	167	8	MOLECULE: MEDIATOR OF RNA POLYMERASE II TRANSCRIPTION SUBUN
1877	3vej-B	2.2	3	37	41	8	MOLECULE: UBIQUITIN-LIKE PROTEIN MDY2;
1878	5eik-A	2.2	3.8	68	234	10	MOLECULE: UNCHARACTERIZED PROTEIN Y57A10A.28;
1879	7auf-A	2.2	2.7	47	89	15	MOLECULE: SIMILAR TO ACYL CARRIER PROTEIN;
1880	2x3m-A	2.2	4.7	58	166	5	MOLECULE: HYPOTHETICAL PROTEIN ORF239;
1881	2i15-A	2.2	5.5	40	122	5	MOLECULE: HYPOTHETICAL PROTEIN MG296 HOMOLOG;
1882	4art-A	2.2	14.8	75	237	4	MOLECULE: STRUCTURAL PROTEIN ORF273;
1883	7lma-A	2.2	4.9	74	1012	3	MOLECULE: TELOMERASE LA-RELATED PROTEIN P65;
1884	4dvy-P	2.2	7.3	57	656	9	MOLECULE: CYTOTOXICITY-ASSOCIATED IMMUNODOMINANT ANTIGEN;
1885	6hyp-A	2.2	12.1	80	2272	6	MOLECULE: MIDASIN,MIDASIN;
1886	2bf0-X	2.2	3.8	52	131	4	MOLECULE: PCF11;
1887	6fsf-A	2.2	3.9	68	218	15	MOLECULE: GTPASE-ACTIVATING PROTEIN BEM3;

1888	6p4o-F	2.2	18.3	65	481	11	MOLECULE: DNA-DEPENDENT ATPASE XPBII;
1889	2fuq-A	2.2	14.6	83	747	6	MOLECULE: HEPARINASE II PROTEIN;
1890	6khj-E	2.2	4.5	54	101	13	MOLECULE: NAD(P)H-QUINONE OXIDOREDUCTASE SUBUNIT 1;
1891	5cuy-D	2.2	13.4	81	396	9	MOLECULE: ACIDOCALCISOMAL PYROPHOSPHATASE;
1892	6f0k-H	2.2	4.5	75	156	8	MOLECULE: CYTOCHROME C FAMILY PROTEIN;
1893	6qeq-A	2.2	3.3	39	111	5	MOLECULE: PCFF;
1894	3dsq-B	2.2	3.1	47	282	6	MOLECULE: PYRROLYSYL-TRNA SYNTHETASE;
1895	6e4j-A	2.2	3.9	54	72	13	MOLECULE: UNCHARACTERIZED PROTEIN PF2048.1;
1896	5ted-B	2.2	3.3	41	210	7	MOLECULE: LMO0488 PROTEIN;
1897	2vrz-A	2.2	7.7	52	98	10	MOLECULE: VIRULENCE FACTOR ESXA;
1898	6zh3-A	2.2	8.6	57	170	5	MOLECULE: VACUOLAR PROTEIN-SORTING-ASSOCIATED PROTEIN 24;
1899	6ec8-A	2.2	7	65	803	5	MOLECULE: LANTIBIOTIC DEHYDRATASE DOMAIN PROTEIN;
1900	4lur-A	2.2	8.2	69	309	7	MOLECULE: INTERPHOTORECEPTOR RETINOID-BINDING PROTEIN(IRBP)
1901	7eev-B	2.2	17.6	61	399	7	MOLECULE: GTP CYCLOHYDROLASE II;
1902	5uay-A	2.2	7.1	73	302	4	MOLECULE: PROTEIN TOC75-3, CHLOROPLASTIC;
1903	6rwy-g	2.2	4.3	42	86	7	MOLECULE: INNER ROD PROTEIN;
1904	4oh3-B	2.2	17.7	98	537	4	MOLECULE: NITRATE TRANSPORTER 1.1;
1905	3bhp-C	2.2	5.6	43	54	5	MOLECULE: UPF0291 PROTEIN YNZC;
1906	5lsk-N	2.2	8.7	62	173	6	MOLECULE: PROTEIN MIS12 HOMOLOG;
1907	2oev-A	2.2	5.8	64	697	6	MOLECULE: PROGRAMMED CELL DEATH 6-INTERACTING PROTEIN;
1908	1u2m-C	2.2	9.4	60	143	2	MOLECULE: HISTONE-LIKE PROTEIN HLP-1;
1909	5fjd-B	2.2	4.2	49	111	6	MOLECULE: COPPER STORAGE PROTEIN 1;
1910	5gpa-A	2.2	7	42	189	2	MOLECULE: TRANSCRIPTIONAL REGULATOR (TETR/ACRR FAMILY);
1911	7m7a-A	2.2	8.1	74	538	11	MOLECULE: PHOSPHOINOSITIDE 3-KINASE MAVQ;
1912	3lk7-A	2.2	14.3	79	448	11	MOLECULE: UDP-N-ACETYLMURAMOYLALANINE--D-GLUTAMATE LIGASE;
1913	4fhn-B	2.2	8.9	62	1022	8	MOLECULE: NUCLEOPORIN NUP37;
1914	5uaw-B	2.2	15.4	59	281	3	MOLECULE: PYRROLINE-5-CARBOXYLATE REDUCTASE 1, MITOCHONDRIA
1915	3u8v-A	2.2	4.4	52	83	13	MOLECULE: METAL-BINDING PROTEIN SMBP;
1916	1z9h-A	2.2	10.1	72	274	4	MOLECULE: MEMBRANE-ASSOCIATED PROSTAGLANDIN E SYNTHASE-2;
1917	5mmj-n	2.2	8.9	53	99	6	MOLECULE: 50S RIBOSOMAL PROTEIN L31;
1918	5wu1-A	2.2	4.2	67	493	7	MOLECULE: SPECKLE TARGETED PIP5K1A-REGULATED POLY(A) POLYME
1919	4ps2-A	2.2	4.2	46	79	9	MOLECULE: PUTATIVE TYPE VI SECRETION PROTEIN;
1920	1z0p-A	2.2	5.5	47	73	6	MOLECULE: HYPOTHETICAL PROTEIN SPY1572;
1921	3zym-C	2.2	11	65	280	3	MOLECULE: PHOSPHATIDYLINOSITOL-BINDING CLATHRIN ASSEMBLY PR
1922	5vtl-A	2.2	7.5	65	210	14	MOLECULE: TB427.07.360- PUTATIVE UNCHARACTERIZED METACYCLIC

1923	6h02-A	2.2	20.6	85	1334	5	MOLECULE: MEDIATOR OF RNA POLYMERASE II TRANSCRIPTION SUBUN
1924	5ca9-A	2.2	12.8	79	656	8	MOLECULE: PROTEIN SEY1;
1925	5xsi-A	2.2	5.1	49	333	4	MOLECULE: PHOSPHODIESTERASE ACTING ON CYCLIC DINUCLEOTIDES;
1926	5n5e-A	2.2	4.1	55	98	9	MOLECULE: PFC_05175;
1927	4ryk-A	2.2	9.7	68	295	9	MOLECULE: LMO0325 PROTEIN;
1928	5jqz-A	2.2	4.8	42	75	7	MOLECULE: DE NOVO DESIGNED HOMOTETRAMER;
1929	2kob-A	2.2	8	43	108	9	MOLECULE: UNCHARACTERIZED PROTEIN;
1930	6ioh-A	2.2	5.5	77	375	6	MOLECULE: HOMOSERINE O-ACETYLTRANSFERASE;
1931	5cet-A	2.2	11.2	52	328	10	MOLECULE: BIFUNCTIONAL OLIGORIBONUCLEASE AND PAP PHOSPHATAS
1932	4dnh-A	2.2	3.2	42	386	7	MOLECULE: UNCHARACTERIZED PROTEIN;
1933	1w0b-A	2.2	6.5	57	102	9	MOLECULE: ALPHA-HEMOGLOBIN STABILIZING PROTEIN;
1934	5ydn-A	2.2	3.9	45	109	13	MOLECULE: GENE PRODUCT J;
1935	2cs1-A	2.2	5.4	43	92	7	MOLECULE: PMS1 PROTEIN HOMOLOG 1;
1936	5mlq-A	2.2	6.5	86	226	6	MOLECULE: CDPS;
1937	6g0z-A	2.2	7.1	71	290	10	MOLECULE: RIBOSOME BIOGENESIS GTPASE A;
1938	3f0c-A	2.2	11.9	71	193	8	MOLECULE: TRANSCRIPTIONAL REGULATOR;
1939	3k2j-A	2.2	3.2	60	121	5	MOLECULE: PROTEIN POLYBROMO-1;
1940	6s8g-G	2.2	3.8	50	244	2	MOLECULE: LIPOPOLYSACCHARIDE ABC TRANSPORTER, ATP-BINDING P
1941	5lnk-Y	2.2	15.2	64	171	9	MOLECULE: MITOCHONDRIAL COMPLEX I, 51 KDA SUBUNIT;
1942	2dsy-D	2.2	4.4	46	81	9	MOLECULE: HYPOTHETICAL PROTEIN TTHA0281;
1943	6drh-E	2.2	4.5	81	366	11	MOLECULE: ADP-RIBOSYL-(DINITROGEN REDUCTASE) HYDROLASE;
1944	2eyq-A	2.2	10.3	72	1146	8	MOLECULE: TRANSCRIPTION-REPAIR COUPLING FACTOR;
1945	7pt0-A	2.2	12	69	201	12	MOLECULE: TETR FAMILY TRANSCRIPTIONAL REGULATOR;
1946	4x5m-B	2.2	2.6	46	93	11	MOLECULE: UNCHARACTERIZED PROTEIN;
1947	5eyb-A	2.2	4.2	59	340	7	MOLECULE: DNA-BINDING PROTEIN REB1;
1948	2n2h-B	2.2	5.8	47	125	6	MOLECULE: SIN3 HISTONE DEACETYLASE COREPRESSOR COMPLEX COMP
1949	2b8i-A	2.2	3.2	50	77	4	MOLECULE: PAS FACTOR;
1950	6iv9-A	2.2	6.4	77	874	4	MOLECULE: CAS13D;
1951	7mdy-B	2.2	6.3	78	411	3	MOLECULE: LIPOPROTEIN TRANSPORTER SUBUNIT LOLE;
1952	7bin-g	2.2	5.2	52	260	2	MOLECULE: FLAGELLAR BIOSYNTHETIC PROTEIN FLIP;
1953	6nr8-2	2.2	6.3	52	103	4	MOLECULE: PREFOLDIN SUBUNIT 1;
1954	7jsr-A	2.2	10.2	56	1496	4	MOLECULE: NAD-SPECIFIC GLUTAMATE DEHYDROGENASE;
1955	7pw5-B	2.2	11.2	84	702	6	MOLECULE: SMG1,SERINE/THREONINE-PROTEIN KINASE SMG1,SMG1,
1956	4a12-B	2.2	4.1	63	186	8	MOLECULE: TRANSCRIPTION FACTOR FAPR;
1957	1k7y-A	2.2	15.2	64	577	6	MOLECULE: METHIONINE SYNTHASE;

1958	5y77-A	2.2	12.9	55	456	9	MOLECULE: KYNURENINE 3-MONOOXYGENASE;
1959	5e1l-A	2.2	8	56	173	9	MOLECULE: CELL DIVISION PROTEIN ZAPC;
1960	2v57-B	2.2	4.3	51	176	6	MOLECULE: TETR FAMILY TRANSCRIPTIONAL REPRESSOR LFRR;
1961	6vr7-A	2.2	5.7	69	485	9	MOLECULE: MUR LIGASE MIDDLE DOMAIN PROTEIN;
1962	3mzy-A	2.2	3.5	47	123	15	MOLECULE: RNA POLYMERASE SIGMA-H FACTOR;
1963	6qct-A	2.2	4.7	70	722	6	MOLECULE: POLYMERASE ACIDIC PROTEIN;
1964	2yqy-A	2.2	3.4	53	126	6	MOLECULE: HYPOTHETICAL PROTEIN TTHA0303;
1965	3okg-B	2.2	4.3	50	391	14	MOLECULE: RESTRICTION ENDONUCLEASE S SUBUNITS;
1966	1vyi-A	2.2	3.2	66	111	6	MOLECULE: RNA POLYMERASE ALPHA SUBUNIT;
1967	3vou-A	2.2	4	58	139	3	MOLECULE: ION TRANSPORT 2 DOMAIN PROTEIN, VOLTAGE-GATED SOD
1968	3eo8-A	2.2	10.3	51	219	6	MOLECULE: BLUB-LIKE FLAVOPROTEIN;
1969	6aun-A	2.2	8.1	88	618	5	MOLECULE: PLA2G6, IPLA2BETA;
1970	3lpz-A	2.2	11.4	59	314	7	MOLECULE: UNCHARACTERIZED PROTEIN;
1971	6nzk-A	2.2	6.8	86	1175	8	MOLECULE: SPIKE SURFACE GLYCOPROTEIN;
1972	5c0w-K	2.2	15.8	93	576	8	MOLECULE: EXOSOME COMPLEX COMPONENT RRP45;
1973	5a1v-K	2.2	9.6	67	1125	6	MOLECULE: ADP-RIBOSYLATION FACTOR 1;
1974	5o9z-L	2.2	9.6	57	459	12	MOLECULE: PRE-MRNA-PROCESSING-SPLICING FACTOR 8;
1975	4xh3-A	2.1	11.1	59	214	2	MOLECULE: ACTIN-BINDING PROTEIN ANILLIN;
1976	3rh2-A	2.1	10.6	74	210	9	MOLECULE: HYPOTHETICAL TETR-LIKE TRANSCRIPTIONAL REGULATOR;
1977	3e8p-A	2.1	4.3	57	153	4	MOLECULE: UNCHARACTERIZED PROTEIN;
1978	1f51-B	2.1	7.3	63	182	10	MOLECULE: SPORULATION INITIATION PHOSPHOTRANSFERASE B;
1979	2xex-B	2.1	16.2	93	674	13	MOLECULE: ELONGATION FACTOR G;
1980	4lws-B	2.1	5.4	51	88	8	MOLECULE: UNCHARACTERIZED PROTEIN;
1981	6u8y-c	2.1	6.2	52	112	4	MOLECULE: MONOVALENT CATION/H+ ANTIPORTER SUBUNIT E;
1982	3a8r-B	2.1	10.9	80	167	10	MOLECULE: PUTATIVE UNCHARACTERIZED PROTEIN;
1983	4ayb-K	2.1	4.1	58	84	7	MOLECULE: DNA-DIRECTED RNA POLYMERASE;
1984	5of3-C	2.1	9.5	46	107	7	MOLECULE: DNA PRIMASE SMALL SUBUNIT PRIS;
1985	7dqk-B	2.1	3.7	56	479	7	MOLECULE: PROTEIN DETOXIFICATION;
1986	4gu7-A	2.1	5.2	69	310	12	MOLECULE: PUTATIVE UNCHARACTERIZED PROTEIN SCO7193;
1987	6c2s-A	2.1	5.6	83	142	14	MOLECULE: TRANSCRIPTIONAL REGULATOR, MARR FAMILY;
1988	3k1l-A	2.1	13	62	376	8	MOLECULE: FANCL;
1989	4r6i-A	2.1	14.7	85	472	9	MOLECULE: ANTHRAX TOXIN EXPRESSION TRANS-ACTING POSITIVE RE
1990	4n78-A	2.1	6.5	71	1184	14	MOLECULE: CYTOPLASMIC FMR1-INTERACTING PROTEIN 1;
1991	2pnk-A	2.1	9.7	48	424	4	MOLECULE: BH0493 PROTEIN;
1992	2p6v-A	2.1	9.4	55	97	7	MOLECULE: TRANSCRIPTION INITIATION FACTOR TFIID SUBUNIT 4;

1993	5mlp-A	2.1	5.7	64	255	9	MOLECULE: UNCHARACTERIZED PROTEIN;
1994	7crc-B	2.1	13.3	73	1112	10	MOLECULE: NAD+ HYDROLASE (NADASE);
1995	3dao-B	2.1	18.5	63	274	11	MOLECULE: PUTATIVE PHOSPHATE;
1996	2p01-A	2.1	9.9	74	323	7	MOLECULE: ALPHA-2-MACROGLOBULIN RECEPTOR-ASSOCIATED
1997	5woe-A	2.1	3.9	64	125	8	MOLECULE: SORTING NEXIN-25;
1998	6wpm-A	2.1	10.1	57	401	0	MOLECULE: SUBSTRATE-BINDING PROTEIN;
1999	6xp5-G	2.1	4.9	43	122	5	MOLECULE: MEDIATOR OF RNA POLYMERASE II TRANSCRIPTION SUBUN
2000	2yb5-F	2.1	7.1	65	215	9	MOLECULE: PUTATIVE FUSIDIC ACID RESISTANCE PROTEIN;
2001	2rq5-A	2.1	4.8	41	121	7	MOLECULE: PROTEIN JUMONJI;
2002	6whj-B	2.1	8.3	58	335	9	MOLECULE: RIBOKINASE;
2003	1gp8-A	2.1	5.8	36	40	11	MOLECULE: PROTEIN (SCAFFOLDING PROTEIN);
2004	7jr7-A	2.1	5.5	87	588	7	MOLECULE: ATP-BINDING CASSETTE SUB-FAMILY G MEMBER 5;
2005	7oq4-Z	2.1	3.5	48	118	23	MOLECULE: DNA-DIRECTED RNA POLYMERASE SUBUNIT A';
2006	2jnk-A	2.1	6.7	69	140	12	MOLECULE: HYALURONONGLUCOSAMINIDASE;
2007	5kzb-A	2.1	7.3	49	104	16	MOLECULE: VIRUS MATRIX PROTEIN;
2008	1l6n-A	2.1	5.6	76	288	5	MOLECULE: GAG POLYPROTEIN;
2009	7e4h-B	2.1	7.5	100	317	10	MOLECULE: SORTING ASSEMBLY MACHINERY 50 KDA SUBUNIT;
2010	4j19-A	2.1	2.9	46	77	9	MOLECULE: HOMEOBOX-CONTAINING PROTEIN 1;
2011	4yut-A	2.1	12.3	76	351	14	MOLECULE: FAMILY 3 ADENYLATE CYCLASE;
2012	6ph4-B	2.1	4.6	50	460	10	MOLECULE: BLUE-LIGHT-ACTIVATED HISTIDINE KINASE;
2013	6h0g-B	2.1	8.4	70	401	9	MOLECULE: DNA DAMAGE-BINDING PROTEIN 1,DNA DAMAGE-BINDING P
2014	7ob9-M	2.1	6.9	63	388	13	MOLECULE: DNA-DIRECTED RNA POLYMERASE I SUBUNIT RPA1;
2015	6yx5-B	2.1	5.9	91	339	4	MOLECULE: RAS-RELATED PROTEIN RAB-8A;
2016	7bin-L	2.1	5.1	47	75	11	MOLECULE: FLAGELLAR BIOSYNTHETIC PROTEIN FLIP;
2017	6u4o-A	2.1	10.7	116	479	5	MOLECULE: FUMARATE HYDRATASE;
2018	6rfq-9	2.1	7.1	57	86	5	MOLECULE: SUBUNIT NUAM OF NADH:UBIQUINONE OXIDOREDUCTASE (C
2019	5mlt-A	2.1	15.8	71	454	13	MOLECULE: ABC TRANSPORTER, SUBSTRATE-BINDING PROTEIN;
2020	6u8t-B	2.1	11.7	66	221	9	MOLECULE: PFHB2;
2021	5zxd-B	2.1	10.9	76	491	5	MOLECULE: ATP-BINDING CASSETTE SUB-FAMILY F MEMBER 1;
2022	2pjw-V	2.1	8	53	91	9	MOLECULE: UNCHARACTERIZED PROTEIN YHL002W;
2023	3l76-A	2.1	20.9	91	589	4	MOLECULE: ASPARTOKINASE;
2024	5ayk-A	2.1	9	74	744	7	MOLECULE: DNAJ HOMOLOG SUBFAMILY C MEMBER 10;
2025	5a31-N	2.1	10.7	94	703	5	MOLECULE: ANAPHASE-PROMOTING COMPLEX SUBUNIT 1;
2026	5fg0-A	2.1	4	50	407	6	MOLECULE: E3 UBIQUITIN-PROTEIN LIGASE LISTERIN;
2027	6l30-A	2.1	3.7	46	518	20	MOLECULE: PROTEIN ECT2;

2028	6rbf-B	2.1	14.8	79	364	8	MOLECULE: MUCIN-2;
2029	5uft-A	2.1	6.6	56	144	7	MOLECULE: NITROGEN-FIXING NIFU-LIKE, N-TERMINAL;
2030	4jd9-G	2.1	3.8	66	120	5	MOLECULE: 14.5 KDA SALIVARY PROTEIN;
2031	1v74-B	2.1	3.5	44	87	14	MOLECULE: COLICIN D;
2032	7bc4-B	2.1	8.3	87	2054	9	MOLECULE: FATTY ACID SYNTHASE SUBUNIT ALPHA;
2033	6hd8-A	2.1	9.5	47	461	4	MOLECULE: NANOBODY,MALTOSE/MALTODEXTRIN-BINDING PERIPLASMIC
2034	7ml7-A	2.1	16.2	95	1216	7	MOLECULE: TOXIN B;
2035	6egc-A	2.1	5.6	61	146	10	MOLECULE: SC_2L4HC2_23;
2036	4yxw-G	2.1	5.8	52	213	10	MOLECULE: ATP SYNTHASE SUBUNIT ALPHA, MITOCHONDRIAL;
2037	6vw7-D	2.1	9.6	63	514	6	MOLECULE: NAD-DEPENDENT FORMATE DEHYDROGENASE GAMMA
2038	4gr6-B	2.1	13.6	65	105	3	MOLECULE: ATRBCX2;
2039	5wlq-A	2.1	7.1	53	104	4	MOLECULE: CAPSID ASSEMBLY SCAFFOLDING PROTEIN,MYOSIN-7,MICR
2040	6nbu-A	2.1	4	59	126	15	MOLECULE: CRISPR-ASSOCIATED PROTEIN;
2041	2uxw-A	2.1	12.8	111	567	4	MOLECULE: VERY-LONG-CHAIN SPECIFIC ACYL-COA DEHYDROGENASE;
2042	6fea-C	2.1	4.5	54	113	15	MOLECULE: NITROGENASE PROTEIN ALPHA CHAIN;
2043	2h8r-A	2.1	5.8	81	176	7	MOLECULE: HEPATOCYTE NUCLEAR FACTOR 1-BETA;
2044	1vi7-A	2.1	11.1	68	206	3	MOLECULE: HYPOTHETICAL PROTEIN YIGZ;
2045	3gn4-A	2.1	8.1	61	129	10	MOLECULE: MYOSIN-VI;
2046	1fse-B	2.1	2.5	44	70	5	MOLECULE: GERE;
2047	3j25-A	2.1	15.4	100	638	7	MOLECULE: TETRACYCLINE RESISTANCE PROTEIN TETM;
2048	2jes-A	2.1	10.3	95	370	9	MOLECULE: PORTAL PROTEIN;
2049	3f46-A	2.1	13.1	57	345	5	MOLECULE: 5,10-METHENYLTETRAHYDROMETHANOPTERIN
2050	7cq3-A	2.1	4.9	80	298	3	MOLECULE: STRUCTURE-SPECIFIC ENDONUCLEASE SUBUNIT SLX1;
2051	4bxx-X	2.1	8.4	58	120	12	MOLECULE: DNA-DIRECTED RNA POLYMERASE II SUBUNIT RPB1;
2052	3npe-A	2.1	4.1	60	522	12	MOLECULE: 9-CIS-EPOXYCAROTENOID DIOXYGENASE 1, CHLOROPLASTI
2053	1nkl-A	2.1	2.9	46	78	13	MOLECULE: NK-LYSIN;
2054	3td7-A	2.1	4.8	82	253	9	MOLECULE: FAD-LINKED SULFHYDRYL OXIDASE R596;
2055	2jek-A	2.1	4.2	65	140	2	MOLECULE: RV1873;
2056	4zmk-A	2.1	4.7	47	71	6	MOLECULE: TELOMERE LENGTH REGULATOR TAZ1;
2057	6lk8-A	2.1	10.7	85	1409	4	MOLECULE: MGC83295 PROTEIN;
2058	3lf9-A	2.1	3.9	48	120	8	MOLECULE: 4E10_D0_1IS1A_001_C (T161);
2059	2nrk-A	2.1	3	46	165	2	MOLECULE: HYPOTHETICAL PROTEIN GRPB;
2060	5z68-C	2.1	4.4	73	373	7	MOLECULE: DNA REPLICATION AND REPAIR PROTEIN RECF;
2061	2r11-D	2.1	7.8	47	288	4	MOLECULE: CARBOXYLESTERASE NP;
2062	7c9m-C	2.1	11.7	65	260	5	MOLECULE: D-HISTIDINE 2-AMINOBUTANOYLTRANSFERASE;

2063	2f66-B	2.1	4	44	111	7	MOLECULE: SUPPRESSOR PROTEIN STP22 OF TEMPERATURE-
2064	1hrk-A	2.1	12.2	53	359	8	MOLECULE: FERROCHELATASE;
2065	6pns-A	2.1	13.1	87	1291	8	MOLECULE: RNA-DIRECTED RNA POLYMERASE;
2066	4av2-A	2.1	5.2	52	274	6	MOLECULE: TYPE IV PILUS BIOGENESIS AND COMPETENCE PROTEIN P
2067	7amy-A	2.1	4.2	59	353	8	MOLECULE: FLAGELLAR BIOSYNTHESIS PROTEIN FLHA;
2068	6o8b-B	2.1	9.7	81	657	4	MOLECULE: STIMULATOR OF INTERFERON GENES PROTEIN;
2069	4ilo-A	2.1	6	52	236	10	MOLECULE: CT398;
2070	6lum-D	2.1	9.3	63	146	8	MOLECULE: SUCCINATE DEHYDROGENASE SUBUNIT C;
2071	3lyq-B	2.1	9.3	58	186	12	MOLECULE: IPGB2;
2072	6oew-B	2.1	12.5	68	239	4	MOLECULE: CYTIDYLYLTRANSFERASE;
2073	6cn0-B	2.1	3.9	74	272	7	MOLECULE: 16S RRNA (GUANINE(1405)-N(7))-METHYLTRANSFERASE;
2074	1u9d-A	2.1	9.8	59	122	15	MOLECULE: HYPOTHETICAL PROTEIN VC0714;
2075	3tix-C	2.1	4.9	59	147	10	MOLECULE: UBIQUITIN-LIKE PROTEIN SMT3,RNA-INDUCED TRANSCRIP
2076	4asv-A	2.1	5.1	54	79	6	MOLECULE: SMALL GLUTAMINE-RICH TETRATRICOPEPTIDE REPEAT-CON
2077	3rkl-A	2.1	6.5	52	80	10	MOLECULE: STIV-A81;
2078	2i0z-A	2.1	12.5	82	416	9	MOLECULE: NAD(FAD)-UTILIZING DEHYDROGENASES;
2079	1pd3-A	2.1	3.8	36	54	6	MOLECULE: NONSTRUCTURAL PROTEIN NS2;
2080	6w6v-E	2.1	5.1	61	169	10	MOLECULE: RNA COMPONENT OF RNASE MRP NME1;
2081	2ksf-A	2.1	4.2	50	107	10	MOLECULE: SENSOR PROTEIN KDPD;
2082	6pe4-Q	2.1	4.1	66	369	12	MOLECULE: V-TYPE PROTON ATPASE SUBUNIT A, VACUOLAR ISOFORM;
2083	2o4c-A	2.1	3.6	53	380	4	MOLECULE: ERYTHRONATE-4-PHOSPHATE DEHYDROGENASE;
2084	3rfy-A	2.1	4	61	356	7	MOLECULE: PEPTIDYL-PROLYL CIS-TRANS ISOMERASE CYP38, CHLORO
2085	5muu-D	2.1	8.3	55	148	4	MOLECULE: MAJOR INNER PROTEIN P1;
2086	5mz2-D	2.1	12.3	69	482	6	MOLECULE: RUBISCO LARGE SUBUNIT;
2087	6hmj-A	2.1	4.4	53	359	6	MOLECULE: PUTATIVE PAS/PAC SENSOR PROTEIN;
2088	2i8e-A	2.1	2.6	39	88	0	MOLECULE: HYPOTHETICAL PROTEIN;
2089	6f7j-A	2.1	12.9	47	97	11	MOLECULE: SERRATE RNA EFFECTOR MOLECULE HOMOLOG;
2090	3af5-A	2.1	5.6	58	638	2	MOLECULE: PUTATIVE UNCHARACTERIZED PROTEIN PH1404;
2091	7eep-M	2.1	3.8	46	182	7	MOLECULE: PAM1 PORTAL PROTEINS;
2092	6h86-A	2.1	8	52	76	2	MOLECULE: SYNAPTONEMAL COMPLEX CENTRAL ELEMENT PROTEIN 3;
2093	7mpy-B	2.1	7.1	71	430	10	MOLECULE: FOLATE SYNTHESIS BIFUNCTIONAL PROTEIN;
2094	6yvd-D	2.1	4.8	74	414	9	MOLECULE: CONDENSIN COMPLEX SUBUNIT 2;
2095	6wge-B	2.1	9.1	76	490	12	MOLECULE: STRUCTURAL MAINTENANCE OF CHROMOSOMES PROTEIN 1A;
2096	2aj0-A	2.1	3.2	41	71	7	MOLECULE: PROBABLE CADMIUM-TRANSPORTING ATPASE;
2097	4v1a-m	2.1	7.4	71	109	7	MOLECULE: MITORIBOSOMAL PROTEIN ML37, MRPL37;

2098	7fas-A	2.1	8.1	68	1229	6	MOLECULE: ERYTHROCYTE MEMBRANE PROTEIN 1, PFEMP1;
2099	6ici-A	2.1	18.1	84	585	2	MOLECULE: [F-ACTIN]-MONOOXYGENASE MICAL3;
2100	6lo8-E	2.1	4.4	50	80	12	MOLECULE: MITOCHONDRIAL IMPORT INNER MEMBRANE TRANSLOCASE S
2101	5iqc-A	2.1	8	80	301	9	MOLECULE: BIFUNCTIONAL AAC/APH;
2102	1l8q-A	2.1	8.9	55	321	15	MOLECULE: CHROMOSOMAL REPLICATION INITIATOR PROTEIN DNAA;
2103	2my1-A	2.1	15.2	54	159	11	MOLECULE: PRE-MRNA-SPLICING FACTOR BUD31;
2104	5d1w-D	2.1	3	48	196	6	MOLECULE: RV3249C TRANSCRIPTIONAL REGULATOR;
2105	7aqo-J	2.1	7.1	55	230	4	MOLECULE: THO COMPLEX SUBUNIT 2;
2106	1p68-A	2.1	5.4	45	102	4	MOLECULE: DE NOVO DESIGNED PROTEIN S-824;
2107	1u5k-A	2.1	12.1	69	242	9	MOLECULE: HYPOTHETICAL PROTEIN;
2108	6l7o-C	2.1	11.2	53	121	9	MOLECULE: NAD(P)H-QUINONE OXIDOREDUCTASE SUBUNIT 1;
2109	6t5a-A	2.1	4.7	43	96	9	MOLECULE: TEGUMENT PROTEIN UL51;
2110	5k04-A	2.1	4.9	59	732	8	MOLECULE: UNCHARACTERIZED PROTEIN;
2111	2k19-A	2.1	3	50	98	6	MOLECULE: PUTATIVE PISCICOLIN 126 IMMUNITY PROTEIN;
2112	2ouw-B	2.1	14	52	136	6	MOLECULE: ALKYLHYDROPEROXIDASE AHPD CORE;
2113	2euf-A	2.1	4.1	64	244	9	MOLECULE: VIRAL CYCLIN;
2114	3at7-A	2.1	3.9	79	251	9	MOLECULE: ALGINATE-BINDING FLAGELLIN;
2115	5zib-A	2.1	5.5	87	579	6	MOLECULE: ALPHA-1,6-MANNOSYLGLYCOPROTEIN 6-BETA-N-
2116	7ogp-A	2.1	8.6	71	361	7	MOLECULE: PHIKZ055,PHIKZ056.1;
2117	1le6-B	2.1	4.2	57	123	9	MOLECULE: GROUP X SECRETORY PHOSPHOLIPASE A2;
2118	4hkr-A	2.1	6	56	165	9	MOLECULE: CALCIUM RELEASE-ACTIVATED CALCIUM CHANNEL PROTEIN
2119	6nhh-A	2.1	12.2	77	431	6	MOLECULE: CYTOCHROME B;
2120	6f70-A	2.1	8.5	84	242	11	MOLECULE: GLUTATHIONE TRANSFERASE;
2121	6k93-A	2.1	7.3	69	239	7	MOLECULE: TYPE III EFFECTOR XOPAI;
2122	2hsz-A	2.1	11.1	60	225	10	MOLECULE: NOVEL PREDICTED PHOSPHATASE;
2123	6hu9-c	2.1	4.8	80	269	8	MOLECULE: CYTOCHROME B-C1 COMPLEX SUBUNIT 1, MITOCHONDRIAL;
2124	5u0p-2	2.1	7.7	40	78	8	MOLECULE: MEDIATOR COMPLEX SUBUNIT 14;
2125	4oph-A	2.1	6.2	49	202	10	MOLECULE: NONSTRUCTURAL PROTEIN 1;
2126	2c0s-A	2.1	4.5	39	64	5	MOLECULE: CONSERVED DOMAIN PROTEIN;
2127	5n77-A	2.1	8	83	257	5	MOLECULE: MAGNESIUM TRANSPORT PROTEIN CORA;
2128	3c18-A	2.1	11.5	78	290	8	MOLECULE: NUCLEOTIDYLTRANSFERASE-LIKE PROTEIN;
2129	6eys-B	2.1	10.2	63	478	10	MOLECULE: PVDP;
2130	4om3-D	2.1	5.6	49	122	4	MOLECULE: TRANSDUCIN-LIKE ENHANCER PROTEIN 1;
2131	6fxd-A	2.1	9.4	55	128	9	MOLECULE: MUPZ;
2132	1eqf-A	2.1	12.4	83	267	5	MOLECULE: RNA POLYMERASE II TRANSCRIPTION INITIATION

2133	6lcc-A	2.1	8.8	58	375	5	MOLECULE: AATPS;
2134	6ndu-A	2.1	5	58	170	5	MOLECULE: PARKIN COREGULATED GENE PROTEIN;
2135	2nyf-A	2.1	8.1	80	511	11	MOLECULE: NOSTOC PUNCTIFORME PHENYLALANINE AMMONIA LYASE;
2136	5flv-M	2.1	4.7	58	229	10	MOLECULE: HOMEOBOX PROTEIN NKX-2.5, T-BOX TRANSCRIPTION FAC
2137	4p17-B	2.1	9.2	76	290	9	MOLECULE: RABGAP/TBC PROTEIN;
2138	3j7a-E	2.1	9	69	185	1	MOLECULE: 18S RIBOSOMAL RNA;
2139	4ow5-A	2.1	9.3	61	307	8	MOLECULE: FUSOLIN;
2140	4q28-A	2.1	3.1	43	110	7	MOLECULE: PERIPLAKIN;
2141	1of9-A	2.1	2.7	47	77	11	MOLECULE: PORE-FORMING PEPTIDE AMEOBAPORE A;
2142	3rg2-C	2.1	4.4	51	613	6	MOLECULE: ENTEROBACTIN SYNTHASE COMPONENT E (ENTE), 2,3-DIH
2143	4tr2-B	2.1	2.9	43	474	9	MOLECULE: SUBTILISIN-LIKE 1 SERINE PROTEASE;
2144	5gko-A	2.1	6.5	70	650	1	MOLECULE: MACROLIDE EXPORT ATP-BINDING/PERMEASE PROTEIN MAC
2145	6s18-A	2.1	4.6	44	143	2	MOLECULE: AROMATIC ACID CHEMORECEPTOR;
2146	5imj-A	2.1	4.1	67	246	7	MOLECULE: CELL DIVISION PROTEIN ZAPD;
2147	3qr0-A	2.1	15.9	81	781	14	MOLECULE: PHOSPHOLIPASE C-BETA (PLC-BETA);
2148	1paq-A	2.1	4.1	61	161	15	MOLECULE: TRANSLATION INITIATION FACTOR EIF-2B EPSILON
2149	6j9r-B	2.1	5.2	41	126	12	MOLECULE: BRAIN TUMOR PROTEIN;
2150	2y3a-B	2.1	9.5	63	236	10	MOLECULE: PHOSPHATIDYLINOSITOL-4,5-BISPHOSPHATE 3-KINASE CA
2151	1zke-D	2.1	4.2	41	83	15	MOLECULE: HYPOTHETICAL PROTEIN HP1531;
2152	7obb-B	2.1	11	78	1130	6	MOLECULE: DNA-DIRECTED RNA POLYMERASE I SUBUNIT RPA1;
2153	3bit-A	2.1	15.8	77	447	5	MOLECULE: FACT COMPLEX SUBUNIT SPT16;
2154	1upt-B	2.1	6	37	58	11	MOLECULE: ADP-RIBOSYLATION FACTOR-LIKE PROTEIN 1;
2155	6ej7-A	2.1	12.6	72	706	3	MOLECULE: XYLOSYLTRANSFERASE 1;
2156	3c8g-C	2.1	4	64	167	8	MOLECULE: PUTATIVE TRANSCRIPTIONAL REGULATOR;
2157	5h9c-A	2.1	4.8	39	78	10	MOLECULE: ENVELOPE GLYCOPROTEIN GP95;
2158	6ghc-B	2.1	14.2	68	286	6	MOLECULE: 5-METHYLCYTOSINE-SPECIFIC RESTRICTION ENZYME A;
2159	5yh1-A	2.1	13.5	65	439	11	MOLECULE: MEMBER OF S1P FAMILY OF RIBOSOMAL PROTEINS;
2160	4qmf-B	2.1	2.6	41	175	15	MOLECULE: KRR1 SMALL SUBUNIT PROCESSOME COMPONENT;
2161	2kjf-A	2.1	2.6	43	60	7	MOLECULE: CARNOCYCLIN-A;
2162	6hs6-A	2.1	7.7	59	69	2	MOLECULE: TYPE VI SECRETION PROTEIN IMPA;
2163	6tdx-H	2.1	10.9	59	160	10	MOLECULE: ATP SYNTHASE F1 SUBUNIT GAMMA;
2164	2hvr-A	2.1	4.6	44	319	16	MOLECULE: 5'-
2165	5whm-C	2.1	5.1	53	264	17	MOLECULE: ICLR FAMILY TRANSCRIPTIONAL REGULATOR;
2166	5mx5-L	2.1	4.7	64	214	8	MOLECULE: PROTEASOME ACTIVATOR COMPLEX SUBUNIT 1;
2167	2mpk-A	2.1	4.2	44	74	7	MOLECULE: CHITIN SYNTHASE 1;

2168	6pwd-A	2.1	12.2	68	176	9	MOLECULE: TYPE III EFFECTOR HOPBF1;
2169	6pl5-B	2.1	3.8	70	577	4	MOLECULE: PEPTIDOGLYCAN GLYCOSYLTRANSFERASE RODA;
2170	6pwn-A	2.1	5.9	53	280	6	MOLECULE: SMALL-CONDUCTANCE MECHANOSENSITIVE CHANNEL;
2171	6fml-G	2.1	9.5	64	731	5	MOLECULE: RUVB-LIKE HELICASE;
2172	7bc6-A	2.1	5	98	432	4	MOLECULE: PROTON-COUPLED FOLATE TRANSPORTER;
2173	6hum-L	2.1	5.4	40	76	5	MOLECULE: NAD(P)H-QUINONE OXIDOREDUCTASE SUBUNIT 1;
2174	3bkx-A	2.1	12.6	58	274	5	MOLECULE: SAM-DEPENDENT METHYLTRANSFERASE;
2175	6orb-A	2.1	39	78	3352	5	MOLECULE: MIDASIN;
2176	3c9p-A	2.1	3.7	50	122	12	MOLECULE: UNCHARACTERIZED PROTEIN SP1917;
2177	6m17-A	2.1	6.5	81	606	1	MOLECULE: SODIUM-DEPENDENT NEUTRAL AMINO ACID TRANSPORTER B
2178	5iy7-M	2.1	14.6	58	310	5	MOLECULE: DNA-DIRECTED RNA POLYMERASE II SUBUNIT RPB1;
2179	4kxr-A	2.1	4.4	43	85	7	MOLECULE: PE25;
2180	6neq-g	2.1	9.1	100	351	13	MOLECULE: 28S RIBOSOMAL RNA, MITOCHONDRIAL;
2181	3bh6-B	2.1	12.1	66	314	11	MOLECULE: ADP-RIBOSYLATION FACTOR-LIKE PROTEIN 3;
2182	4gou-A	2.1	6.4	75	507	9	MOLECULE: EHRGS-RHOGEF;
2183	1snl-A	2.1	10.9	55	99	7	MOLECULE: NUCLEOBINDIN 1;
2184	5uif-A	2.1	3.4	50	112	8	MOLECULE: PS01740;
2185	7pmk-D	2.1	10.9	57	243	5	MOLECULE: DNA REPLICATION LICENSING FACTOR MCM2;
2186	6jy0-A	2.1	7.3	57	410	9	MOLECULE: FLAGELLIN;
2187	4rs7-B	2.1	6	50	88	6	MOLECULE: PARB-C;
2188	1f93-A	2.1	3.2	49	103	4	MOLECULE: DIMERIZATION COFACTOR OF HEPATOCYTE NUCLEAR
2189	5b6b-G	2.1	3.8	42	74	17	MOLECULE: MOB KINASE ACTIVATOR 1B;
2190	7lb8-B	2.1	8.7	82	630	7	MOLECULE: IRON(3+)-HYDROXAMATE IMPORT SYSTEM PERMEASE PROTE
2191	7et3-1	2.1	4.1	71	285	6	MOLECULE: TRIPLEX CAPSID PROTEIN 2;
2192	5wwx-A	2.1	3.4	41	84	10	MOLECULE: RNA-BINDING E3 UBIQUITIN-PROTEIN LIGASE MEX3C;
2193	6z8k-A	2.1	16.4	83	2158	11	MOLECULE: LA CROSSE VIRUS 5' VRNA 1-10;
2194	5x9a-B	2.1	18.6	64	198	6	MOLECULE: CALAXIN;
2195	6x6n-A	2.1	3.5	51	134	8	MOLECULE: OUTER MEMBRANE VIRULENCE PROTEIN YOPE;
2196	2lxe-A	2.1	6.6	51	109	6	MOLECULE: HISTONE-LYSINE N-METHYLTRANSFERASE SUVR4;
2197	2bl8-A	2.1	3.7	50	93	18	MOLECULE: ENTEROCINE A IMMUNITY PROTEIN;
2198	5b7j-A	2.1	5.1	65	110	11	MOLECULE: SWITCH-ACTIVATING PROTEIN 1;
2199	1n93-X	2.1	13.8	76	335	11	MOLECULE: P40 NUCLEOPROTEIN;
2200	6r5k-A	2.1	10.2	68	1040	15	MOLECULE: PAN2-PAN3 DEADENYLATION COMPLEX CATALYTIC SUBUNIT
2201	3urz-B	2.1	4.8	46	206	11	MOLECULE: UNCHARACTERIZED PROTEIN;
2202	4kqt-A	2.1	4.2	55	169	7	MOLECULE: PUTATIVE OUTER MEMBRANE CHAPERONE (OMPH-LIKE);

2203	7am1-B	2.1	6.7	73	811	15	MOLECULE: PROTEIN SSD1;
2204	6w32-B	2.1	8.7	92	288	11	MOLECULE: PHOSPHATIDYLINOSITOL TRANSFER PROTEIN SFH5;
2205	3l0m-A	2.1	6	63	328	10	MOLECULE: DRRA;
2206	3ueb-E	2.1	3.4	47	104	11	MOLECULE: PUTATIVE UNCHARACTERIZED PROTEIN;
2207	4od8-D	2.1	5.7	40	51	13	MOLECULE: URACIL-DNA GLYCOSYLASE;
2208	4rs0-A	2.1	10.9	84	557	12	MOLECULE: PROSTAGLANDIN G/H SYNTHASE 2;
2209	2vwa-A	2.1	3.8	56	99	16	MOLECULE: PUTATIVE UNCHARACTERIZED PROTEIN PF13_0012;
2210	7d5i-A	2.1	7	70	443	9	MOLECULE: CYTOCHROME D UBIQUINOL OXIDASE SUBUNIT 1;
2211	6c26-3	2.1	8.6	58	126	10	MOLECULE: DOLICHYL-DIPHOSPHOOLIGOSACCHARIDE--PROTEIN
2212	6fkg-C	2.1	2.9	49	111	6	MOLECULE: RV1989C (MBCT);
2213	4cpg-A	2.1	5.4	44	69	11	MOLECULE: SMALL GLUTAMINE-RICH TETRATRICOPEPTIDE REPEAT-CON
2214	6gyt-B	2.1	3.5	61	168	5	MOLECULE: HISTONE ACETYLTRANSFERASE P300;
2215	6qq4-A	2.1	3.9	50	121	0	MOLECULE: GENERAL ODORANT-BINDING PROTEIN 28A;
2216	3ezx-A	2.1	4.3	58	212	3	MOLECULE: MONOMETHYLAMINE CORRINOID PROTEIN 1;
2217	3pm2-A	2.1	3.6	58	173	7	MOLECULE: ODORANT BINDING PROTEIN (AGAP007287-PA);
2218	4aqi-A	2.1	3.3	52	96	6	MOLECULE: PROTEIN S100-A7A;
2219	1ypy-A	2.1	3.2	64	182	6	MOLECULE: VIRION MEMBRANE PROTEIN;
2220	6agb-K	2.1	5.4	49	128	8	MOLECULE: CHROMOSOME V, COMPLETE SEQUENCE;
2221	4j8f-A	2.1	6.5	75	551	8	MOLECULE: HEAT SHOCK 70 KDA PROTEIN 1A/1B, HSC70-INTERACTIN
2222	4hh5-A	2.1	3.9	74	163	5	MOLECULE: PUTATIVE TYPE VI SECRETION PROTEIN;
2223	5mss-A	2.1	9.1	85	717	7	MOLECULE: THIOESTER REDUCTASE DOMAIN-CONTAINING PROTEIN;
2224	6qld-N	2.1	13.3	94	371	6	MOLECULE: INNER KINETOCHORE SUBUNIT MIF2;
2225	5xu6-A	2.1	9.8	65	367	5	MOLECULE: INOSITOL-PENTAKISPHOSPHATE 2-KINASE;
2226	6y92-A	2.1	9.4	56	178	9	MOLECULE: B-LYMPHOCYTE ANTIGEN CD20;
2227	7pgs-N	2.1	10.5	78	1787	5	MOLECULE: NEUROFIBROMIN;
2228	7bkb-e	2.1	4.4	72	411	6	MOLECULE: COB--COM HETERODISULFIDE REDUCTASE IRON-SULFUR SU
2229	4kqe-A	2.1	7.3	65	597	11	MOLECULE: GLYCINE--TRNA LIGASE;
2230	2lm9-A	2.1	9.2	51	96	12	MOLECULE: BLO T 21 ALLERGEN;
2231	6pd1-C	2.1	5.4	60	617	5	MOLECULE: NUCLEOTIDYL TRANSFERASE/AMINOTRANSFERASE, CLASS V
2232	2q01-B	2.1	5	51	480	12	MOLECULE: URONATE ISOMERASE;
2233	5i6r-A	2.1	13.2	82	419	6	MOLECULE: SLIT-ROBO RHO GTPASE-ACTIVATING PROTEIN 2;
2234	1x4q-A	2.1	4.7	50	92	8	MOLECULE: U4/U6 SMALL NUCLEAR RIBONUCLEOPROTEIN PRP3;
2235	6l4o-A	2.1	9.7	69	429	4	MOLECULE: APOPTOSIS INHIBITOR 5;
2236	1lxj-A	2.1	3.1	45	104	18	MOLECULE: HYPOTHETICAL 11.5KDA PROTEIN IN HTB2-NTH2 INTERGE
2237	2ibo-A	2.1	2.8	46	90	11	MOLECULE: HYPOTHETICAL PROTEIN SP2199;

2238	1dd3-A	2.1	6.6	49	128	8	MOLECULE: 50S RIBOSOMAL PROTEIN L7/L12;
2239	5t58-D	2.1	7.2	50	201	10	MOLECULE: KLLA0F02343P;
2240	5a2g-A	2.1	15	96	501	5	MOLECULE: CARBOXYLIC ESTER HYDROLASE;
2241	6rd4-X	2.1	10.2	89	542	4	MOLECULE: ASA-10: POLYTOMELLA F-ATP SYNTHASE ASSOCIATED SUB
2242	6ayi-A	2.1	12.2	64	183	6	MOLECULE: HTH-TYPE TRANSCRIPTIONAL REGULATOR UIDR;
2243	1v33-A	2.1	10.8	66	346	8	MOLECULE: DNA PRIMASE SMALL SUBUNIT;
2244	6tm2-A	2.1	4.2	79	708	6	MOLECULE: MUCIN-2;
2245	1i5p-A	2.1	20.9	89	633	8	MOLECULE: PESTICIDIAL CRYSTAL PROTEIN CRY2AA;
2246	6yxu-H	2.1	12.9	71	691	8	MOLECULE: DNA-DIRECTED RNA POLYMERASE SUBUNIT ALPHA;
2247	2d9d-A	2.1	9.2	54	89	4	MOLECULE: BAG FAMILY MOLECULAR CHAPERONE REGULATOR 5;
2248	4acl-A	2.1	5	50	155	14	MOLECULE: TSSL;
2249	6j52-A	2.1	9.6	67	94	12	MOLECULE: CASPASE RECRUITMENT DOMAIN-ONLY PROTEIN;
2250	7emf-K	2	8.4	61	112	5	MOLECULE: MEDIATOR OF RNA POLYMERASE II TRANSCRIPTION SUBUN
2251	1x9b-A	2	8.5	42	53	7	MOLECULE: HYPOTHETICAL MEMBRANE PROTEIN TA0354_69_121;
2252	6kn5-A	2	4.3	51	225	10	MOLECULE: AF4/FMR2 FAMILY MEMBER 4;
2253	6bwi-A	2	13	71	816	11	MOLECULE: TRANSIENT RECEPTOR POTENTIAL CATION CHANNEL SUBFA
2254	6ijf-A	2	3	49	101	6	MOLECULE: TAI4;
2255	3zhe-A	2	17.4	70	399	4	MOLECULE: NONSENSE-MEDIATED MRNA DECAY PROTEIN;
2256	2roe-A	2	2.5	39	66	10	MOLECULE: HEAVY METAL BINDING PROTEIN;
2257	6ras-I	2	16.3	59	433	8	MOLECULE: DNA (5'-
2258	4g6d-B	2	8.7	60	198	5	MOLECULE: RNA POLYMERASE SIGMA FACTOR RPOD;
2259	6s6v-A	2	11.8	51	386	16	MOLECULE: NUCLEASE SBCCD SUBUNIT D;
2260	3jcm-B	2	15.3	80	428	6	MOLECULE: PRE-MRNA-SPLICING FACTOR 8;
2261	5wjt-A	2	6.1	77	302	5	MOLECULE: FLAGELLIN;
2262	5zdh-A	2	12.4	88	497	10	MOLECULE: TYPE II SECRETION SYSTEM PROTEIN D;
2263	6d5f-a	2	8.5	56	131	11	MOLECULE: FIMBRIAL PROTEIN;
2264	4noo-A	2	3.4	61	200	7	MOLECULE: VGRG PROTEIN;
2265	6nf1-A	2	9.1	80	550	4	MOLECULE: PROTO-ONCOGENE VAV;
2266	6wu0-B	2	18.2	83	854	4	MOLECULE: HOPANOID BIOSYNTHESIS ASSOCIATED RND TRANSPORTER
2267	5z7c-A	2	7.6	82	414	9	MOLECULE: 3'3'-CGAMP-SPECIFIC PHOSPHODIESTERASE 3;
2268	2k3o-A	2	3.6	64	129	9	MOLECULE: TUSP1;
2269	5ok8-B	2	5.6	50	115	2	MOLECULE: LPP20 LIPOPROTEIN;
2270	2rd9-A	2	9.8	77	189	6	MOLECULE: BH0186 PROTEIN;
2271	5u9m-D	2	4.3	49	234	4	MOLECULE: SUPEROXIDE DISMUTASE [CU-ZN];
2272	1win-A	2	2.5	50	143	20	MOLECULE: FLOTILLIN 2;

2273	4igg-B	2	5.2	81	771	9	MOLECULE: CATENIN ALPHA-1;
2274	5eom-C	2	4	72	353	11	MOLECULE: PROTEIN MAB-21-LIKE 1;
2275	4ycz-A	2	4.8	68	713	4	MOLECULE: FUSION PROTEIN OF SEC13 AND NUP145C;
2276	4kf8-A	2	12.9	77	344	3	MOLECULE: NUP188;
2277	3k1z-A	2	20.4	78	241	8	MOLECULE: HALOACID DEHALOGENASE-LIKE HYDROLASE DOMAIN-CONTA
2278	2fu2-A	2	3.1	45	78	18	MOLECULE: HYPOTHETICAL PROTEIN SPY2152;
2279	5v8z-C	2	5.6	50	98	10	MOLECULE: ENDOPLASMIC RETICULUM RESIDENT PROTEIN 29;
2280	5da9-C	2	6.2	50	85	6	MOLECULE: PUTATIVE UNCHARACTERIZED PROTEIN,PUTATIVE UNCHARA
2281	7pmk-X	2	13.2	83	665	8	MOLECULE: DNA REPLICATION LICENSING FACTOR MCM2;
2282	1k99-A	2	13.5	45	91	9	MOLECULE: UPSTREAM BINDING FACTOR 1;
2283	6x0l-P	2	19.6	84	474	11	MOLECULE: HISTONE PARYLATION FACTOR 1;
2284	6lul-A	2	5.4	52	223	2	MOLECULE: RESPIRATORY SUPERCOMPLEX FACTOR 2, MITOCHONDRIAL;
2285	6w17-G	2	3.6	72	150	8	MOLECULE: ACTIN-RELATED PROTEIN 3;
2286	7o3x-B	2	9.9	66	215	12	MOLECULE: PROTEIN SLL0617;
2287	1u8s-B	2	11.6	58	172	5	MOLECULE: GLYCINE CLEAVAGE SYSTEM TRANSCRIPTIONAL
2288	3cxj-A	2	9.6	67	144	7	MOLECULE: UNCHARACTERIZED PROTEIN;
2289	6xfl-A	2	9.2	52	79	10	MOLECULE: TYPE 3 SECRETION SYSTEM PILOTIN;
2290	7ar9-X	2	3.2	45	99	4	MOLECULE: ND3;
2291	5x4j-A	2	2.2	40	471	8	MOLECULE: UNCHARACTERIZED PROTEIN;
2292	1uus-A	2	8.7	99	465	6	MOLECULE: STAT PROTEIN;
2293	3zta-A	2	4.4	58	139	12	MOLECULE: ANTI-SIGMA-FACTOR ANTAGONIST (STAS) DOMAIN PROTEI
2294	6nt7-A	2	16.4	54	294	0	MOLECULE: STIMULATOR OF INTERFERON GENES PROTEIN;
2295	5c00-A	2	5.1	55	202	4	MOLECULE: MDBA PROTEIN;
2296	2l22-A	2	10.4	63	183	8	MOLECULE: MUPIROCIN DIDOMAIN ACYL CARRIER PROTEIN;
2297	2ets-A	2	4.9	50	115	8	MOLECULE: HYPOTHETICAL PROTEIN;
2298	2q0o-D	2	6.1	48	87	8	MOLECULE: PROBABLE TRANSCRIPTIONAL ACTIVATOR PROTEIN TRAR;
2299	4p6v-B	2	6.4	66	348	6	MOLECULE: NA(+)-TRANSLOCATING NADH-QUINONE REDUCTASE SUBUNI
2300	6giy-G	2	7.1	67	444	6	MOLECULE: TSSF;
2301	1mqs-A	2	11.3	86	588	13	MOLECULE: SLY1 PROTEIN;
2302	3cit-A	2	4.8	50	155	12	MOLECULE: SENSOR HISTIDINE KINASE;
2303	2wop-A	2	12.7	71	555	7	MOLECULE: CLAVULANIC ACID BIOSYNTHESIS OLIGOPEPTIDE
2304	5gai-K	2	3.5	51	146	14	MOLECULE: PORTAL PROTEIN;
2305	1urf-A	2	6.1	50	81	12	MOLECULE: PROTEIN KINASE C-LIKE 1;
2306	4ng2-E	2	5.9	50	94	4	MOLECULE: TRANSCRIPTIONAL ACTIVATOR PROTEIN LASR;
2307	5ehk-A	2	12.5	68	1036	10	MOLECULE: LANTIBIOTIC DEHYDRATASE;

2308	6fqb-A	2	6.2	73	383	4	MOLECULE: MUR LIGASE FAMILY PROTEIN;
2309	6q0x-A	2	9.7	65	373	11	MOLECULE: SORTING NEXIN MVP1;
2310	3veb-B	2	9.9	59	150	5	MOLECULE: MACRODOMAIN TER PROTEIN;
2311	5aa5-C	2	9.9	69	561	7	MOLECULE: NIFE-HYDROGENASE SMALL SUBUNIT, HOFK;
2312	6f5d-G	2	4.8	56	277	7	MOLECULE: ATP SYNTHASE ALPHA CHAIN, MITOCHONDRIAL;
2313	3ce9-A	2	6.9	100	349	6	MOLECULE: GLYCEROL DEHYDROGENASE;
2314	5e8j-A	2	6.2	56	311	13	MOLECULE: MRNA CAP GUANINE-N7 METHYLTRANSFERASE;
2315	2jmh-A	2	5.6	56	117	9	MOLECULE: MITE ALLERGEN BLO T 5;
2316	5yz0-C	2	5.6	91	362	9	MOLECULE: SERINE/THREONINE-PROTEIN KINASE ATR;
2317	6nps-A	2	5.7	88	968	10	MOLECULE: AXYAGU115A;
2318	7clv-A	2	8.2	76	222	4	MOLECULE: TIM23 ISOFORM 1;
2319	6gdj-A	2	4.1	38	71	5	MOLECULE: MTO2;
2320	6rj9-C	2	18.9	74	836	3	MOLECULE: ACRIIA6;
2321	5v2o-A	2	3.8	44	60	9	MOLECULE: TP2;
2322	2yn7-A	2	6	77	214	17	MOLECULE: OUTER SURFACE PROTEIN;
2323	6oit-F	2	4.2	40	342	13	MOLECULE: PROTEIN RDM1;
2324	2rp4-A	2	5.7	44	71	11	MOLECULE: TRANSCRIPTION FACTOR P53;
2325	4rbr-A	2	2.5	43	131	14	MOLECULE: HTH-TYPE TRANSCRIPTIONAL REGULATOR ROT;
2326	6lw5-A	2	5.1	75	427	12	MOLECULE: SOLUBLE CYTOCHROME B562,N-FORMYL PEPTIDE RECEPTOR
2327	2pzi-A	2	10.4	91	654	8	MOLECULE: PROBABLE SERINE/THREONINE-PROTEIN KINASE PKNG;
2328	5xtc-j	2	11.6	56	115	5	MOLECULE: NADH DEHYDROGENASE [UBIQUINONE] IRON-SULFUR PROTE
2329	3av0-B	2	9.8	63	365	6	MOLECULE: DNA DOUBLE-STRAND BREAK REPAIR PROTEIN MRE11;
2330	5jjh-A	2	11.9	87	704	6	MOLECULE: 4-ALPHA-GLUCANOTRANSFERASE;
2331	4zcf-A	2	7.7	72	616	8	MOLECULE: RESTRICTION ENDONUCLEASE ECOP15I, MODIFICATION SU
2332	5nik-D	2	4.1	48	340	19	MOLECULE: OUTER MEMBRANE PROTEIN TOLC;
2333	5a7d-R	2	10.5	81	311	14	MOLECULE: PINS;
2334	1te2-A	2	8.1	73	218	4	MOLECULE: 2-DEOXYGLUCOSE-6-P PHOSPHATASE;
2335	4cej-B	2	16.2	97	1156	6	MOLECULE: ATP-DEPENDENT HELICASE/NUCLEASE SUBUNIT A;
2336	5j1s-A	2	9.9	68	277	12	MOLECULE: TORSIN-1A;
2337	6g2a-A	2	10.8	73	361	4	MOLECULE: [PROTEIN ADP-RIBOSYLARGININE] HYDROLASE;
2338	6f1y-j	2	10.5	53	303	6	MOLECULE: CYTOPLASMIC DYNEIN 1 HEAVY CHAIN 1,DYNEIN HEAVY C
2339	3din-E	2	5.6	43	65	9	MOLECULE: PROTEIN TRANSLOCASE SUBUNIT SECA;
2340	1in0-A	2	4.8	67	162	7	MOLECULE: YAJQ PROTEIN;
2341	6x0u-D	2	6.1	60	158	15	MOLECULE: GAMMA-TUBULIN COMPLEX COMPONENT 3;
2342	2oeb-A	2	5.1	57	152	12	MOLECULE: HYPOTHETICAL PROTEIN;

2343	6iqs-C	2	7.3	64	541	5	MOLECULE: LIPOPROTEIN NLPI;
2344	7pmk-Y	2	6	57	98	16	MOLECULE: DNA REPLICATION LICENSING FACTOR MCM2;
2345	3qnq-D	2	10.5	75	436	5	MOLECULE: PTS SYSTEM, CELLOBIOSE-SPECIFIC IIC COMPONENT;
2346	2m5o-A	2	15.1	56	97	7	MOLECULE: NFU1 IRON-SULFUR CLUSTER SCAFFOLD HOMOLOG, MITOCH
2347	6j6g-C	2	17.2	95	920	11	MOLECULE: PRE-MRNA-SPLICING FACTOR 8;
2348	5a1v-Q	2	11.4	71	292	6	MOLECULE: ADP-RIBOSYLATION FACTOR 1;
2349	5ame-A	2	2.9	42	123	2	MOLECULE: BROMODOMAIN-CONTAINING PROTEIN 1;
2350	3bf5-A	2	10.3	53	284	8	MOLECULE: RIBOKINASE RELATED PROTEIN;
2351	2kg7-B	2	6.7	53	97	2	MOLECULE: UNCHARACTERIZED PROTEIN ESXG (PE FAMILY PROTEIN);
2352	5xjt-2	2	6.1	50	217	12	MOLECULE: GEM-ASSOCIATED PROTEIN 2;
2353	6u0o-B	2	7.4	66	288	8	MOLECULE: LYSOSTAPHIN RESISTANCE PROTEIN A;
2354	5l4k-V	2	9.3	67	293	7	MOLECULE: 26S PROTEASOME NON-ATPASE REGULATORY SUBUNIT 4;
2355	2lyy-A	2	3.3	50	96	6	MOLECULE: UNCHARACTERIZED PROTEIN;
2356	2iaz-B	2	15	55	113	5	MOLECULE: HYPOTHETICAL PROTEIN SP1372;
2357	5a63-D	2	7.9	54	100	4	MOLECULE: NICASTRIN;
2358	2kna-A	2	5.4	51	104	4	MOLECULE: BACULOVIRAL IAP REPEAT-CONTAINING PROTEIN 4;
2359	1khm-A	2	3.4	43	89	12	MOLECULE: PROTEIN (HNRNP K);
2360	5n9q-A	2	14	72	128	4	MOLECULE: INACTIVE POLY [ADP-RIBOSE] POLYMERASE RCD1;
2361	6fpd-A	2	6.3	50	191	10	MOLECULE: PROTEIN AB21;
2362	5ldi-A	2	17.2	55	175	11	MOLECULE: RNA 2',3'-CYCLIC PHOSPHODIESTERASE;
2363	6xrx-A	2	12.5	88	551	7	MOLECULE: MALTOSE/MALTODEXTRIN-BINDING PERIPLASMIC PROTEIN,
2364	5f1q-A	2	12.8	71	515	7	MOLECULE: PERIPLASMIC DIPEPTIDE TRANSPORT PROTEIN;
2365	6v85-A	2	16.1	76	1901	3	MOLECULE: RNA-DIRECTED RNA POLYMERASE L;
2366	1ss3-A	2	13.9	45	50	9	MOLECULE: POLLEN ALLERGEN OLE E 6;
2367	2pmy-A	2	3.1	39	73	10	MOLECULE: RAS AND EF-HAND DOMAIN-CONTAINING PROTEIN;
2368	7bfq-B	2	6.7	60	590	0	MOLECULE: INTEGRATOR COMPLEX SUBUNIT 9;
2369	4bi8-A	2	4.3	49	94	8	MOLECULE: RAP1A;
2370	1r9q-A	2	3.1	49	310	4	MOLECULE: GLYCINE BETAINE-BINDING PERIPLASMIC PROTEIN;
2371	2lu1-A	2	3.3	43	89	14	MOLECULE: SUBTILASE;
2372	2r10-B	2	12.9	61	298	11	MOLECULE: CHROMATIN STRUCTURE-REMODELING COMPLEX PROTEIN
2373	1yvw-A	2	5.8	57	92	4	MOLECULE: PHOSPHORIBOSYL-ATP PYROPHOSPHATASE;
2374	7ktr-A	2	10	98	3042	7	MOLECULE: TRANSFORMATION/TRANSCRIPTION DOMAIN-ASSOCIATED PR
2375	6ulg-G	2	12.1	59	310	7	MOLECULE: FOLLICULIN;
2376	3t57-A	2	14.3	52	288	8	MOLECULE: UDP-N-ACETYLGLUCOSAMINE O-ACYLTRANSFERASE DOMAIN-
2377	7b2b-A	2	3.9	48	104	10	MOLECULE: AMINO ACID ADENYLATION DOMAIN-CONTAINING PROTEIN;

2378	7nvr-3	2	4.5	42	214	12	MOLECULE: TFIIH BASAL TRANSCRIPTION FACTOR COMPLEX HELICASE
2379	3d6j-A	2	7.3	59	210	7	MOLECULE: PUTATIVE HALOACID DEHALOGENASE-LIKE HYDROLASE;
2380	5uxg-A	2	4.5	76	212	3	MOLECULE: ALDEHYDE DEFORMYLATING OXYGENASE;
2381	6n9j-B	2	11.8	50	356	4	MOLECULE: CLOSTRIPAIN-RELATED PROTEIN;
2382	6swy-9	2	8.2	48	157	0	MOLECULE: VACUOLAR IMPORT AND DEGRADATION PROTEIN 28;
2383	3nyb-A	2	11.2	72	323	7	MOLECULE: POLY(A) RNA POLYMERASE PROTEIN 2;
2384	6nr8-3	2	4.2	44	132	11	MOLECULE: PREFOLDIN SUBUNIT 1;
2385	3kkz-A	2	10	69	257	6	MOLECULE: UNCHARACTERIZED PROTEIN Q5LES9;
2386	1gak-A	2	4.7	57	137	11	MOLECULE: FERTILIZATION PROTEIN;
2387	3bqp-B	2	2.5	47	80	11	MOLECULE: PROACTIVATOR POLYPEPTIDE;
2388	1oao-A	2	7	68	673	6	MOLECULE: CARBON MONOXIDE DEHYDROGENASE/ACETYL-COA
2389	1ijl-B	2	4.4	59	123	10	MOLECULE: PHOSPHOLIPASE A2;
2390	5ux5-A	2	12.3	60	963	13	MOLECULE: BIFUNCTIONAL PROTEIN PROLINE UTILIZATION A (PUTA)
2391	1h21-A	2	8.2	64	240	8	MOLECULE: SPLIT-SORET CYTOCHROME C;
2392	3hi0-A	2	12	88	501	9	MOLECULE: PUTATIVE EXOPOLYPHOSPHATASE;
2393	6s3f-A	2	3.5	43	65	14	MOLECULE: 2S ALBUMIN;
2394	6mn5-D	2	13.5	65	260	8	MOLECULE: AMINOGLYCOSIDE N(3)-ACETYLTRANSFERASE, AAC(3)-IVA
2395	1q1v-A	2	5.6	49	70	12	MOLECULE: DEK PROTEIN;
2396	1vzs-A	2	10.6	46	76	11	MOLECULE: ATP SYNTHASE COUPLING FACTOR 6, MITOCHONDRIAL
2397	5d18-A	2	11	76	202	11	MOLECULE: TETR FAMILY TRANSCRIPTIONAL REGULATOR;
2398	7kah-D	2	6.6	52	493	10	MOLECULE: PROTEIN TRANSPORT PROTEIN SEC61;
2399	1e7d-A	2	2.9	54	157	4	MOLECULE: RECOMBINATION ENDONUCLEASE VII;
2400	1wte-A	2	3.6	56	272	4	MOLECULE: 5'-D(*AP*CP*CP*GP*GP*GP*CP*CP*CP*TP*GP*CP*C)-3';
2401	5but-I	2	7.3	68	431	7	MOLECULE: KTR SYSTEM POTASSIUM UPTAKE PROTEIN A,KTR SYSTEM
2402	3s63-B	2	4.3	43	90	9	MOLECULE: SAPOSIN-LIKE PROTEIN;
2403	1eyy-A	2	7.3	70	504	10	MOLECULE: ALDEHYDE DEHYDROGENASE;
2404	7ctp-A	2	4.4	65	553	11	MOLECULE: PROTEIN NIBAN 2;
2405	5z08-D	2	5.5	36	44	8	MOLECULE: CENP-I;
2406	5g4d-A	2	3.4	45	79	7	MOLECULE: CRISPR-ASSOCIATED ENDORIBONUCLEASE CAS2;
2407	4wbt-A	2	17.5	83	369	4	MOLECULE: PROBABLE HISTIDINOL-PHOSPHATE AMINOTRANSFERASE;
2408	5fhp-C	2	10.8	73	209	7	MOLECULE: NICR;
2409	6gyb-C	2	7.7	53	205	6	MOLECULE: VIRB7;
2410	7e1w-O	2	10.7	71	223	3	MOLECULE: CYTOCHROME C OXIDASE SUBUNIT 2;
2411	1n1c-A	2	8.2	68	199	4	MOLECULE: TORA SPECIFIC CHAPERONE;
2412	7bk0-a	2	14.2	45	250	16	MOLECULE: FLAGELLAR M-RING PROTEIN;

2413	1gmu-C	2	11.1	58	140	3	MOLECULE: UREE;
2414	3dee-A	2	12.4	50	200	8	MOLECULE: PUTATIVE REGULATORY PROTEIN;
2415	4od4-A	2	5.1	70	275	7	MOLECULE: 4-HYDROXYBENZOATE OCTAPRENYLTRANSFERASE;
2416	7e40-D	2	10	95	345	12	MOLECULE: PROTEIN PHOSPHATE STARVATION RESPONSE 2;

S2 Table. Worm strains used in this study

Strain name	Genotype (transgene or mutant alelle details)	Figures	Source	Notes
ERT054	*jyIs8[pals-5p::gfp, myo-2p::mCherry] X*	4	Bakowski et al., 2012 [1]	
ERT1053	*mgTi54[rpl-28p::mScarlet::drh-1]; drh-1(jy110) IV*	5A/B, S5	Mao et al., 2020 [2]	GR3325 from Mao et al., 2020 was crossed with *drh-1(jy110)* in this
ERT1076	*jyEx302[vha-6p::drh-1-NTD::wrmScarlet::3xFLAG::unc-54 3'UTR, myo-3p::mCherry]; rde-1(ne219) V; jyIs8 X*	1C-E, S3B	This study	
ERT1151	*mgTi54[rpl-28p::mScarlet::drh-1]; drh-1(jy110) IV; rde-1(ne219) V*	5C	Mao et al., 2020 [2]	
ERT1182	*jyEx305[vha-6p::drh-1-NTD::wrmScarlet::3xFLAG::unc-54 3'UTR]; rde-1(ne219) V; jyIs8 X*	1C/D, 2A/B, S1B	This study	
ERT1199	*jyEx305; rde-1(ne219) V*	1F, 3A/B	This study	
ERT1205	*jyEx305; zip-1(jy14) III; rde-1(ne219) V; jyIs8 X*	2A/B	This study	
ERT1207	*jyEx304[vha-6p::drh-1-NTD::wrmScarlet::3xFLAG::unc-54 3'UTR]; rde-1(ne219) V; jyIs8 X*	1C/D	This study	
ERT1208	*jyEx304; rde-1(ne219) V*	3A-E, S3A, S4	This study	
ERT1209	*jyEx302; rde-1(ne219) V*	3A/B, S3C	This study	
ERT1215	*frSi17[mtl-2p::rde-1 3'UTR] II; rde-1(ne300) V; jyIs8 X*	4	Watts et al., 2020 [3]	IG1839 from Watts et al., 2020 was crossed with ERT054 in this study
ERT1216	*frSi21[col-62p::rde-1 3'UTR] II; rde-1(ne300) V; jyIs8 X*	4	Watts et al., 2020 [3]	IG1846 from Watts et al., 2020 was crossed with ERT054 in this study
ERT1257	*jyEx305; zip-1::gfp(jy132) III; rde-1(ne219) V*	S2	This study	
ERT1262	*jyEx336[vha-6p::wrmScarlet::3xFLAG 3'UTR]; rde-1(ne219) V; jyIs8 X*	S1A	This study	
ERT711	*rde-1(ne219) V; jyIs8 X*	1C/D, 2A/B, S1B	This study	
ERT781	*drh-1(jy110)*	S5	Sowa et al., 2020 [4]	
GR3325	*mgTi54[rpl-28p::mScarlet::drh-1]*	S5	Mao et al., 2020 [2]	
N2	wild type	S5	Caenorhabditis Genetics	
WM27	*rde-1(ne219) V*	1F	Caenorhabditis Genetics	

References

[1] M. A. Bakowski, *et al.* , Ubiquitin-Mediated Response to Microsporidia and Virus Infection in C. elegans. *PLOS Pathogens* **10**, e1004200 (2014).
[2] K. Mao, P. Breen, G. Ruvkun, Mitochondrial dysfunction induces RNA interference in C. elegans through a pathway homologous to the mammalian RIG-I antiviral response. *PLoS Biol* **18**, e3000996 (2020).
[3] J. S. Watts, *et al.* , New Strains for Tissue-Specific RNAi Studies in Caenorhabditis elegans. *G3 (Bethesda)* **10**, 4167–4176 (2020).
[4] J. N. Sowa, *et al.* , The Caenorhabditis elegans RIG-I Homolog DRH-1 Mediates the Intracellular Pathogen Response upon Viral Infection. *J Virol* **94**, e01173-19 (2020).

S3 Table. Constructs used in this study

Construct name	Description	Source
pET636	*vha-6p::pals-22 cDNA no stop::gfp_SBP_3xFLAG::unc-54 3'UTR* in pCFJ151	[1]
pET770	*vha-6p::drh-1-NTD::wrmScarlet::3xFLAG::unc-54 3'UTR, unc-119(+)*	This study
pET786	*vha-6p::wrmScarlet::3xFLAG::unc-54 3'UTR, unc-119(+)*	This study
pET788	*vha-6p::drh-1::wrmScarlet::3XFLAG::unc-54 3'UTR, unc-119(+)*	This study
Tian233	*rpl-28p::mScarlet::drh-1::unc-119(+), Mos1*	[2]

References

[1] K. C. Reddy, *et al.* , An Intracellular Pathogen Response Pathway Promotes Proteostasis in C. elegans. *Curr Biol* **27**, 3544-3553.e5 (2017).

[2] K. Mao, P. Breen, G. Ruvkun, Mitochondrial dysfunction induces RNA interference in C. elegans through a pathway homologous to the mammalian RIG-I antiviral response. *PLoS Biol* **18**, e3000996 (2020).

S4 Table. Primers used in this study

Primer name	Sequence	Purpose
LEB001	TACAAATCAGGATCAGGATCAGC	Cloning for intestinal expression of *drh-1(2CARD)*
LEB002	CCCGGGTTTATGGGTTTTGG	Cloning for intestinal expression of *drh-1(2CARD)*
LEB003	ATGGTCAGCAAGGGAGAGG	Cloning for empty vector control
LEB004	CCCGGGTTTATGGGTTTTGG	Cloning for empty vector control
LEB005	TTCAGGGCGACTAAAACCTACCAAAACCCATAAACCCGGGAGGAAAAAGCAGTGTTCTTC	Cloning for intestinal expression of *drh-1*
LEB006	GAACTCCTTGATAACTGCCTCTCCCTTGCTGACCATTGCTTCTCTGATTAAATTGACTAC	Cloning for intestinal expression of *drh-1*
snb-1 F	CCGGATAAGACCATCTTGACG	qPCR
snb-1 R	GACGACTTCATCAACCTGAGC	qPCR
pals-5 F	CATTGGAAAGCGATATTGGA	qPCR
pals-5 R	TCTCCAGGCACCTATCTTGTAG	qPCR
F26F2.1 F	TGGAACCAGGTCAGAGACAC	qPCR
F26F2.1 R	TTGTGAGAATTTCCGCGATA	qPCR
skr-5 F	CGAAGAGCAAGATGTCAAAATTG	qPCR
skr-5 R	AGAAGCTTGGATTGATTGGCA	qPCR
cul-6 F	CTGGGCTTACTCACAATGCC	qPCR
cul-6 R	GCAGAGTTGGCTTGCTGTAA	qPCR
eol-1 F	GAAGGAGGTGGCGATGTTTAT	qPCR
eol-1 R	CGGCGTCGATTGTCTCTTT	qPCR
RNA1 F	ACCTCACAACTGCCATCTACA	qPCR
RNA1 R	GACGCTTCCAAGATTGGTATTGGT	qPCR
