# Supplementary material for: C. elegans RIG-I-like receptor DRH-1 signals via CARDs to activate anti-viral immunity in intestinal cells
Source: bioRxiv. 2024 Feb 8:2024.02.05.578694. Preprint. [Version 1] doi: 10.1101/2024.02.05.578694 (PMC10871272; doi:10.1101/2024.02.05.578694)
Supplement: 2 [file NIHPP2024.02.05.578694V1-supplement-1.pdf]

bioRxiv preprint doi: https://doi.org/10.1101/2024.02.05.578694; this version posted February 8, 2024. The copyright holder for this preprint (which was not certified by peer review) is the author/funder, who has granted bioRxiv a license to display the preprint in perpetuity. It is made available under aCC-BY 4.0 International license.

Supporting Information

S1 Fig. DRH-1(2CARD) specifically induces *pals-5p::*GFP*.* (A) Representative images showing *pals-5p::*GFP induction in DRH-1(2CARD) transgenic animals (line *jyEx305*) and absence of *pals-5p*::GFP induction in empty vector control (line *jyEx336*). *myo-2p::*mCherry is a part of the *jyIs8[pals-5p::gfp]* transgene and is constitutively expressed in the pharynx. Scale bar = 50 μm. (B) qRT-PCR analysis of DRH-1(2CARD) line *jyEx305* (expresses *pals-5p::*GFP

bioRxiv preprint doi: https://doi.org/10.1101/2024.02.05.578694; this version posted February 8, 2024. The copyright holder for this preprint (which was not certified by peer review) is the author/funder, who has granted bioRxiv a license to display the preprint in perpetuity. It is made available under aCC-BY 4.0 International license.

reporter) shown in (A). RNA was extracted from a mixed-stage population containing both DRH-1(2CARD) transgenic animals and their non-transgenic siblings. Fold change in gene expression was determined relative to a non-transgenic control strain (*rde-1* mutant in a *pals-5p::*GFP background). Bars represent the mean across experimental replicates; error bars represent the standard deviation. Each dot represents a biological replicate (a plate with a minimum of 2000 animals); four independent experimental replicates were performed. A one-tailed *t*-test was used to calculate p-values; *p < 0.05.

S2 Fig. DRH-1(2CARD) expression does not promote nuclear localization of ZIP-1::GFP. Representative images showing ZIP-1::GFP expression in DRH-1(2CARD) transgenic animals and non-transgenic siblings. ZIP-1::GFP is not visible in the nuclei of DRH-1(2CARD) animals. Bortezomib treatment was used as a positive control for nuclear localization of ZIP-1::GFP. White arrowheads indicate ZIP-1::GFP expression in the nucleus. Yellow arrowheads indicate autofluorescence from intestinal gut granules. Scale bar = 25 μm. (B) ZIP-1::GFP is not present in intestinal nuclei of untreated animals, but is expressed in 100% of animals treated with bortezomib. Localization pattern of ZIP-1::GFP is the same for both DRH-1(2CARD) animals and non-transgenic siblings. For each genotype and treatment, 45 total animals were scored. Bars represents the mean across biological replicates; error bars represent the standard deviation. Each dot represents a biological replicate (a plate with 15 animals).

S3 Fig. DRH-1(2CARD) expression does not reduce *N. parisii* pathogen load and delays development. (A) Quantification of *N. parisii* pathogen load in individual experiments (combined results shown in Fig. 3D) by FISH using fluorescein-conjugated (green) probes that target *N. parisii* ribosomal RNA. A COPAS Biosort instrument was used to measure green fluorescence normalized to time-of-flight (a measure of worm size). Dots represent individual animals. Left (R1): n = 353 (non-transgenic; uninfected), 317 (non-transgenic; infected), 791 (DRH-1(2CARD); uninfected), or 535 (DRH-1(2CARD); infected). Center (R2): n = 336 (non-transgenic; uninfected), 337 (non-transgenic; infected), 260 (DRH-1(2CARD); uninfected), or 385 (DRH-1(2CARD); infected). Right (R3): n = 322 (non-transgenic; uninfected), 271 (non-transgenic; infected), 384 (DRH-1(2CARD); uninfected), or 372 (DRH-1(2CARD); infected). Horizontal lines in box-and-whisker plots represent median values, and the box reflects the 25th to 75th percentiles. Each panel displays data from an independent experimental replicate. A Mann-Whitney *U* test was used to calculate p-values; ****p < 0.0001. (B) DRH-1(2CARD) animals exhibit delayed development relative to non-transgenic siblings in a strain background with (B) or without (C) the *pals-5p::*GFP

bioRxiv preprint doi: https://doi.org/10.1101/2024.02.05.578694; this version posted February 8, 2024. The copyright holder for this preprint (which was not certified by peer review) is the author/funder, who has granted bioRxiv a license to display the preprint in perpetuity. It is made available under aCC-BY 4.0 International license.

reporter. Bars represent the mean across experimental replicates; error bars represent the standard deviation. A two-tailed *t*-test was used to calculate p-values; **p < 0.01. (D) Intestinal overexpression of full-length DRH-1 leads to developmental arrest at L1/L2 larval stages in 100% of transgenic progeny across four injections. The fraction of transgenic progeny exhibiting larval arrest is displayed above each bar.

S4 Fig. Uninfected intestinal cells predominantly exhibit DRH-1(2CARD) aggregates, whereas virus-infected cells are enriched for DRH-1(2CARD) puncta. In the absence of infection, all animals exhibit DRH-1(2CARD) aggregates in the intestinal cytoplasm (white box), with some animals exhibiting both DRH-1(2CARD) aggregates and puncta (white arrowhead). In infected animals, the majority of virus-infected cells (green FISH staining) contain DRH-1(2CARD) puncta only, with some animals exhibiting both puncta and aggregates. In the same virus-infected animals, most uninfected neighboring cells (no green FISH staining) show DRH-1(2CARD) aggregates. Intestine is outlined by white dashed line. Proportions determined by scoring 38 (uninfected) or 36 (virus-infected) animals across three independent experimental replicates.

S5 Fig. Expression of the *rpl-28p::mScarlet::drh-1* transgene rescues *pals-5* expression and Orsay RNA1 levels upon viral infection. (A) During viral infection, expression of the mScarlet::DRH-1 transgene (*mgTi54)* in a *drh-1(jy110)* deletion mutant is sufficient to rescue mRNA levels of (A) IPR genes *F26F2.1* and *pals*-5, as well as (B) Orsay RNA1 to WT levels. qRT-PCR analysis of *F26F2.1*, *pals-5*, and Orsay RNA1 in WT animals (N2), a *drh-1(jy110)* deletion mutant, and animals that express the *mgTi54* transgene in a *drh-1(jy110)* or WT background. Fold change in gene expression was determined relative to the uninfected WT (N2) control. Bars represent the mean; error bars represent the standard deviation. Each dot represents a biological replicate (a plate with 2000 animals); three independent experimental replicates were performed. A one-sample Wilcoxon signed rank test was used to compare the distribution of values against a hypothetical value of 1 in the uninfected group. A Mann-Whitney *U* test was used to calculate p-values for comparisons between samples in the infected group; *p < 0.05.

bioRxiv preprint doi: https://doi.org/10.1101/2024.02.05.578694; this version posted February 8, 2024. The copyright holder for this preprint (which was not certified by peer review) is the author/funder, who has granted bioRxiv a license to display the preprint in perpetuity. It is made available under aCC-BY 4.0 International license.

Supplementary Tables – See supporting information

S1 Table. Foldseek and Dali analyses.

S2 Table. List of strains used in this study.

S3 Table. Constructs used in this study.

S4 Table. Primers used in this study.

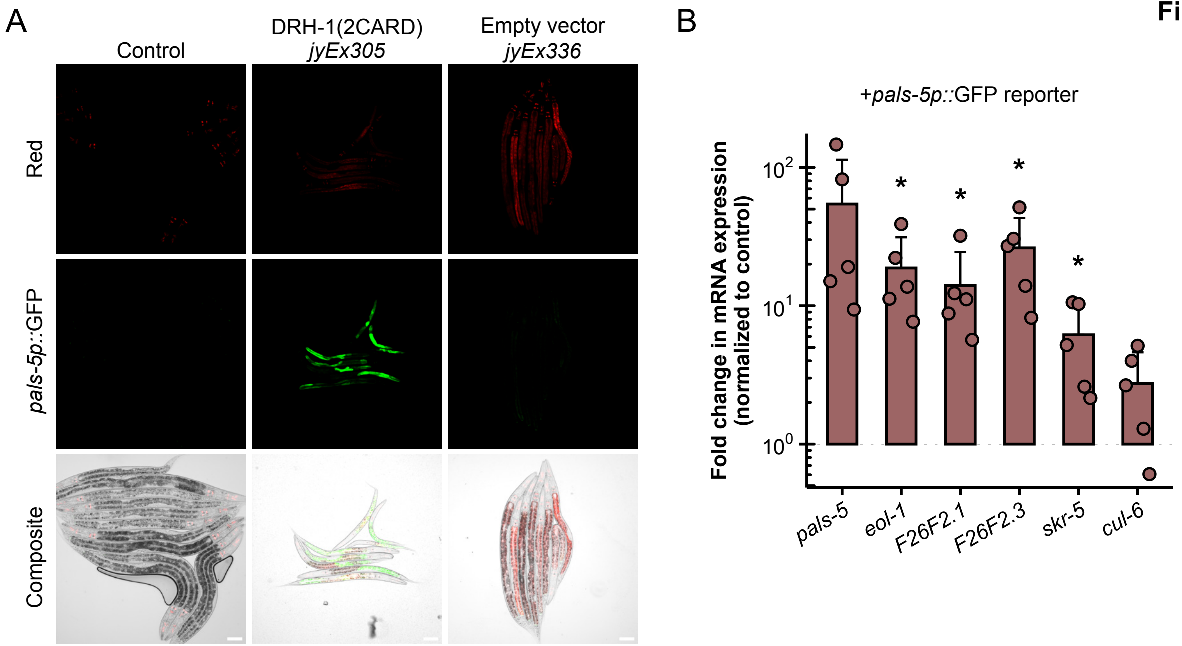
A
Control
DRH-1(2CARD)
jyEx305
Empty vector
jyEx336
Red
pals-5p::GFP
Composite
B
+pals-5p::GFP reporter
Fold change in mRNA expression
(normalized to control)
10^2
10^1
10^0
*
*
*
*
pals-5
eol-1
F26F2.1
F26F2.3
skr-5
cul-6

Figure S1

Figure S2

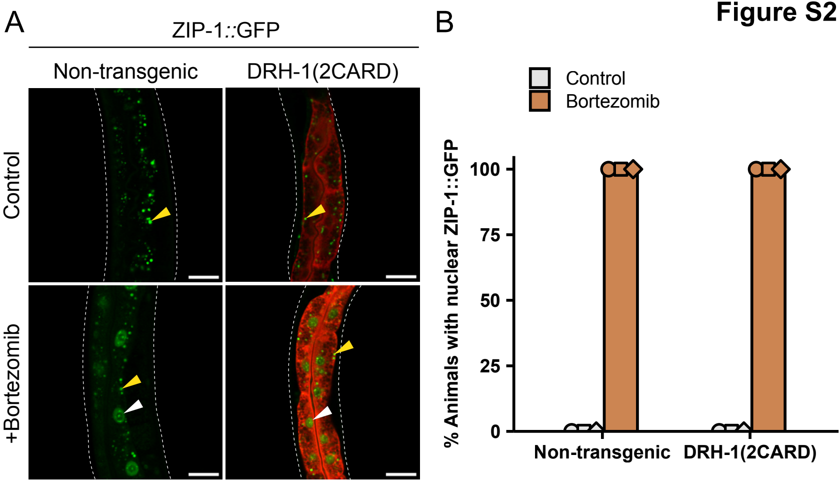

A
ZIP-1::GFP
Non-transgenic
DRH-1(2CARD)
Control
+Bortezomib
B
Control
Bortezomib
% Animals with nuclear ZIP-1::GFP
100
75
50
25
0
Non-transgenic
DRH-1(2CARD)

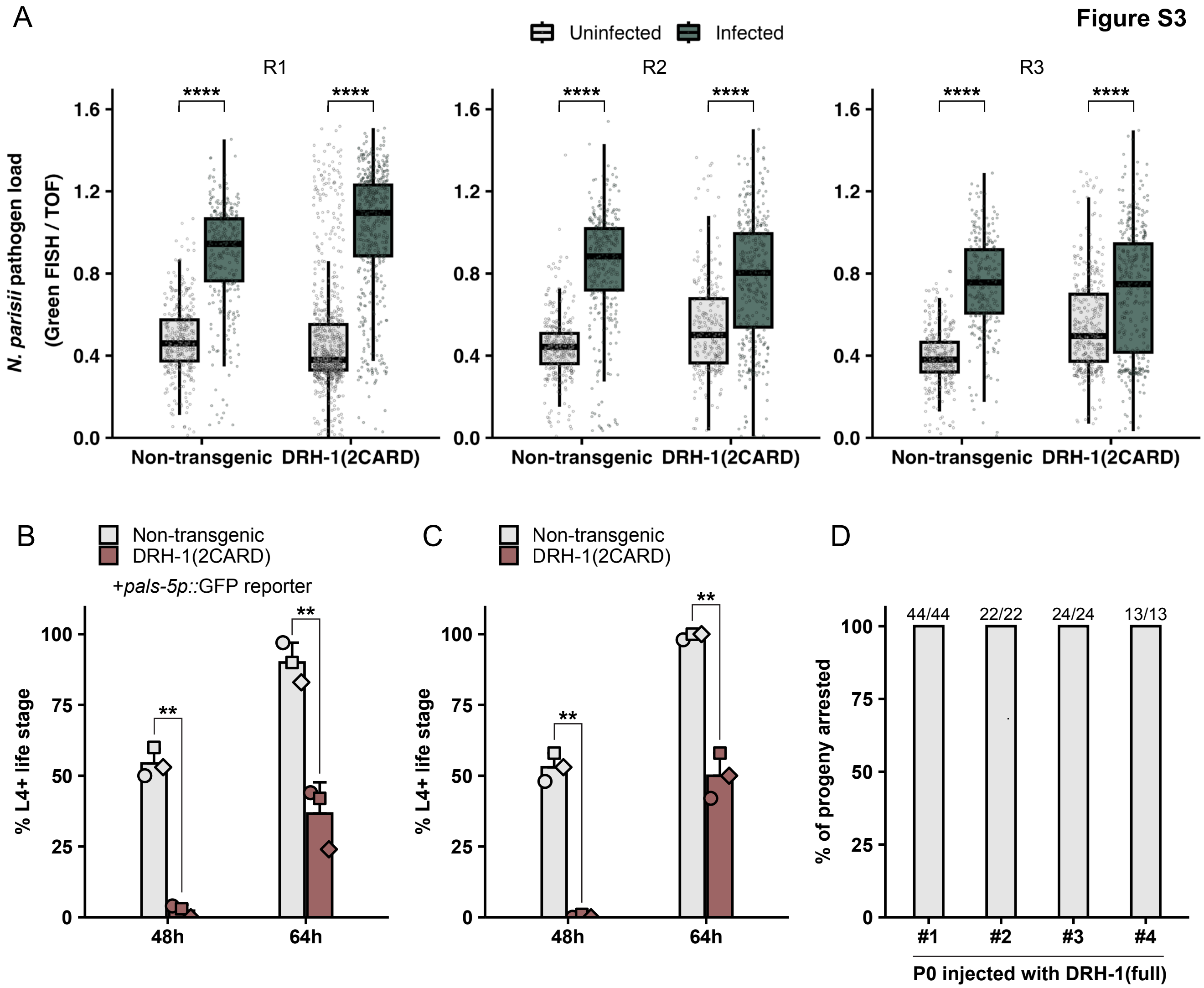
Figure S3
A
Uninfected
Infected
R1
R2
R3
N. parisii pathogen load (Green FISH / TOF)
****
1.6
1.2
0.8
0.4
0.0
Non-transgenic
DRH-1(2CARD)
B
Non-transgenic
DRH-1(2CARD)
+pals-5p::GFP reporter
% L4+ life stage
**
100
75
50
25
0
48h
64h
C
Non-transgenic
DRH-1(2CARD)
D
% of progeny arrested
44/44
22/22
24/24
13/13
#1
#2
#3
#4
P0 injected with DRH-1(full)

Figure S4

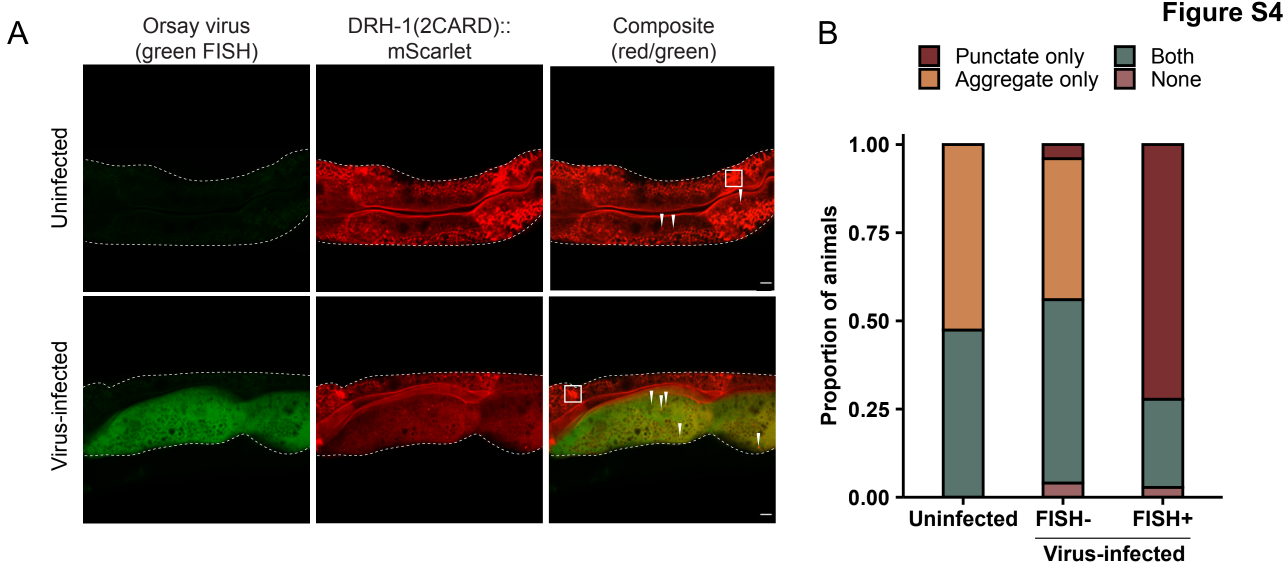
A
Orsay virus (green FISH)
DRH-1(2CARD):: mScarlet
Composite (red/green)
Uninfected
Virus-infected
B
Punctate only
Aggregate only
Both
None
Proportion of animals
1.00
0.75
0.50
0.25
0.00
Uninfected
FISH-
FISH+
Virus-infected

Figure S5

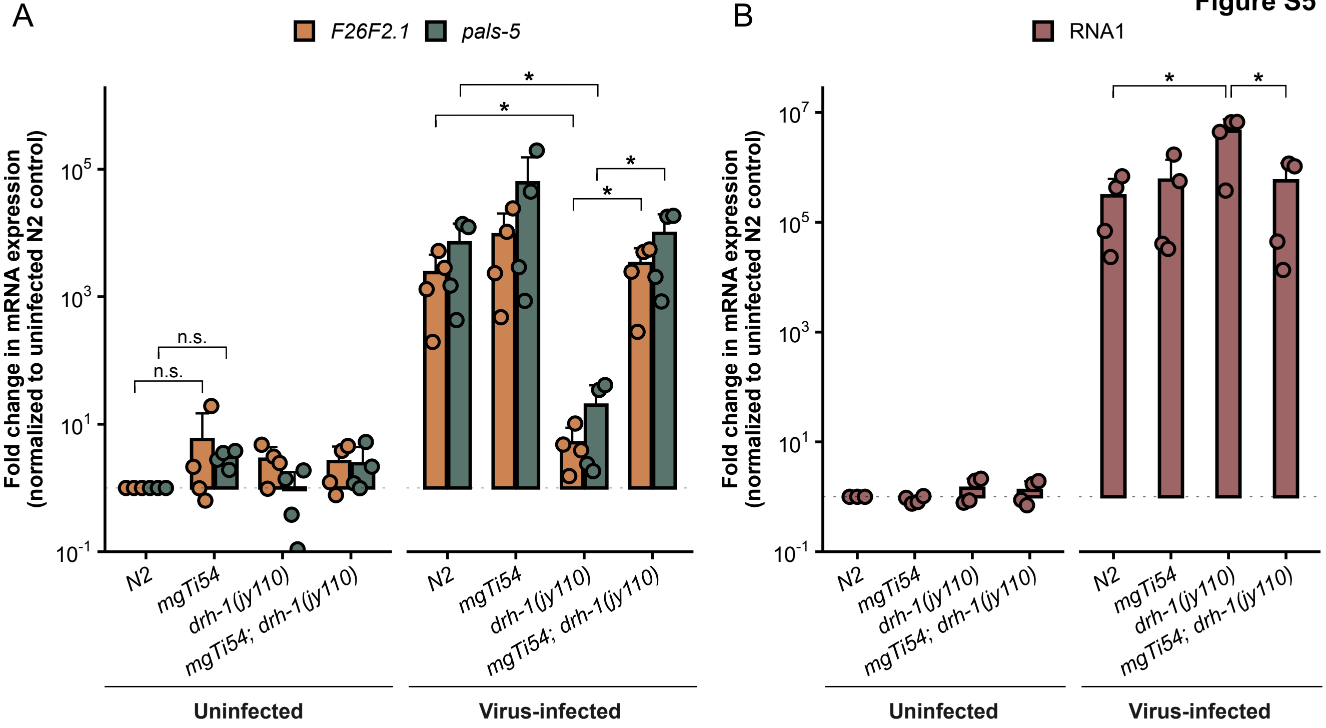
A
F26F2.1
pals-5
Fold change in mRNA expression
(normalized to uninfected N2 control)
10^5
10^3
10^1
10^-1
n.s.
n.s.
*
*
*
*
N2
mgTi54
drh-1(jy110)
mgTi54; drh-1(jy110)
Uninfected
Virus-infected
B
RNA1
10^7
10^5
10^3
10^1
10^-1
*
*
